# Optimized image segmentation using an improved reptile search algorithm with Gbest operator for multi-level thresholding

**DOI:** 10.1038/s41598-025-96429-1

**Published:** 2025-04-13

**Authors:** Laith Abualigah, Nada Khalil Al-Okbi, Saleh Ali Alomari, Mohammad H. Almomani, Sahar Moneam, Maryam A. Yousif, Vaclav Snasel, Kashif Saleem, Aseel Smerat, Absalom E. Ezugwu

**Affiliations:** 1 Computer Science Department, Al Al-Bayt University, Mafraq, 25113 Jordan; 2 Department of Computer Science, College of Science for Women, University of Baghdad, Baghdad, Iraq; 3 Faculty of Information Technology, Jadara University, Irbid, 21110 Jordan; 4 Faculty of Electrical Engineering and Computer Science, VŠB-Technical University of Ostrava, 70800 Poruba-Ostrava, Czech Republic; 5 Department of Computer Science & Engineering, College of Applied Studies & Community Service, King Saud University, 11362 Riyadh, Saudi Arabia; 6 Faculty of Educational Sciences, Al-Ahliyya Amman University, Amman, 19328 Jordan; 7 Centre for Research Impact & Outcome, Chitkara University Institute of Engineering and Technology, Chitkara University, Rajpura, Punjab 140401 India; 8 Unit for Data Science and Computing, North-West University, 11 Hofman Street, Potchefstroom, 2520 South Africa; 9 Department of Mathematics, Facility of Science, The Hashemite University, P.O box 330127, Zarqa 13133, Jordan; 10 Computer Technologies Engineering, Mazaya University College, Nasiriyah, Iraq

**Keywords:** Medical images, Image segmentation, Multi-level threshold, Reptile search algorithm, Otsu method, Kapur method, Medical research, Energy science and technology, Engineering, Mathematics and computing

## Abstract

Image segmentation using bi-level thresholds works well for straightforward scenarios; however, dealing with complex images that contain multiple objects or colors presents considerable computational difficulties. Multi-level thresholding is crucial for these situations, but it also introduces a challenging optimization problem. This paper presents an improved Reptile Search Algorithm (RSA) that includes a Gbest operator to enhance its performance. The proposed method determines optimal threshold values for both grayscale and color images, utilizing entropy-based objective functions derived from the Otsu and Kapur techniques. Experiments were carried out on 16 benchmark images, which included COVID-19 scans along with standard color and grayscale images. A thorough evaluation was conducted using metrics such as the fitness function, peak signal-to-noise ratio (PSNR), structural similarity index measure (SSIM), and the Friedman ranking test. The results indicate that the proposed algorithm seems to surpass existing state-of-the-art methods, demonstrating its effectiveness and robustness in multi-level thresholding tasks.

## Introduction

With the growing use of computer technologies, various challenging issues have emerged in areas like big data^1^, resource allocation^2^, the supply chain of large projects^3^, decision-making for cloud service providers^4^, environmental concerns^5^, photovoltaic models^6^, and image processing^7–9^. However, traditional methods such as gradient descent and other deterministic approaches often struggle to solve these complex problems^10,11^ effectively.

In fields like image processing and pattern recognition^10–12^, image segmentation plays a vital role^13–15^. The primary goal of segmentation is to differentiate between the foreground and background of an image^16,17^. It is a key task in image analysis. Segmentation involves dividing an image into distinct objects based on the intensity of their pixels and is utilized in various domains, including medicine, agriculture, and surveillance, among others^18–20^.

Numerous researchers have proposed a wide array of techniques for image segmentation, including thresholding, edge detection, graph partitioning, as well as methods based on clustering^21,22^. An important and simple method for image segmentation is thresholding^23^. Researchers widely use this technique due to its simplicity and efficiency^24–26^. The thresholding process relies on two main factors: the histogram and the number of thresholds applied to the image^27–29^. The histogram is crucial as it represents the probability distribution of the pixels^30,31^. Thresholding utilizes information from the histogram, applying a threshold (*th*) value based on a straightforward rule^32,33^.

There are two types of thresholds: bi-level and multi-layer^34,35^. A bi-level threshold uses a single value to divide an image into two sections^36–38^. In contrast, the multi-layer threshold specifies multiple values to identify how many objects are present in the image^39^. When color images are segmented into different regions based on color by using several threshold values, this is referred to as color image multilevel thresholding. However, determining the appropriate thresholds for color images can be challenging due to the 3D histogram involved, as opposed to the 1D histogram used for grayscale images^40,41^. Color images consist of components that vary based on both color and intensity growth, among others^42^.

The field of machine translation (MT) has been extensively researched, leading to the development of various algorithms aimed at determining optimal thresholds, which can be categorized into parametric and non-parametric methods^43,44^. Parametric techniques estimate the histogram using mathematical functions, with the Gaussian mixture being the most common approach^45^. In this method, the best thresholds are identified after accurately defining the set of functions that approximate the histogram. Non-parametric techniques, on the other hand, rely on statistical information from the classes to select ideal thresholds^46^. Among these, Otsu’s method^47^, Kapur’s method^48^, and the Tsallis entropy method are recognized as leading approaches in the field^49^.

Various applications, such as medical image processing^50^, satellite imagery^51^, synthetic aperture radar imagery^52^, and infrared image segmentation^53^, utilize different techniques to establish multi-level thresholds for effective image segmentation^54^. The Otsu method, introduced in 1979, aims to find an optimal threshold value that maximizes the variance between classes^55^. Kapoor’s method, proposed in 1985^56^, applies entropy to the histogram, while the Tsallis entropy method is grounded in the principle of moment preservation and is widely used for image thresholding segmentation^57^. Despite the multitude of proposed methods, the computational cost of the algorithms tends to increase with the number of levels, posing significant challenges for applications.

In recent decades, the increasing complexity of problems has highlighted the need for highly reliable techniques to enhance the performance of algorithms, particularly meta-heuristic optimization algorithms^58^. These techniques are known for their randomness and their ability to estimate optimal solutions for various optimization challenges, as well as their effectiveness in avoiding local optima, which makes them preferable to traditional optimization methods^59,60^. These algorithms aim to identify the best decision variables for a given problem by either minimizing or maximizing an objective function. However, they are often characterized by complex computations, lengthy processing times, and extensive search areas, especially when dealing with non-linear problems^61^.

Meta-heuristic optimization algorithms rely on two key tasks^62^: (1) Exploration^63^, which involves the algorithm’s ability to investigate non-local (global) areas, and (2) Exploitation, which refers to the algorithm’s ability to find better optimization solutions within those global areas^64^. Achieving a proper balance between exploration and exploitation is crucial. While all population-based optimization algorithms utilize these two strategies, their implementation can differ based on the specific mechanics and conditions of each algorithm^65^.

Among the key classifications that underpin meta-heuristics are swarm intelligence algorithms^66^, physics-based or human-based methods^67^, and evolutionary algorithms^68^. Evolutionary algorithms draw inspiration from natural evolution, utilizing biological factors to drive behaviors, such as mutation. The literature on Evolutionary Algorithms (EA) highlights various operators that are grounded in evolutionary theory principles. These operators include mutation, crossover, selection, and the inheritance of traits in offspring. One of the most well-known EAs is the Genetic Algorithm (GA), introduced by Holland, which is rooted in Darwinian evolutionary theory^69^. Another significant category is swarm algorithms, which many meta-heuristic approaches employ, often mimicking the movement patterns of animals. The key feature of this group is the way members share information about object movement within the swarm during the optimization process. Some algorithms in this category include the Salp Swarm, Krill Herd, and others^70^. Another type used by meta-heuristic algorithms is the physics-based approach. Notable algorithms in this area are the Imperialist Competitive Algorithm and the Teaching–Learning-Based Optimization Algorithm^71^. These algorithms mimic physical laws that we encounter in everyday life. The search solutions rely on principles that govern physical methods, as seen in the Gravitational Search Algorithm and the Charged System Search^72^. Lastly, there is the human-based method, which draws on human behavior in social contexts^73^.

Most published studies focus on modifying a specific algorithm, combining different algorithms, or introducing a new algorithm. The challenge of relying on a single algorithm stems from the understanding that no algorithm can effectively address all optimization problems for segmentation, as highlighted by the adage there is no free lunch. This limitation has led many researchers to explore these alternative approaches, which inspires our effort to implement a new optimization algorithm known as the Reptile Search Algorithm (RSA)^74^. The study proposes an enhanced Reptile Search Algorithm to determine optimal threshold values in images with varying levels of color and grayscale. This improved method incorporates the Gbest operator to address the key limitations found in the original version. These strategies were chosen based on their effectiveness in balancing exploration and exploitation, which is crucial for solving multi-level thresholding problems in image segmentation. Belly walking enhances the global search process by allowing broader coverage of the search space, ensuring that the algorithm avoids premature convergence. Coordination hunting, on the other hand, improves local exploitation by enabling collaborative decision-making among search agents, leading to a more refined and stable convergence toward optimal threshold values. This selection aligns with the problem’s complexity, ensuring that the proposed algorithm efficiently adapts to different image segmentation challenges. The entropy methods developed by Otsu and Kapur serve as the objective functions for this research. Sixteen benchmark images related to COVID-19, both in color and grayscale, were utilized for the experiments. The results were evaluated based on the fitness function, peak signal-to-noise ratio (PSNR), and structural similarity index (SSIM). The findings indicated that the proposed method surpassed several existing techniques.

The remainder of the paper is organized as follows: “Problem assessment of multilevel thresholding" discusses the challenges of multi-level image segmentation. “The proposed method (RSA)” section outlines the workflow of the proposed method. “Experiments and results” section presents the experiments and their results. Finally, “Conclusion” section concludes with a summary and suggestions for future work.

## Problem assessment of multilevel thresholding

Thresholding is an effective technique for image segmentation^23^. It categorizes all pixels in an image, whether in color or grayscale, into different classes^75,76^. A bi-level threshold, however, is not very effective for complex images since it only segments them into two objects. Therefore, it can be expanded to multi-level thresholding as follows:

- *C1 ← p if 0* ≤ *p* < *th1*
- *C2 ← p if th1* ≤ *p* < *th2*
- *Ci ← p if thi* ≤ *p* < *thi* + *1*
- *Cn ← p if thn* ≤ *p* < *L – 1*

where *th* (1,….,*k*) are several threshold values; in bi-level or multilevel thresholding, the goal is to identify the threshold values that effectively segment an image into different classes^54^. This paper focuses on two widely used methods: Otsu’s method and Kapur’s entropy^77^.

### Between-class variance method (Otsu’s method)

The search for optimal threshold values in Otsu’s method involves maximizing the between-class variance. Consider an image made up of *N* pixels, which are represented in *L* gray levels, with the number of pixels at gray level i denoted by *fi*^78^. The probability of gray level* i* is defined as shown in Eq. (1).

1 $${p}_{i}^{c}=\frac{{f}_{i}^{c}}{N}, {p}_{i}^{c}\ge 0, \sum_{i=0}^{L-1}{p}_{i}^{c}=1, c=\left\{\begin{array}{c}\text{1,2},3\, if\, RGB\, image \\ \text{1,2},3\, if\, gray \,scale \,image\end{array}\right..$$

In this context, *i* represents a gray level within the range of 0 to *L* − 1, while *c* denotes the image component. An RGB color image consists of three distinct components: red, green, and blue. Otsu’s method expands the concept of bi-level thresholding to accommodate multi-level thresholding^79,80^. When an image is divided into m classes, it will have *m* − 1 threshold values. The extended between-class variance function can be expressed as shown in Eq. (2):

2 $${f}^{c}\left(t\right)=\sum_{i=0}^{m-1}{\sigma }_{i}^{c}.$$

The optimal thresholding values ${(t}_{1}^{*c}{t}_{2}^{*c}{t}_{3}^{*c},\dots .,{t}_{m-1}^{*c})$ are calculated by maximizing ${\sigma }_{B}^{c}$ as in Eq. (3):

3 $${t}_{1}^{*c}{t}_{2}^{*c}{t}_{3}^{*c},\dots .,{t}_{m-1}^{*c}= \underset{0\le {t}_{1}^{c}\dots .\le {t}_{m-1}^{c}\le L-1}{\mathit{arg}}max\left\{{\sigma }_{B}^{c}\left({t}_{1}^{c},\right.\left.{t}_{2}^{c},\dots .,{t}_{m-1}^{c}\right).\right.$$

### Kapur’s entropy method

Kapur’s entropy standard relies on the probability distribution of the gray-level histogram^81^. The process of finding optimal threshold values using Kapur’s entropy involves maximizing entropy. This method was applied to determine the best threshold values for bi-level thresholding^82^. It was then expanded from bi-level to multilevel thresholding using Eq. (4).

4 $$f\left(t\right)=\sum_{i=0}^{m-1}{H}_{i}^{c}, c=\left\{\begin{array}{c}\text{1,2},3\, if \,RGB\, image \\ \text{1,2},3\, if\, gray \,scale\, image\end{array}\right..$$

In Eq. (4), the image is categorized into m classes using *m* − 1 threshold values. Kapur’s entropy can be readily adapted for multilevel image thresholding. The task of finding optimal multilevel thresholds is treated as a multi-dimensional optimization problem. To determine the *m* − 1 optimal threshold values (*t*1, *t*2,…, *tm* − 1) for a specific image, the objective function is maximized, as shown in Eq. (5).

5 $${(t}_{1}^{*c},{t}_{2}^{*c},\dots .,{t}_{m-1}^{*c})= \mathit{arg}\text{max}\left(\sum_{i=0}^{m-1}{H}_{i}^{c}\right).$$

## The proposed method (RSA)

### Biology and behavior of crocodiles algorithm (RSA)

The search algorithm for reptiles (RSA) draws inspiration from the natural behaviors of crocodiles, including their hunting strategies and social interactions^83^. The two main phases of crocodile activity—encircling and hunting prey—are referred to as exploration (global) and exploitation (local). These concepts are theoretically outlined and applied to develop the RSA for optimization tasks. As a population-based and gradient-free approach, RSA is suitable for tackling both simple and complex optimization challenges that have specific constraints. The cohesive groups formed by crocodiles enhance their effectiveness and facilitate active collaboration. Crocodiles possess streamlined bodies that enhance their speed, allowing for easier movement. When they walk, they lift their feet to the side, which contributes to their swiftness. Since animals typically navigate their environment by walking, the structure of their feet is a key characteristic. Below are the main features of the crocodile^84^.

- Vision: Because they are primarily nighttime hunters, crocodiles have excellent night vision.
- Hunting and diet: Crocodiles hunt for nearby fish or land animals before charging out to attack. Due to their slow metabolism, they can go for extended periods without eating. Crocodiles move quickly despite having the illusion of moving slowly.
- Locomotion: Crocodiles have a very high top speed over short distances. This is achieved by keeping its legs under its body in a straighter position (called the high walk). So, the walk will be at a high speed.
- Cognition: Crocodiles have some sophisticated cognitive abilities. They are able to identify and take advantage of prey behavior patterns.
- Hunting: Crocodiles cooperate to hunt, relying on both coordination and cooperation. It is thought to be unusual for an animal lacking a backbone to engage in coordinated hunting, which is an advanced kind of collaborative hunting in which certain predators coordinate with one another’s actions.
- Coordination and collaboration of crocodiles: According to a recent study, crocodiles hunt in packs. This made them stand out as one of the most sophisticated and intellectual teams that could foster cooperation among many individuals playing various roles. Their metabolism is slow, so they only occasionally eat. They nearly exclusively hunt at night and occasionally in shallow water.

The conclusion is that crocodiles are among the most cunning and skilled hunters, perhaps second only to humans. Crocodile behavior was treated as a mathematical optimization problem, and it involves deciding which option is best given a set of restrictions.

#### Initialization phase

In RSA, the optimization process starts with a set of randomly generated candidate solutions (*X*), as shown in Eq. (6), and the best solution found is considered to be approximately optimal in each iteration^85^.

6 $$X=\left[\begin{array}{ccc}\begin{array}{c}{x}_{\text{1,1} }\\ {x}_{\text{2,1} }\end{array}& \cdots & \begin{array}{c}\begin{array}{cc}{x}_{1,j }& {x}_{1,n}\end{array}\\ \begin{array}{cc}{x}_{2,j }& {x}_{2,n }\end{array}\end{array} \\ \vdots & \ddots & \vdots \\ \begin{array}{c}{x}_{N-\text{1,1}}\\ {x}_{N,1}\end{array}& \cdots & \begin{array}{c}\begin{array}{cc}{x}_{N-1,j}& {x}_{N-1,n-1}\end{array}\\ \begin{array}{cc}{x}_{N,j }& {x}_{N,n }\end{array}\end{array}\end{array}\right],$$

7 $${x}_{(i,j)}=rand+\left(UB-LB\right)+LB , J=1, 2, \dots ., n.$$

N represents the total number of potential solutions, and *n* indicates the magnitude of the given problem’s dimensions, *jth* position of the *ith* solution^86^.

#### Exploration strategy (encircling)

Crocodiles exhibit two main behaviors when surrounded: high walking and belly walking. Unlike other search phases, these movements make it difficult for them to get close to their intended prey due to the disturbances they create during the hunting phase. Through exploratory searching, a large area is uncovered, and after considerable effort, they may pinpoint a denser area. At this level of optimization, exploration techniques are also employed to aid in both the hunting and exploration phases through thorough and widespread searching.

The RSA can alternate between exploration (surrounding) and extraction (hunting) search terms based on four distinct characteristics. Its exploration mechanisms assess the search areas to find a more suitable response through two primary search strategies: the high-walking strategy and the belly-walking strategy. To move forward in this search phase, two conditions must be met. The high-walking movement strategy is applicable when *t* ≤ *T*/4, while the belly-walking movement strategy is relevant for *t* ≤ 2(*T*/4) and *t* > *T*/4. These steps are used in exploration searches. It should be noted that the stochastic scaling coefficient in Eq. (8) is examined to increase the variety of solutions and regions that can be explored.

8 $${x}_{(i,j)}\left(t+1\right)=\left\{\begin{array}{c}{best}_{j}\left(t\right)\times {\eta }_{(i,j)}\left(t\right) \times \beta -{R}_{\left(i,j\right)}\left(t\right)\times rand ,\, t\le \frac{T}{4} \\ {best}_{j}\left(t\right)\times {x}_{({r}_{1},j)} \times ES\left(t\right) \times rand,\, t\le 2\frac{T}{4}\, and\, t>\frac{T}{4}\end{array}\right..$$

#### Exploitation strategy (hunting)

Crocodiles primarily rely on coordination and collaboration as their main hunting strategies, which are evident in their hunting behavior. These strategies encompass various methods aimed at intensifying their search for prey. Unlike encircling tactics, crocodile hunting techniques—such as coordination and cooperation—enable them to target their prey due to their focused approach effectively. Consequently, their search for prey often leads to nearly optimal outcomes, albeit after several attempts. Furthermore, during this optimization phase, their exploitation methods are employed to conduct a more concentrated search near the ideal solution, emphasizing the importance of communication among them.

The RSA exploitation mechanisms utilize two primary search techniques—hunting coordination and hunting collaboration—to find the optimal solution. These search strategies are illustrated in Eq. (9). In this phase, the search process is influenced by the hunting coordination strategy, which is applicable when *t* ≤ 3 (*T*/4) and *t* > 2 (*T*/4). If these conditions are not met, the hunting cooperation strategy is employed, applicable when *t* ≤ *T* and *t* > 3 (*T*/4)^87^.

9 $${x}_{(i,j)}\left(t+1\right)=\left\{\begin{array}{c}{best}_{j}\left(t\right)\times {P}_{(i,j)}\left(t\right) \times rand ,\, t\le 3 \frac{T}{4} \,and\, t>2 \frac{T}{4} \\ {best}_{j}\left(t\right)-{\eta }_{\left(i,j\right)}\left(t\right)\times \epsilon - {R}_{\left(i,j\right)}(t)\times rand,\, t\le T \,and\, t>3\frac{T}{4}\end{array}\right..$$

### The reptile search algorithm (RSA)

The optimization procedure begins by producing a random group of candidate solutions in order to look into prospective locations for the nearly optimum answer. Every solution will trade places with the best-obtained solution at every iteration, according to the proposed RSA. The search techniques are split into two primary categories in order to emphasize exploration and exploitation (exploration and exploitation)^88^.

#### Strategy1: high walking

During the exploration phase, the Reptile identifies the prey area and selects the optimal hunting zone by employing a high walking movement strategy when *t* ≤ *T*/4, as described in Eq. (10).

10 $${x}_{(i,j)}\left(t+1\right)={best}_{j}\left(t\right)\times {\eta }_{(i,j)}\left(t\right) \times \beta -{R}_{\left(i,j\right)}\left(t\right)\times rand , t\le \frac{T}{4}.$$

In this context, the response from the initial search strategy at iteration t is represented by *x (i, j)(t* + *1).* The term *rand* refers to a random number between 0 and 1, while (*i*,) indicates the hunting operator for the *jt*ℎ position in the *it*ℎ solution, as defined by Eq. (11). Additionally, *Bestj (t*) represents the *jt*ℎ position in the best solution obtained so far. A critical parameter known as high walking influences the accuracy of exploration during the encircling phase across iterations, maintaining a fixed value of 0.1. The search region is narrowed using the reduction function ((*i*,)), which is established by Eq. (12). A random number is referred to as *Rand*. The phrases’ current iteration and the maximum number of iterations are abbreviated as *t* and *T*, respectively^89^.

11 $${\eta }_{\left(i,j\right)}\left(t\right)={best}_{j}\left(t\right)\times {P}_{\left(i,j\right)},$$

12 $${R}_{\left(i,j\right)}\left(t\right)=\frac{{best}_{j}\left(t\right)\times {x}_{(r2,j)}}{{best}_{j}\left(t\right)\times \epsilon },$$

Using Eq. (16) and as outlined in Approach 3*, P(i,j)* represents the percentage difference between the *jt*ℎ position of the optimal solution found and the *jt*ℎ position of the current solution.

#### Strategy2: belly walking exploration

When the prey area is located and *t* ≤ *T*/4, the Reptile employs a belly walking movement strategy to prepare the area and observe the prey, as described in Eq. (13).

13 $${x}_{(i,j)}\left(t+1\right)={best}_{j}\left(t\right)\times {x}_{({r}_{1},j)} \times ES\left(t\right) \times rand, t\le 2\frac{T}{4} \,and\, t>\frac{T}{4}.$$

When *r1* is a random value between (1 *N*), (*r*1,) indicates the random position of the *it*ℎ solution. *N* represents the total number of possible solutions. Equation (14) is utilized to calculate the probability ratio referred to as evolutionary sense ((*t*)), which randomly decreases from 2 to -2 throughout the repetitions.

14 $$ES\left(t\right)=2\times {r}_{3}\times \left(1-\frac{1}{T}\right).$$

At this level of optimization, both high-walking and belly-walking exploration methods are used to carry out thorough and widespread research that aids in the hunting (exploration) phases of the search process.

#### Strategy3: hunting of coordination exploitation

Candidate solutions begin an attempt to expand the search area when *t* ≤ *T*/2 and attempt to converge towards the near-optimal solution when *t* > *T*/2. When the prey area is precisely defined, the Reptile is ready to attack, and the hunting coordination strategy is performed when *t* ≤ 3 (*T*/4) and *t* > 2 (*T*/4), using Eq. (15).

15 $${x}_{(i,j)}\left(t+1\right)={best}_{j}\left(t\right)\times {P}_{(i,j)}\left(t\right) \times rand , t\le 3 \frac{T}{4} and t>2 \frac{T}{4}.$$

*Bestj(t)* represents the *jth* position in the best solution found so far, while *P(i,j)* indicates the percentage difference between the *jth* position of the best solution and the *jth* position of the current solution, calculated using Eq. (16).

16 $$P\left(t\right)=\alpha + \frac{{x}_{(i,j)}-{M}_{({x}_{i})}}{{best}_{j}\left(t\right)\times \left({UB}_{j}- {LB}_{j}\right)+\epsilon }.$$

In Eq. (16), (*xi*) indicates the average positions of the *it*ℎ solution as defined by Eq. (17). The upper and lower limits of the *jt*ℎ position are represented by (*j*) and (*j*), respectively. The parameter α, which is set at 0.1 for this study, plays a crucial role in determining the exploration accuracy (the variation between candidate solutions) during the collaborative hunting process throughout the iterations.

17 $${M}_{({x}_{i})}=\frac{1}{n} \sum_{j=1}^{n}{x}_{(i,j)}.$$

#### Strategy 4: hunting of cooperation exploitation

The hunting cooperation strategy is implemented when *t* ≤ *T* and *t* > 3 (*T*/4), as shown in Eq. (18). The RSA concludes once it meets the end criterion.

18 $${x}_{(i,j)}\left(t+1\right)={best}_{j}\left(t\right)-{\eta }_{\left(i,j\right)}\left(t\right)\times \epsilon - {R}_{\left(i,j\right)}\left(t\right)\times rand.$$

Hunting coordination and collaboration are strategies used by exploitation search systems to prevent getting trapped in local optima. These methods help maintain diversity among potential solutions while pinpointing the best answer. They defined two parameters (i.e., β and α) and conducted research during both the initial and final iterations to derive a stochastic value at each step. This approach is particularly beneficial when local optima become stagnant, especially in the later iterations.

#### Strategy5: Gbest method

The Gbest method is a variant of the Particle Swarm Optimization (PSO) algorithm frequently used to optimize specific objective functions^90,91^. This approach simulates a swarm of particles that navigate through the search space, influenced by the best position discovered up to that point.

In mathematical terms, each particle’s position in the swarm is denoted by a vector *xi*, while the objective function being optimized is represented as *f*(*x*). During each iteration, the velocity of each particle is adjusted based on its previous position, the global best position identified so far (referred to as *Gbest*), and a random value that encourages exploration^92^. The equation for updating the velocity of the *i-th* particle can be expressed as follows:

19 $$vi\left( {t\, + \,1} \right)\, = \,wvi\left( t \right)\, + \,c1 \, * \, rand1 \, * \, \left( {Pbesti \, - \, xi\left( t \right)} \right)\, + \,c2 \, * \, rand2 \, * \, \left( {Gbest \, - \, xi\left( t \right)} \right).$$

In this context, *w* represents the inertia weight, *c1* and *c2* are the acceleration coefficients, and rand1 and *rand2* are random values ranging from 0 to 1. *Pbesti* denotes the personal best position of the i-th particle, while *xi*(*t*) indicates the current position of the *i-th* particle at time step *t*^93,94^. The updated position of each particle is then calculated based on its velocity:

20 $$xi\left( {t + 1} \right) = xi\left( t \right) + vi\left( {t + 1} \right).$$

**Fig. 1:**
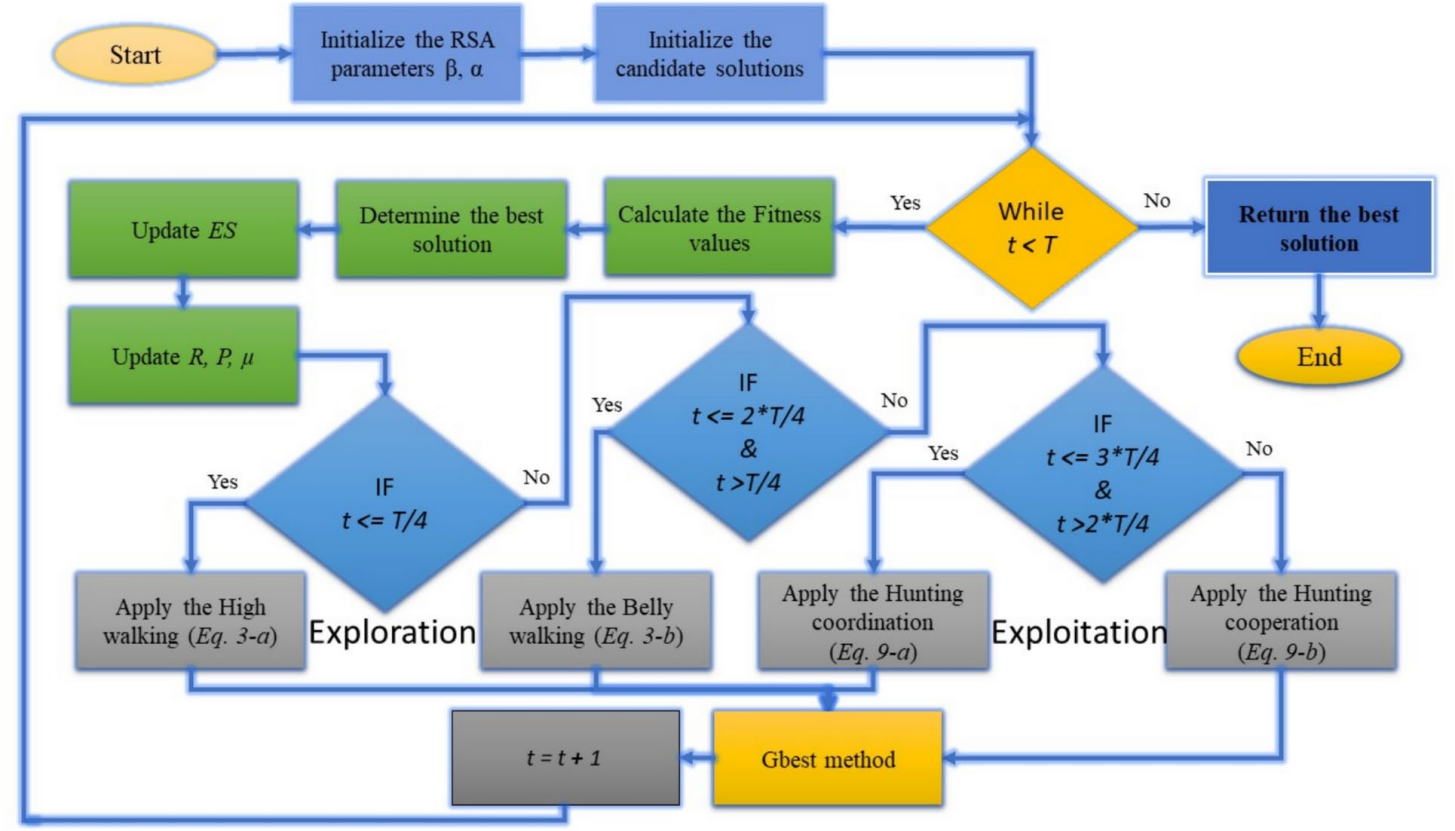
The main procedure of the proposed method.

The Gbest method proceeds until a stopping criterion is satisfied, which could be reaching a maximum number of iterations or identifying an acceptable solution. The primary procedure of the proposed method is illustrated in Fig. 1.

## Experiments and results

### Image dataset

**Fig. 2:**
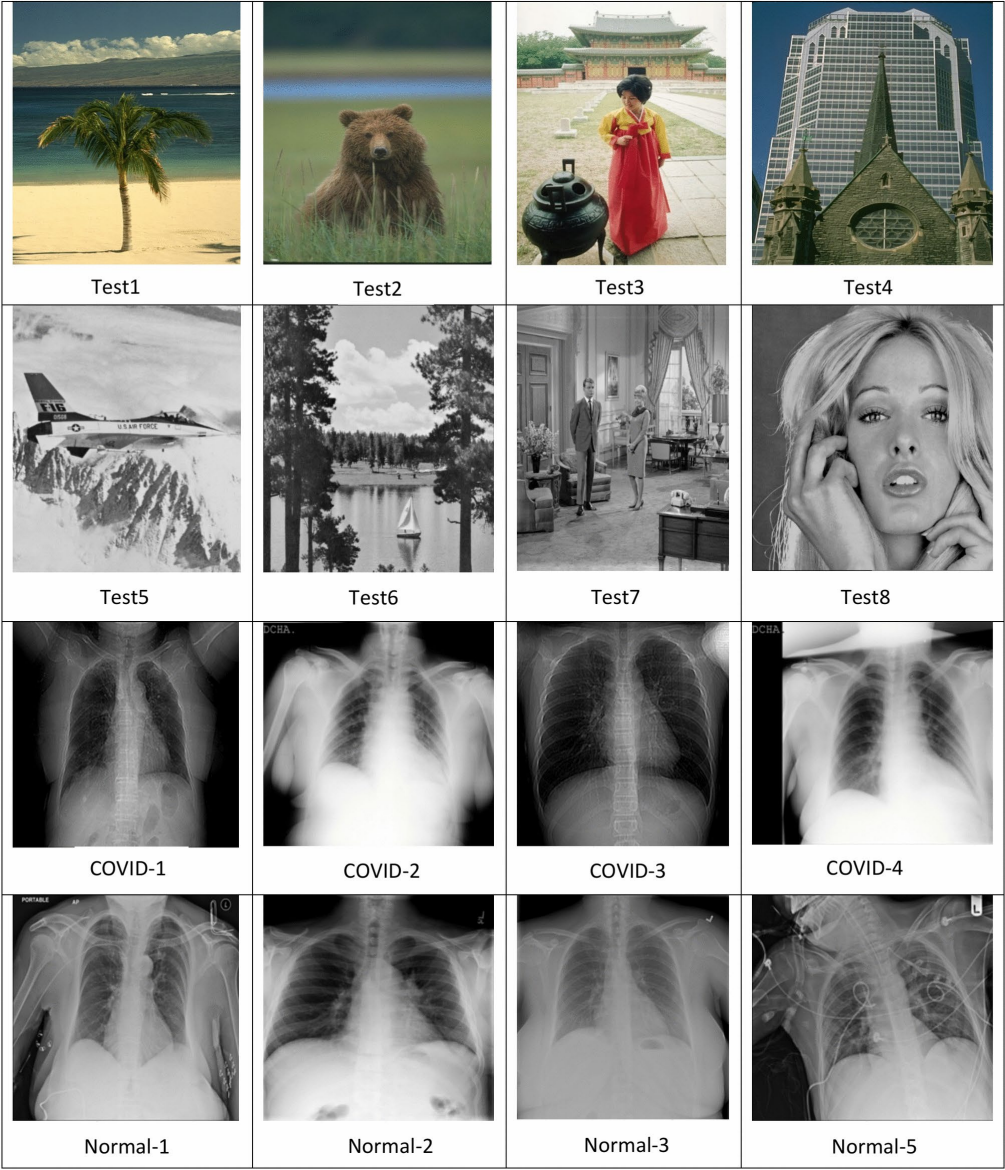
The benchmark image datasets.

The experiments were conducted on 16 well-known test images taken from https://r0k.us/graphics/kodak/, as illustrated in Fig. 2. In the image segmentation process using multilevel thresholding, the number of thresholds applied in these experiments was 3, 4, 5, and 6. Typically, the behavior of swarm intelligence optimization algorithms is random. Therefore, each experiment was repeated 100 times for every image and each threshold level.

### Experiment setup

Five different algorithms, Hybrid Marine Predators Algorithm (MPA)^95^, Self-adaptive Salp Swarm Algorithm (SSA)^96^, Improved Whale Optimization Algorithm (WOA)^97^, Dynamic Particle Swarm Optimization (PSO)^98^, Gaussian Aquila Optimizer (AO)^99^, are compared with the proposed algorithm (RSA) to validate its performance in deal with image segmentation problems. These algorithms have been used individually for a case study of chest X-ray Images for COVID-19 Cases(COVID-1, COVID-2, COVID-3, COVID-4), Normal Cases (NORMAL-1, NORMAL-2, NORMAL-3, NORMAL-4), Gray (Test5, Test6, Test7, Test8) and Color Images (Test1, Test2, Test3, Test4). Two well-known function methods, Otsu’s method, and Kapur’s entropy, are employed as objective functions. All the experiments executed in population size set to be 25, the maximum iteration is 100 with 30 Run time. The other parameter of each algorithm is shown in Table 1.

**Table 1:** Parameters of the comparison algorithms.

Algorithm	Parameters setting
SSA	c2 and c3 are random values (1, 0)
MPA	FADS = 0.2, P = 0.5
PSO	wMax = 0.9, wMin = 0.2, c1 = 2, c2 = 2
WOA	a (2,0), b = 1, t (− 1, 1)
AO	alpha = 0.1, delta = 0.1
RSA	β = 0.1, α = 0.1

### Segmented image quality measures

The results are shown as the mean, standard deviation, and results from the Friedman tests.

1. The Peak Signal-to-Noise Ratio (PSNR) is a measure used to assess the difference between a reference image and a segmented image, and it relies on the intensity values^100^.
  21 $$PSNR= 20{\text{log}}_{10}\left(\frac{255}{RMSE}\right), (in dB)$$
2. Root Mean Square Error (RMSE) refers to the root-mean-squared error, detected as:
  22 $$RMSE= \sqrt{\frac{{\sum }_{i=1}^{M}{\sum }_{j=1}^{Q}{(I\left(i,j\right)-Seg(i,j))}^{2}}{M*Q}}$$
  *I* and *Seg* denote the original and segmented images, which have dimensions *M* × *Q*, respectively. The highest value of PSNR indicates the optimal performance of the segmentation algorithms.
3. The Structural Similarity Index (SSIM): is used to evaluate the similarity between (*I*) and (*Seg*) images^101^, defined as:
  23 $$SSMI\left(I,Seg\right)= \frac{(2{\mu }_{I}{\mu }_{Seg}+{C}_{1})(2{\sigma }_{I.Seg}+{C}_{2})}{({\mu }_{I}^{2}+{\mu }_{Seg}^{2}+{C}_{1})({\sigma }_{I}^{1}+{\sigma }_{Seg}^{2}+{C}_{2})}$$
  *μ*_*I*_ and *μ*_*Seg*_ refer to the mean intensity of (*I*) and (*Seg*), respectively, while *σ*_*I*_ and *σ*_*Seg*_ refer to the standard deviation of (*I*) and (*Seg*), respectively; *σ*_*I*_, *Seg* represent a variance of (*I*) and (*Seg*). *c1* and *c2* are constants, that *c*_*1*_ = *6.5025* and *c*_*2*_ = *58.52252* measure. The maximum value of *SSIM* refers to a better performance.
4. The Friedman ranking test serves as a non-parametric alternative to the one-way ANOVA with repeated measures^102^. It is used to analyze changes across groups when the dependent variable is ordinal. Moreover, it can also be applied to continuous data that does not meet the assumptions necessary for conducting a one-way ANOVA with repeated measures.

### Experiment 1: maximizing between-class variance

In the first experiment, Otsu’s method (which focuses on between-class variance) served as the objective function that was maximized using the WOA, MPA, SSA, AO, RSA, and PSO algorithms. This was applied to chest X-ray images for COVID-19 cases, addressing the multilevel segmentation problem for both gray and color images to find optimal thresholding values. The method was evaluated on 16 test images, considering threshold values of 2, 3, 4, 5, and 6, along with the PSNR, SSIM, and Friedman ranking test values for the WOA, MPA, SSA, AO, RSA, and PSO algorithms.

The results of the comparative methods are shown in Table 2, where they are evaluated based on PSNR using the Otsu function. The findings indicate that the proposed method outperformed all other techniques, demonstrating its effectiveness in segmenting images and identifying optimal threshold values for accurate segmentation. The Friedman ranking test revealed that the proposed method achieved the top rank, followed by MPA, WOA, SSA, AO, and PSO. The evaluation focuses on key metrics such as the mean and standard deviation (STD) of the outcomes. These metrics provide valuable insights into both the central tendency and variability of the results, allowing for a better understanding of how the algorithms perform under various conditions. The mean values across different datasets show that SSA consistently outperforms the other algorithms in most cases, achieving the highest overall mean of 15.99876 among the six algorithms. This indicates that SSA has superior optimization capabilities, especially when dealing with the complexities of these datasets. RSA and MPA follow closely behind, with mean values of 15.28292 and 15.25353, respectively, reflecting strong but slightly less consistent performance compared to SSA.

**Table 2:** PSNR of Otsu function.

	K		AO	WOA	SSA	RSA	MPA	PSO
COVID-1	2	Mean	1.362E+01	1.335E+01	1.362E+01	1.534E+01	1.453E+01	1.347E+01
		STD	1.208E+00	2.880E+00	8.129E−01	7.494E−01	1.674E+00	1.055E+00
	3	Mean	1.386E+01	1.499E+01	1.568E+01	1.575E+01	1.395E+01	1.391E+01
		STD	1.412E+00	9.212E−01	1.990E+00	1.363E+00	1.214E+00	1.308E+00
	4	Mean	1.501E+01	1.637E+01	1.598E+01	1.524E+01	1.573E+01	1.673E+01
		STD	1.219E+00	7.225E−01	1.624E+00	1.679E+00	1.568E+00	1.304E+00
	5	Mean	1.618E+01	1.590E+01	1.513E+01	1.677E+01	1.586E+01	1.615E+01
		STD	3.787E−01	3.896E−02	3.291E−01	1.182E+00	2.173E+00	7.604E−01
	6	Mean	1.608E+01	1.968E+01	1.886E+01	1.966E+01	1.964E+01	1.880E+01
		STD	3.619E+00	9.446E−01	2.297E+00	6.667E−01	1.034E+00	1.210E+00
COVID-2	2	Mean	1.305E+01	1.074E+01	1.255E+01	1.370E+01	1.347E+01	1.366E+01
		STD	2.030E+00	1.366E+00	4.770E−01	2.426E−01	1.055E+00	2.046E+00
	3	Mean	1.529E+01	1.423E+01	1.415E+01	1.499E+01	1.391E+01	1.300E+01
		STD	8.957E−01	6.349E−01	2.905E+00	1.510E+00	1.308E+00	1.105E+00
	4	Mean	1.610E+01	1.680E+01	1.422E+01	1.681E+01	1.673E+01	1.440E+01
		STD	2.587E+00	1.131E+00	1.440E+00	9.076E−01	1.304E+00	3.323E+00
	5	Mean	1.671E+01	1.644E+01	1.473E+01	1.620E+01	1.615E+01	1.890E+01
		STD	1.482E+00	2.082E+00	7.197E−01	1.894E+00	7.604E−01	4.583E−01
	6	Mean	1.808E+01	1.818E+01	1.741E+01	1.882E+01	1.880E+01	1.799E+01
		STD	7.542E−01	1.306E+00	1.136E+00	3.056E+00	1.210E+00	6.474E−01
COVID-3	2	Mean	1.117E+01	1.083E+01	1.363E+01	1.370E+01	1.178E+01	1.339E+01
		STD	2.423E+00	1.894E+00	1.825E+00	1.966E+00	7.976E−01	2.060E+00
	3	Mean	1.451E+01	1.458E+01	1.537E+01	1.464E+01	1.421E+01	1.458E+01
		STD	2.245E+00	7.433E−01	2.144E+00	3.069E+00	2.253E+00	1.434E+00
	4	Mean	1.227E+01	1.334E+01	1.483E+01	1.627E+01	1.647E+01	1.621E+01
		STD	5.378E+00	1.033E+00	1.313E+00	9.904E−01	1.321E+00	2.464E+00
	5	Mean	1.692E+01	1.655E+01	1.570E+01	1.894E+01	1.753E+01	1.626E+01
		STD	2.173E+00	5.951E−01	4.706E+00	2.842E+00	1.362E+00	1.323E+00
	6	Mean	1.967E+01	1.728E+01	1.761E+01	1.993E+01	1.867E+01	1.634E+01
		STD	2.077E−01	2.821E+00	3.020E+00	3.267E+00	1.189E+00	3.535E+00
COVID-4	2	Mean	1.365E+01	1.178E+01	1.062E+01	1.485E+01	1.258E+01	1.407E+01
		STD	2.075E+00	7.976E−01	2.657E+00	2.158E+00	1.754E+00	5.943E−01
	3	Mean	1.489E+01	1.421E+01	1.496E+01	1.507E+01	1.397E+01	1.397E+01
		STD	1.755E+00	2.253E+00	4.609E−01	1.826E+00	7.742E−01	1.231E+00
	4	Mean	1.704E+01	1.647E+01	1.588E+01	1.725E+01	1.630E+01	1.664E+01
		STD	1.092E+00	1.321E+00	7.490E−01	1.485E+00	2.928E+00	1.689E+00
	5	Mean	1.674E+01	1.753E+01	1.573E+01	1.771E+01	1.665E+01	1.741E+01
		STD	1.736E+00	1.362E+00	1.112E+00	7.666E−01	8.058E−01	1.191E+00
	6	Mean	1.863E+01	1.867E+01	1.784E+01	1.932E+01	1.902E+01	1.877E+01
		STD	4.174E−01	1.189E+00	1.365E+00	2.666E+00	1.869E+00	1.075E+00
NORMAL-1	**2**	Mean	1.241E+01	1.320E+01	1.407E+01	1.364E+01	1.346E+01	1.288E+01
		STD	1.680E+00	3.673E−01	5.943E−01	7.395E−01	7.835E−01	2.084E+00
	3	Mean	1.280E+01	1.371E+01	1.397E+01	1.356E+01	1.353E+01	1.550E+01
		STD	4.886E−01	1.151E+00	1.231E+00	3.290E−01	2.547E−01	1.855E+00
	4	Mean	1.556E+01	1.682E+01	1.664E+01	1.673E+01	1.671E+01	1.392E+01
		STD	1.068E+00	5.721E−01	1.689E+00	1.715E+00	6.418E−01	3.240E+00
	5	Mean	1.753E+01	1.658E+01	1.741E+01	1.753E+01	1.574E+01	1.488E+01
		STD	1.052E+00	6.270E−01	1.191E+00	1.218E+00	2.035E+00	5.333E−01
	6	Mean	1.767E+01	1.797E+01	1.877E+01	1.793E+01	1.783E+01	1.398E+01
		STD	4.202E−01	8.214E−01	1.075E+00	2.094E−01	1.239E+00	3.625E+00
NORMAL-2	2	Mean	9.756E−01	1.227E+01	1.038E+01	1.214E+01	1.178E+01	1.284E+01
		STD	1.366E+01	1.058E+00	2.577E+00	2.731E+00	1.065E+00	7.942E−01
	3	Mean	6.879E−01	1.412E+01	1.357E+01	1.550E+01	1.178E+01	1.289E+01
		STD	1.526E+01	8.179E−01	1.144E+00	1.740E+00	1.787E+00	8.876E−01
	4	Mean	1.406E+00	1.696E+01	1.416E+01	1.453E+01	1.378E+01	1.728E+01
		STD	1.749E+01	2.445E+00	3.153E−01	1.405E+00	2.866E+00	3.646E−01
	5	Mean	1.372E+00	1.800E+01	1.591E+01	1.649E+01	1.566E+01	1.747E+01
		STD	1.694E+01	1.415E+00	1.400E+00	1.471E+00	3.699E−01	9.704E−01
	6	Mean	5.424E−01	1.842E+01	1.759E+01	1.770E+01	1.458E+01	1.833E+01
		STD	1.339E+00	1.668E+00	2.803E+00	4.557E−01	2.384E+00	1.596E+00
NORMAL-3	2	Mean	1.442E+01	1.238E+01	1.435E+01	1.415E+01	1.213E+01	1.079E+01
		STD	7.381E−01	1.580E+00	2.880E−01	6.727E−01	2.750E+00	1.588E+00
	3	Mean	1.543E+01	1.380E+01	1.502E+01	1.565E+01	1.518E+01	1.458E+01
		STD	8.607E−01	9.848E−01	2.063E+00	1.241E+00	8.002E−01	8.821E−01
	4	Mean	1.705E+01	1.772E+01	1.601E+01	1.651E+01	1.687E+01	1.575E+01
		STD	2.544E+00	1.519E+00	1.267E+00	1.243E+00	2.000E+00	7.981E−01
	5	Mean	1.706E+01	1.782E+01	1.802E+01	1.881E+01	1.877E+01	1.533E+01
		STD	8.481E−01	3.093E−01	1.068E+00	1.323E+00	1.033E+00	4.403E−01
	6	Mean	1.657E+01	1.928E+01	1.729E+01	1.843E+01	1.848E+01	1.792E+01
		STD	7.614E−01	9.202E−01	4.339E−01	6.957E−01	3.023E−02	4.327E−01
NORMAL-4	2	Mean	1.297E+01	1.178E+01	1.229E+01	1.395E+01	1.310E+01	1.365E+01
		STD	1.556E+00	7.976E−01	1.863E+00	2.355E+00	5.441E−01	2.075E+00
	3	Mean	1.452E+01	1.421E+01	1.456E+01	1.464E+01	1.427E+01	1.489E+01
		STD	1.431E+00	2.253E+00	7.746E−01	1.238E+00	1.420E+00	1.755E+00
	4	Mean	1.577E+01	1.747E+01	1.627E+01	1.781E+01	1.587E+01	1.704E+01
		STD	1.486E+00	1.321E+00	1.136E+00	1.513E+00	8.759E−01	1.092E+00
	5	Mean	1.800E+01	1.783E+01	1.717E+01	1.814E+01	1.775E+01	1.674E+01
		STD	1.088E+00	1.362E+00	6.481E−01	1.142E+00	2.209E+00	1.736E+00
	6	Mean	1.926E+01	1.867E+01	1.843E+01	1.965E+01	1.839E+01	1.863E+01
		STD	2.010E+00	1.189E+00	2.347E−01	1.552E+00	1.851E+00	4.174E−01
Test1	2	Mean	1.407E+01	1.354E+01	1.062E+01	1.429E+01	1.258E+01	1.208E+01
		STD	5.943E−01	7.399E−01	2.657E+00	1.267E+00	1.754E+00	1.324E+00
	3	Mean	1.397E+01	1.354E+01	1.496E+01	1.634E+01	1.397E+01	1.275E+01
		STD	1.231E+00	1.710E+00	4.609E−01	7.943E−01	7.742E−01	1.464E+00
	4	Mean	1.664E+01	1.769E+01	1.588E+01	1.776E+01	1.630E+01	1.442E+01
		STD	1.689E+00	1.905E+00	7.490E−01	3.073E+00	2.928E+00	6.635E−01
	5	Mean	1.844E+01	1.729E+01	1.573E+01	1.851E+01	1.665E+01	1.676E+01
		STD	1.191E+00	5.967E−01	1.112E+00	1.107E+00	8.058E−01	2.000E+00
	6	Mean	1.929E+01	1.937E+01	1.784E+01	1.977E+01	1.902E+01	1.747E+01
		STD	1.075E+00	3.842E−01	1.365E+00	5.507E−01	1.869E+00	1.458E+00
Test2	2	Mean	1.366E+01	1.483E+01	1.407E+01	1.510E+01	1.313E+01	1.270E+01
		STD	1.366E+00	2.130E+00	2.477E+00	2.151E+00	2.476E+00	1.789E+00
	3	Mean	1.380E+01	1.412E+01	1.403E+01	1.428E+01	1.421E+01	1.266E+01
		STD	1.257E+00	1.475E+00	2.507E+00	4.367E+00	1.038E+00	9.840E−01
	4	Mean	1.613E+01	1.562E+01	1.614E+01	1.410E+01	1.790E+01	1.546E+01
		STD	9.089E−01	1.624E+00	3.201E+00	3.062E−01	3.329E+00	3.412E+00
	5	Mean	1.665E+01	1.729E+01	1.708E+01	1.821E+01	1.688E+01	1.562E+01
		STD	1.363E+00	2.715E+00	3.906E−01	2.554E+00	9.949E−01	1.615E+00
	6	Mean	1.922E+01	1.894E+01	1.927E+01	1.759E+01	1.845E+01	1.866E+01
		STD	1.434E+00	1.740E+00	2.096E+00	1.595E+00	9.278E−01	1.145E+00
Test3	2	Mean	1.272E+01	1.134E+01	1.084E+01	1.212E+01	1.362E+01	1.177E+01
		STD	5.245E−01	1.783E+00	9.006E−01	1.095E+00	8.129E−01	1.201E+00
	3	Mean	1.476E+01	1.285E+01	1.277E+01	1.322E+01	1.568E+01	1.214E+01
		STD	1.512E+00	1.138E+00	9.075E−01	1.624E+00	1.990E+00	1.015E+00
	4	Mean	1.426E+01	1.431E+01	1.252E+01	1.333E+01	1.598E+01	1.338E+01
		STD	1.023E+00	1.236E+00	2.610E+00	2.472E−01	1.624E+00	1.944E+00
	5	Mean	1.583E+01	1.664E+01	1.599E+01	1.535E+01	1.513E+01	1.572E+01
		STD	1.714E+00	9.249E−01	1.449E+00	1.494E+00	3.291E−01	2.198E+00
	6	Mean	1.700E+01	1.758E+01	1.566E+01	1.570E+01	1.886E+01	1.587E+01
		STD	1.278E+00	1.903E+00	3.540E−01	1.868E+00	2.297E+00	1.363E+00
Test4	2	Mean	1.468E+01	1.352E+01	1.325E+01	1.358E+01	1.421E+01	1.365E+01
		STD	1.454E+00	7.411E−01	9.792E−01	5.557E−01	1.419E+00	2.075E+00
	3	Mean	1.645E+01	1.593E+01	1.580E+01	1.402E+01	1.549E+01	1.489E+01
		STD	1.891E+00	2.268E+00	7.442E−01	7.157E−01	1.366E+00	1.755E+00
	4	Mean	1.671E+01	1.609E+01	1.705E+01	1.745E+01	1.462E+01	1.704E+01
		STD	6.173E−01	1.230E+00	2.075E+00	1.199E+00	1.196E+00	1.092E+00
	5	Mean	1.680E+01	1.611E+01	1.662E+01	1.886E+01	1.678E+01	1.674E+01
		STD	1.574E+00	1.139E+00	5.558E−01	2.364E+00	6.008E−01	1.736E+00
	6	Mean	1.827E+01	1.803E+01	1.821E+01	1.986E+01	1.990E+01	1.863E+01
		STD	2.400E+00	7.417E−01	1.896E+00	5.705E−01	9.242E−01	4.174E−01
Test5	2	Mean	1.407E+01	1.178E+01	1.062E+01	1.329E+01	1.258E+01	1.362E+01
		STD	5.943E−01	7.976E−01	2.657E+00	1.267E+00	1.754E+00	8.129E−01
	3	Mean	1.397E+01	1.421E+01	1.496E+01	1.634E+01	1.397E+01	1.568E+01
		STD	1.231E+00	2.253E+00	4.609E−01	7.943E−01	7.742E−01	1.990E+00
	4	Mean	1.664E+01	1.747E+01	1.588E+01	1.476E+01	1.630E+01	1.598E+01
		STD	1.689E+00	1.321E+00	7.490E−01	3.073E+00	2.928E+00	1.624E+00
	5	Mean	1.844E+01	1.783E+01	1.573E+01	1.741E+01	1.665E+01	1.513E+01
		STD	1.191E+00	1.362E+00	1.112E+00	1.107E+00	8.058E−01	3.291E−01
	6	Mean	1.929E+01	1.867E+01	1.784E+01	1.877E+01	1.902E+01	1.886E+01
		STD	1.075E+00	1.189E+00	1.365E+00	5.507E−01	1.869E+00	2.297E+00
Test6	2	Mean	1.500E+01	1.335E+01	1.362E+01	1.514E+01	1.453E+01	1.305E+01
		STD	4.578E−01	2.880E+00	8.129E−01	7.494E−01	1.674E+00	2.030E+00
	3	Mean	1.502E+01	1.499E+01	1.568E+01	1.572E+01	1.395E+01	1.529E+01
		STD	4.958E−01	9.212E−01	1.990E+00	1.363E+00	1.214E+00	8.957E−01
	4	Mean	1.519E+01	1.637E+01	1.598E+01	1.504E+01	1.573E+01	1.610E+01
		STD	4.683E−01	7.225E−01	1.624E+00	1.679E+00	1.568E+00	2.587E+00
	5	Mean	1.654E+01	1.590E+01	1.513E+01	1.677E+01	1.586E+01	1.627E+01
		STD	1.204E+00	3.896E−02	3.291E−01	1.182E+00	2.173E+00	1.482E+00
	6	Mean	1.829E+01	1.938E+01	1.886E+01	1.966E+01	1.964E+01	1.808E+01
		STD	1.275E+00	9.446E−01	2.297E+00	6.667E−01	1.034E+00	7.542E−01
Test7	2	Mean	1.365E+01	1.178E+01	1.062E+01	1.285E+01	1.258E+01	1.407E+01
		STD	2.075E+00	7.976E−01	2.657E+00	2.158E+00	1.754E+00	5.943E−01
	3	Mean	1.489E+01	1.421E+01	1.496E+01	1.507E+01	1.397E+01	1.397E+01
		STD	1.755E+00	2.253E+00	4.609E−01	1.826E+00	7.742E−01	1.231E+00
	4	Mean	1.704E+01	1.747E+01	1.588E+01	1.625E+01	1.630E+01	1.664E+01
		STD	1.092E+00	1.321E+00	7.490E−01	1.485E+00	2.928E+00	1.689E+00
	5	Mean	1.674E+01	1.783E+01	1.573E+01	1.791E+01	1.665E+01	1.741E+01
		STD	1.736E+00	1.362E+00	1.112E+00	7.666E−01	8.058E−01	1.191E+00
	6	Mean	1.863E+01	1.867E+01	1.784E+01	1.892E+01	1.902E+01	1.877E+01
		STD	4.174E−01	1.189E+00	1.365E+00	2.666E+00	1.869E+00	1.075E+00
Test8	2	Mean	1.326E+01	1.294E+01	1.183E+01	1.233E+01	1.418E+01	1.199E+01
		STD	1.578E+00	2.735E+00	1.819E+00	2.101E+00	1.156E+00	1.455E+00
	3	Mean	1.588E+01	1.595E+01	1.310E+01	1.451E+01	1.481E+01	1.569E+01
		STD	2.101E+00	1.967E+00	2.482E+00	1.978E+00	2.722E+00	2.517E+00
	4	Mean	1.589E+01	1.554E+01	1.390E+01	1.597E+01	1.494E+01	1.473E+01
		STD	2.928E+00	5.276E−01	3.002E+00	1.059E+00	3.071E+00	3.143E+00
	5	Mean	1.786E+01	1.671E+01	1.503E+01	1.498E+01	1.800E+01	1.641E+01
		STD	2.267E+00	9.309E−01	1.781E+00	5.403E+00	1.597E+00	4.123E+00
	6	Mean	1.752E+01	1.658E+01	1.647E+01	1.766E+01	1.493E+01	1.491E+01
		STD	2.549E+00	1.915E+00	1.547E+00	6.232E−01	2.681E+00	3.046E+00
Mean			15.14604	15.25353	15.18173	15.99876	15.28292	15.11392
Ranking			5	3	4	1	2	6

PSO in Table 2, while a well-known optimization algorithm, has the lowest overall mean performance at 15.11392. This could suggest that its default parameter settings or search dynamics are not well-aligned with the specific characteristics of the datasets assessed in this study. AO and WOA demonstrate similar performance levels, with mean values of 15.14604 and 15.18173, respectively, indicating they are moderately effective for the optimization tasks at hand.

Standard deviation values in Table 2 offer important insights into the reliability of these algorithms. A lower standard deviation indicates more consistent performance across different runs, while a higher value suggests greater variability. SSA not only achieves a high mean but also shows relatively low variability, as demonstrated by consistently moderate standard deviation values across various datasets. This stability reinforces its position as the most effective algorithm in this assessment. RSA and MPA show slightly higher variability in some datasets, which may imply occasional sensitivity to initial conditions or specific characteristics of the problems. In contrast, AO and WOA tend to have larger standard deviations, indicating that their performance can vary more significantly across different experimental conditions.

The performance trends across specific datasets (e.g., COVID-1, NORMAL-1) reveal some intriguing details. For instance, in COVID-3 in Table 2, SSA records the highest mean values in several instances. Still, its standard deviation is also notably higher, indicating that while it is effective, its performance can sometimes vary significantly. On the other hand, MPA tends to show a balance with moderate mean and standard deviation values, suggesting reliable performance without extreme fluctuations. The NORMAL-2 dataset brings another important insight: RSA achieves one of the highest mean values with a relatively low standard deviation, highlighting its potential effectiveness for this particular type of problem. In contrast, PSO and AO show less consistency on this dataset, as evidenced by their higher standard deviation values.

The ranking based on average performance clearly identifies SSA as the top algorithm, followed by MPA, WOA, RSA, AO, and PSO, in Table 2. This ranking offers a clear order of preference for selecting algorithms for optimization tasks relevant to this study. Nonetheless, the choice of algorithm should also take into account the unique characteristics of the dataset and the application’s tolerance for variability. In conclusion, Table 2 illustrates that SSA stands out as the most effective and stable optimization method among the six algorithms. However, selecting an algorithm should involve a careful balance between average performance and stability, as shown by the trends and standard deviation values specific to the dataset.

In Table 3, the results are presented in terms of SSIM using the Otsu function. These results further confirm that the proposed method delivered outstanding performance compared to the other methods, highlighting its capability in segmenting the tested images and determining the best threshold values for precise segmentation. SSIM is an important metric used for assessing image similarity and segmentation tasks, focusing on the preservation of structure. The analysis takes into account both the mean and standard deviation (STD) across different datasets, offering valuable insights into the effectiveness and reliability of each algorithm.

**Table 3:** SSIM of Otsu function.

	K		AO	WOA	SSA	RSA	MPA	PSO
COVID-1	2	Mean	4.256E−01	4.030E−01	3.608E−01	5.195E−01	4.447E−01	3.762E−01
		STD	5.706E−02	5.995E−02	1.344E−01	5.128E−02	2.398E−02	3.032E−02
	3	Mean	5.159E−01	4.438E−01	4.606E−01	5.426E−01	3.954E−01	5.322E−01
		STD	7.921E−02	4.070E−02	8.964E−02	4.113E−02	4.514E−02	7.088E−02
	4	Mean	4.939E−01	5.075E−01	5.369E−01	5.534E−01	4.755E−01	5.366E−01
		STD	1.051E−01	1.404E−02	1.726E−02	6.423E−02	2.118E−02	3.246E−02
	5	Mean	5.503E−01	5.217E−01	5.866E−01	5.522E−01	5.320E−01	5.346E−01
		STD	8.076E−02	4.483E−02	4.549E−03	4.010E−02	4.895E−02	2.550E−02
	6	Mean	5.621E−01	5.884E−01	6.466E−01	5.985E−01	5.766E−01	5.574E−01
		STD	8.872E−02	3.140E−02	3.232E−02	4.210E−02	5.202E−02	8.599E−02
COVID-2	2	Mean	4.500E−01	4.222E−01	4.605E−01	5.132E−01	4.297E−01	3.740E−01
		STD	8.097E−02	6.783E−02	2.403E−02	1.734E−02	3.407E−02	1.429E−01
	3	Mean	5.389E−01	4.148E−01	4.671E−01	5.484E−01	4.981E−01	5.307E−01
		STD	1.247E−01	1.758E−02	1.566E−02	4.907E−02	5.337E−03	1.252E−01
	4	Mean	5.852E−01	5.411E−01	5.332E−01	5.934E−01	4.639E−01	4.373E−01
		STD	1.046E−01	3.782E−02	5.675E−02	5.799E−02	2.138E−02	3.243E−01
	5	Mean	6.016E−01	6.067E−01	5.557E−01	6.072E−01	5.298E−01	6.365E−01
		STD	2.364E−02	2.334E−02	4.663E−02	2.086E−02	3.287E−02	6.288E−02
	6	Mean	5.659E−01	6.278E−01	5.919E−01	6.475E−01	5.428E−01	6.381E−01
		STD	1.010E−01	2.698E−02	1.944E−02	7.393E−02	5.491E−02	2.243E−02
COVID-3	2	Mean	4.396E−01	3.390E−01	4.959E−01	5.637E−01	5.092E−01	5.295E−01
		STD	7.320E−02	9.128E−02	8.073E−02	1.038E−01	1.717E−02	6.294E−02
	3	Mean	4.181E−01	5.518E−01	6.106E−01	6.266E−01	4.817E−01	4.558E−01
		STD	5.682E−02	2.217E−02	2.991E−02	1.249E−01	8.559E−02	2.736E−02
	4	Mean	4.804E−01	4.655E−01	5.235E−01	6.032E−01	5.297E−01	5.311E−01
		STD	6.380E−02	3.143E−02	4.938E−02	7.619E−02	1.250E−01	1.567E−01
	5	Mean	5.357E−01	5.894E−01	5.190E−01	6.429E−01	5.832E−01	6.130E−01
		STD	5.270E−02	1.043E−02	1.720E−01	9.153E−02	8.211E−02	2.742E−02
	6	Mean	5.975E−01	6.023E−01	6.317E−01	6.556E−01	5.766E−01	6.354E−01
		STD	6.656E−02	8.000E−02	7.387E−02	3.659E−02	6.343E−03	3.781E−02
COVID-4	2	Mean	4.260E−01	6.062E−01	4.598E−01	5.620E−01	3.771E−01	5.298E−01
		STD	5.663E−02	5.908E−02	4.038E−02	5.466E−02	1.461E−01	3.016E−02
	3	Mean	4.662E−01	6.750E−01	4.855E−01	6.958E−01	4.879E−01	5.380E−01
		STD	9.588E−02	3.140E−02	6.947E−02	1.755E−02	5.216E−02	1.529E−01
	4	Mean	4.009E−01	6.331E−01	4.054E−01	6.731E−01	4.493E−01	6.410E−01
		STD	4.060E−02	3.235E−02	3.233E−02	5.327E−02	6.955E−02	6.988E−02
	5	Mean	5.273E−01	6.946E−01	4.061E−01	7.205E−01	6.223E−01	6.547E−01
		STD	7.891E−02	5.463E−02	2.259E−01	2.314E−02	1.438E−02	4.456E−02
	6	Mean	5.298E−01	6.620E−01	4.682E−01	7.317E−01	5.220E−01	6.919E−01
		STD	8.838E−02	7.795E−02	1.947E−01	5.561E−02	6.061E−02	3.922E−02
NORMAL-1	2	Mean	5.627E−01	4.339E−01	5.816E−01	5.867E−01	4.394E−01	5.816E−01
		STD	3.275E−02	8.474E−02	7.720E−02	7.750E−02	7.274E−02	4.957E−02
	3	Mean	6.405E−01	4.952E−01	5.835E−01	6.584E−01	3.508E−01	5.912E−01
		STD	1.166E−01	9.026E−02	4.330E−02	5.935E−02	1.870E−01	4.803E−02
	4	Mean	6.408E−01	4.977E−01	5.951E−01	6.737E−01	4.329E−01	5.939E−01
		STD	2.529E−02	5.372E−02	4.977E−02	4.539E−02	7.460E−02	3.299E−02
	5	Mean	7.082E−01	5.868E−01	6.850E−01	7.105E−01	5.757E−01	7.033E−01
		STD	4.435E−02	4.358E−02	8.325E−02	1.591E−01	6.859E−02	2.935E−02
	6	Mean	6.973E−01	5.229E−01	7.227E−01	6.980E−01	4.264E−01	6.795E−01
		STD	2.486E−02	3.107E−02	2.756E−02	1.245E−01	1.609E−01	9.070E−02
NORMAL-2	2	Mean	5.289E−01	5.569E−01	4.702E−01	5.685E−01	4.439E−01	4.481E−01
		STD	1.450E−01	4.319E−02	2.095E−01	3.671E−02	6.460E−02	3.118E−02
	3	Mean	5.788E−01	6.233E−01	6.596E−01	6.715E−01	4.993E−01	4.419E−01
		STD	1.254E−01	6.517E−02	4.570E−02	4.855E−02	4.298E−02	4.066E−02
	4	Mean	6.608E−01	6.885E−01	7.006E−01	6.944E−01	5.858E−01	6.008E−01
		STD	8.913E−02	2.155E−02	1.263E−02	2.865E−02	4.424E−02	4.804E−02
	5	Mean	7.017E−01	6.938E−01	6.505E−01	7.117E−01	5.908E−01	5.714E−01
		STD	5.937E−02	6.836E−02	4.633E−02	3.446E−02	8.104E−02	8.115E−02
	6	Mean	7.501E−01	7.460E−01	7.491E−01	7.540E−01	6.108E−01	6.516E−01
		STD	2.415E−02	1.609E−02	6.160E−02	8.992E−02	2.117E−02	9.446E−02
NORMAL-3	2	Mean	4.401E−01	4.258E−01	3.610E−01	6.563E−01	5.256E−01	5.796E−01
		STD	1.082E−01	4.194E−02	2.443E−02	7.665E−02	8.419E−02	6.787E−02
	3	Mean	4.784E−01	5.182E−01	5.080E−01	6.609E−01	6.372E−01	6.591E−01
		STD	7.128E−02	4.151E−02	5.054E−02	3.671E−02	1.771E−02	4.367E−02
	4	Mean	4.579E−01	5.747E−01	5.076E−01	7.284E−01	7.237E−01	7.274E−01
		STD	2.995E−02	1.792E−02	6.425E−02	8.382E−02	3.544E−02	5.130E−02
	5	Mean	6.077E−01	5.827E−01	5.787E−01	7.083E−01	6.665E−01	6.716E−01
		STD	1.038E−02	4.417E−02	4.299E−02	9.463E−02	3.954E−02	8.848E−02
	6	Mean	6.210E−01	5.795E−01	5.772E−01	7.537E−01	7.459E−01	7.464E−01
		STD	8.017E−02	4.209E−02	7.569E−02	5.391E−02	7.307E−02	2.225E−02
NORMAL-4	2	Mean	3.675E−01	2.728E−01	4.404E−01	4.527E−01	3.571E−01	2.368E−01
		STD	5.595E−02	7.381E−02	5.492E−02	1.628E−02	4.894E−02	8.440E−02
	3	Mean	3.650E−01	2.791E−01	4.168E−01	4.735E−01	4.010E−01	2.899E−01
		STD	8.061E−02	1.164E−01	3.814E−02	8.571E−02	6.885E−02	9.564E−02
	4	Mean	5.340E−01	4.547E−01	4.780E−01	5.707E−01	5.107E−01	4.129E−01
		STD	2.117E−01	1.502E−01	1.243E−01	2.707E−02	2.497E−02	1.497E−01
	5	Mean	6.204E−01	5.573E−01	6.132E−01	6.301E−01	5.095E−01	3.518E−01
		STD	8.256E−02	4.613E−02	3.477E−02	5.841E−02	5.333E−02	1.745E−01
	6	Mean	5.568E−01	5.654E−01	5.892E−01	6.322E−01	6.126E−01	5.937E−01
		STD	1.950E−01	7.380E−02	2.912E−02	2.547E−02	7.962E−02	7.467E−02
Test1	2	Mean	4.632E−01	3.557E−01	4.511E−01	4.916E−01	2.700E−01	3.455E−01
		STD	4.775E−02	1.375E−01	4.175E−02	1.294E−01	1.442E−01	2.027E−01
	3	Mean	4.887E−01	4.842E−01	4.598E−01	4.992E−01	3.073E−01	3.953E−01
		STD	6.309E−02	7.188E−02	4.384E−02	6.345E−02	4.807E−02	1.673E−01
	4	Mean	5.665E−01	5.340E−01	5.305E−01	5.899E−01	5.740E−01	4.685E−01
		STD	1.622E−02	1.460E−01	3.545E−02	1.478E−01	2.685E−01	1.392E−01
	5	Mean	6.049E−01	5.863E−01	5.689E−01	6.325E−01	5.452E−01	5.110E−01
		STD	4.772E−02	2.605E−02	7.921E−02	1.385E−01	7.418E−02	1.327E−01
	6	Mean	6.111E−01	6.080E−01	5.235E−01	5.943E−01	4.715E−01	5.751E−01
		STD	8.404E−02	1.572E−02	8.376E−02	9.406E−02	1.221E−01	6.479E−02
Test2	2	Mean	3.482E−01	1.808E−01	3.311E−01	4.267E−01	4.041E−01	4.310E−01
		STD	1.175E−01	1.098E−01	5.904E−02	1.443E−01	4.914E−02	7.245E−02
	3	Mean	4.441E−01	4.346E−01	3.936E−01	4.626E−01	3.869E−01	4.541E−01
		STD	1.783E−01	4.734E−02	3.010E−01	1.051E−01	7.548E−02	9.148E−02
	4	Mean	4.560E−01	5.650E−01	3.560E−01	5.144E−01	5.125E−01	5.191E−01
		STD	7.796E−02	7.337E−02	1.921E−01	4.612E−02	1.248E−01	9.389E−02
	5	Mean	5.516E−01	5.609E−01	4.733E−01	6.588E−01	5.617E−01	5.671E−01
		STD	2.204E−01	1.158E−01	5.548E−02	4.000E−02	4.398E−02	4.986E−02
	6	Mean	5.851E−01	6.903E−01	6.035E−01	7.408E−01	6.903E−01	5.367E−01
		STD	1.060E−01	7.253E−02	8.794E−02	2.700E−02	7.507E−02	2.268E−01
Test3	2	Mean	5.069E−01	4.120E−01	4.801E−01	5.433E−01	4.588E−01	5.011E−01
		STD	3.744E−02	1.076E−01	8.353E−02	8.036E−02	1.188E−01	1.683E−02
	3	Mean	6.284E−01	5.789E−01	6.290E−01	5.848E−01	4.844E−01	5.700E−01
		STD	2.366E−02	8.141E−02	3.452E−02	1.022E−01	1.773E−01	8.195E−02
	4	Mean	5.481E−01	5.482E−01	6.014E−01	5.549E−01	4.910E−01	5.296E−01
		STD	4.451E−02	1.464E−01	3.854E−02	1.282E−01	1.644E−01	1.575E−01
	5	Mean	6.618E−01	6.329E−01	5.740E−01	6.902E−01	5.628E−01	6.373E−01
		STD	3.521E−02	3.055E−02	4.118E−02	7.853E−02	2.237E−02	6.589E−02
	6	Mean	6.297E−01	5.777E−01	6.003E−01	6.370E−01	6.345E−01	6.354E−01
		STD	9.201E−02	3.988E−02	4.023E−02	4.144E−02	9.315E−02	4.801E−02
Test4	2	Mean	3.961E−01	3.107E−01	3.024E−01	4.541E−01	3.242E−01	4.168E−01
		STD	9.199E−02	5.552E−02	9.746E−02	8.619E−02	1.077E−01	6.718E−02
	3	Mean	4.377E−01	4.152E−01	4.186E−01	4.588E−01	4.505E−01	3.575E−01
		STD	7.252E−02	3.173E−02	1.171E−01	8.675E−02	1.337E−01	8.884E−02
	4	Mean	5.036E−01	4.483E−01	5.094E−01	5.688E−01	4.709E−01	4.829E−01
		STD	4.115E−02	8.294E−02	6.673E−02	7.312E−02	1.725E−01	1.409E−01
	5	Mean	6.293E−01	6.578E−01	5.827E−01	6.707E−01	5.006E−01	6.026E−01
		STD	8.035E−02	8.685E−02	1.258E−01	1.122E−01	6.136E−02	7.533E−02
	6	Mean	6.888E−01	7.405E−01	6.722E−01	6.909E−01	5.031E−01	5.222E−01
		STD	5.379E−02	2.711E−02	1.145E−01	2.206E−01	1.833E−01	1.606E−01
Test5	2	Mean	2.950E−01	3.306E−01	2.725E−01	5.143E−01	3.373E−01	3.214E−01
		STD	1.075E−01	1.213E−01	1.589E−01	1.321E−01	8.646E−02	1.695E−02
	3	Mean	3.633E−01	3.308E−01	2.696E−01	6.386E−01	3.492E−01	3.834E−01
		STD	8.634E−02	7.776E−03	3.676E−02	8.969E−02	7.340E−02	8.652E−02
	4	Mean	4.658E−01	5.548E−01	4.635E−01	6.047E−01	5.381E−01	5.016E−01
		STD	1.418E−01	9.272E−02	6.892E−02	4.147E−02	4.183E−02	2.586E−02
	5	Mean	5.742E−01	4.868E−01	5.847E−01	5.363E−01	5.475E−01	5.673E−01
		STD	1.288E−01	1.707E−01	6.912E−02	1.761E−01	5.209E−02	5.485E−02
	6	Mean	5.395E−01	5.932E−01	6.116E−01	6.484E−01	5.891E−01	6.209E−01
		STD	4.285E−02	7.897E−02	3.107E−02	6.177E−03	6.961E−02	4.261E−02
Test6	2	Mean	1.577E−01	2.333E−01	1.637E−01	4.748E−01	1.635E−01	1.428E−01
		STD	4.916E−02	8.337E−02	7.395E−03	1.208E−02	6.956E−02	6.992E−02
	3	Mean	1.932E−01	2.812E−01	2.240E−01	5.387E−01	2.659E−01	2.308E−01
		STD	2.909E−02	1.700E−01	4.797E−02	3.989E−02	8.610E−02	6.379E−02
	4	Mean	2.551E−01	2.761E−01	2.392E−01	3.923E−01	3.034E−01	3.233E−01
		STD	8.807E−02	4.222E−02	1.141E−01	4.216E−02	3.324E−02	8.317E−02
	5	Mean	3.824E−01	3.506E−01	4.123E−01	4.812E−01	4.474E−01	4.442E−01
		STD	1.255E−01	6.480E−02	2.083E−02	8.851E−02	1.410E−01	8.006E−02
	6	Mean	3.927E−01	4.258E−01	3.614E−01	6.854E−01	4.993E−01	5.286E−01
		STD	1.511E−01	4.308E−02	5.015E−02	1.132E−02	1.139E−01	1.520E−01
Test7	2	Mean	4.511E−01	3.557E−01	4.404E−01	5.317E−01	5.324E−01	4.632E−01
		STD	4.175E−02	1.375E−01	5.492E−02	7.247E−02	9.546E−02	4.775E−02
	3	Mean	4.598E−01	4.842E−01	4.168E−01	5.569E−01	4.623E−01	5.387E−01
		STD	4.384E−02	7.188E−02	3.814E−02	1.332E−01	6.354E−02	6.309E−02
	4	Mean	5.305E−01	5.340E−01	4.780E−01	6.219E−01	5.997E−01	5.665E−01
		STD	3.545E−02	1.460E−01	1.243E−01	8.377E−02	1.478E−02	1.622E−02
	5	Mean	5.689E−01	5.863E−01	6.132E−01	6.684E−01	6.051E−01	6.049E−01
		STD	7.921E−02	2.605E−02	3.477E−02	9.300E−02	3.483E−02	4.772E−02
	6	Mean	5.235E−01	6.080E−01	5.892E−01	6.954E−01	6.936E−01	6.111E−01
		STD	8.376E−02	1.572E−02	2.912E−02	2.310E−02	4.711E−02	8.404E−02
Test8	2	Mean	4.810E−01	3.571E−01	4.641E−01	4.527E−01	5.273E−01	4.952E−01
		STD	4.343E−02	4.894E−02	6.199E−02	1.628E−02	1.196E−01	1.027E−01
	3	Mean	5.054E−01	4.010E−01	5.047E−01	4.735E−01	5.520E−01	5.302E−01
		STD	9.452E−02	6.885E−02	1.174E−01	8.571E−02	6.738E−02	1.158E−01
	4	Mean	5.612E−01	5.107E−01	6.133E−01	5.707E−01	5.797E−01	5.150E−01
		STD	1.125E−01	2.497E−02	1.242E−01	2.707E−02	4.713E−02	8.638E−02
	5	Mean	5.600E−01	5.095E−01	6.669E−01	6.301E−01	6.367E−01	6.126E−01
		STD	5.051E−02	5.333E−02	4.652E−02	5.841E−02	8.358E−02	1.668E−02
	6	Mean	6.591E−01	6.126E−01	6.604E−01	6.922E−01	6.717E−01	6.867E−01
		STD	2.615E−02	7.962E−02	4.414E−02	2.547E−02	6.893E−02	7.263E−02
		Mean	0.524671	0.524372	0.524769	0.577326	0.524061	0.519254
		Ranking	3	4	2	1	5	6

The mean SSIM values indicate that RSA is the top-performing algorithm in Table 3, achieving the highest average score (mean = 0.577326) across the datasets. This suggests that RSA excels in preserving structural similarity during segmentation. SSA follows closely behind (mean = 0.524769), demonstrating consistent and reliable segmentation results. In contrast, PSO has the lowest ranking (mean = 0.519254), pointing to its limitations in segmentation accuracy when compared to the other methods. AO, WOA, and MPA show similar average performances, with means of 0.524671, 0.524372, and 0.524061, respectively. Although these averages are close, a more detailed analysis of dataset-specific performance and standard deviations reveals differences in their effectiveness.

In Table 3, RSA consistently outperforms other algorithms in both the COVID and NORMAL datasets, achieving the highest SSIM values in most instances and demonstrating strong reliability with low standard deviations. In the COVID-1 dataset, RSA records a mean SSIM of 0.5985 at K = 6, with a low standard deviation of 0.0421, indicating stability, while SSA closely follows. In COVID-2, RSA leads again, but SSA and PSO show competitive performance; for instance, at K = 6, SSA achieves 0.5919, closely trailing RSA’s 0.6475, with SSA showing lower variability. For COVID-3, RSA dominates with mean SSIMs of 0.6032, 0.6429, and 0.6556 at K = 4, 5, and 6, respectively, while SSA maintains consistent stability. In COVID-4, RSA continues to perform robustly with mean SSIM values exceeding 0.6 for K ≥ 4, while WOA shows notable improvement at K = 6. Similarly, in the NORMAL datasets, RSA achieves a mean SSIM of 0.7105 at K = 5 in NORMAL-1 and continues to lead in NORMAL-2 with an SSIM of 0.754 at K = 6. Although SSA performs well across these datasets, MPA and PSO exhibit higher variability, which limits their reliability despite achieving competitive mean values in some cases. In NORMAL-3 and NORMAL-4, RSA maintains its lead, with mean SSIMs above 0.7 at K = 5 and K = 6, while SSA and PSO rank slightly lower due to increased variability. Overall, RSA’s consistent performance and low variability establish it as the most reliable algorithm across these datasets.

The analysis of standard deviation values reveals that RSA maintains stability across most datasets in Table 3, consistently showing low STD values. This suggests that it is robust in delivering reliable results. SSA also demonstrates stability, especially in COVID datasets, where its low variability makes it a dependable option for image segmentation tasks. On the other hand, AO and WOA perform well in certain datasets but show higher variability in others, indicating they may be sensitive to the characteristics of the dataset or the settings of the parameters. Although PSO has a lower average rank, it occasionally shows strengths in COVID datasets, indicating it could be effective with properly adjusted parameters.

Based on the average performance across all datasets, RSA takes the lead in Table 3, followed closely by SSA and AO. These rankings indicate that RSA is the go-to option for tasks that demand high structural fidelity and consistency. SSA stands out as a strong contender, particularly in complex datasets. AO, WOA, and MPA show moderate performance, occasionally excelling under certain conditions. Although PSO ranks last, it demonstrates potential for targeted optimizations. The results highlight RSA’s effectiveness and reliability for segmentation tasks that utilize the Otsu function. SSA provides competitive performance with robustness across various datasets. AO, WOA, and MPA yield satisfactory results but need further adjustments to ensure stability across different datasets. Despite its lower average rank, PSO shows promise in specific applications when optimized. These insights assist in choosing algorithms for SSIM-based segmentation tasks, underscoring the significance of both average performance and stability.

**Fig. 3:**
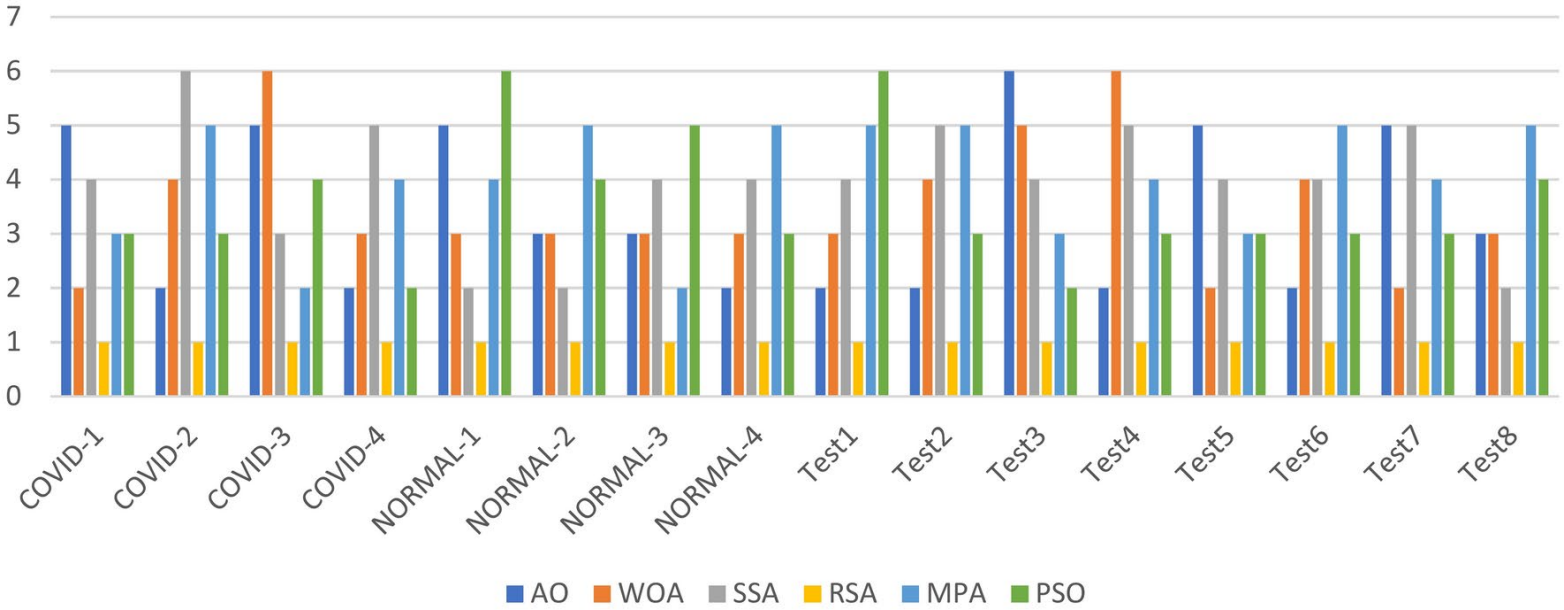
Friedman ranking test of PSNR using Otsu function.

The Friedman ranking test in Table 4 and its corresponding in Fig. 3 offer a detailed evaluation of six optimization methods based on their effectiveness in terms of Peak Signal-to-Noise Ratio (PSNR) across various datasets and segmentation granularity levels (K-values). The table outlines the ranking of each algorithm for specific datasets, with RSA consistently emerging as the top performer in most cases due to its strong capability to optimize PSNR, achieving the lowest overall mean rank in several tests. SSA, while generally competitive, often ranked second or third, indicating its reliability but slightly less consistency compared to RSA. AO and WOA showed mixed results, typically performing moderately well but falling short in more complex datasets. MPA and PSO received less favorable rankings overall, with higher mean ranks indicating their lower optimization efficiency. The accompanying figure illustrates the rankings across datasets, emphasizing RSA’s dominance, especially in the COVID and NORMAL datasets, and the varying performance of other methods. The figure effectively highlights the fluctuations in algorithm rankings, showcasing RSA’s consistent lead and SSA’s competitive standing, while other methods displayed significant variability in performance based on dataset characteristics.

**Table 4:** Friedman ranking test for the methods of PSNR for images experiment.

PSNR rank	K	AO	WOA	SSA	RSA	MPA	PSO
COVID1	2	3	6	4	1	2	5
	3	6	3	2	1	4	5
	4	6	2	3	5	4	1
	5	2	4	6	1	5	3
	6	6	2	5	1	3	4
	Sum	23	17	20	9	18	18
	Mean rank	4.6	3.4	4	1.8	3.6	3.6
	Final rank	5	2	4	1	3	3
COVID2	2	4	6	5	1	3	2
	3	1	3	4	2	5	6
	4	4	3	6	1	2	5
	5	2	3	6	4	5	1
	6	4	3	6	1	5	2
	Sum	15	18	27	9	20	16
	Mean rank	3	3.6	5.4	1.8	4	3.2
	Final rank	2	4	6	1	5	3
COVID3	2	5	6	2	1	4	3
	3	5	4	1	2	6	3
	4	6	5	4	2	1	3
	5	3	4	6	1	2	5
	6	2	5	4	1	3	6
	Sum	21	24	17	7	16	20
	Mean rank	4.2	4.8	3.4	1.4	3.2	4
	Final rank	5	6	3	1	2	4
COVID4	2	3	5	6	1	4	2
	3	3	4	2	1	5	6
	4	2	4	6	1	5	3
	5	4	2	6	1	5	3
	6	5	4	6	1	2	3
	Sum	17	19	26	5	21	17
	Mean rank	3.4	3.8	5.2	1	4.2	3.4
	Final rank	2	3	5	1	4	2
NORMAL-1	2	6	4	1	2	3	5
	3	6	3	4	2	5	1
	4	5	1	3	2	4	6
	5	1	4	3	2	5	6
	6	5	2	1	3	4	6
	Sum	23	14	12	11	21	24
	Mean rank	4.6	2.8	2.4	2.2	4.2	4.8
	Final rank	5	3	2	1	4	6
NORMAL-2	2	3	2	4	1	6	5
	3	4	3	2	1	5	6
	4	4	3	1	2	6	5
	5	2	3	4	1	5	6
	6	2	4	3	1	6	5
	Sum	15	15	14	6	28	27
	Mean rank	3	3	2.8	1.2	5.6	5.4
	Final rank	3	3	2	1	5	4
NORMAL-3	2	1	4	2	3	5	6
	3	2	6	4	1	3	5
	4	2	1	5	4	3	6
	5	5	4	3	1	2	6
	6	6	1	5	3	2	4
	Sum	16	16	19	12	15	27
	Mean rank	3.2	3.2	3.8	2.4	3	5.4
	Final rank	3	3	4	1	2	5
NORMAL-4	2	4	6	5	1	3	2
	3	3	6	4	2	5	1
	4	6	2	4	1	5	3
	5	2	3	5	1	4	6
	6	2	3	5	1	6	4
	Sum	13	14	18	5	20	14
	Mean rank	2.6	2.8	3.6	1	4	2.8
	Final rank	2	3	4	1	5	3
Test1	2	2	4	3	1	6	5
	3	2	3	4	1	6	5
	4	4	1	5	3	2	6
	5	2	3	4	1	5	6
	6	1	2	5	3	6	4
	Sum	11	13	21	9	25	26
	Mean rank	2.2	2.6	4.2	1.8	5	5.2
	Final rank	2	3	4	1	5	6
Test2	2	4	6	5	1	3	2
	3	3	6	4	2	5	1
	4	1	3	4	2	5	6
	5	2	3	5	1	4	6
	6	2	3	5	1	6	4
	Sum	12	21	23	7	23	19
	Mean rank	2.4	4.2	4.6	1.4	4.6	3.8
	Final rank	2	4	5	1	5	3
Test3	2	5	3	6	1	2	4
	3	6	4	5	1	3	2
	4	6	4	5	1	2	3
	5	2	6	1	5	4	3
	6	6	4	3	1	5	2
	Sum	25	21	20	9	16	14
	Mean rank	5	4.2	4	1.8	3.2	2.8
	Final rank	6	5	4	1	3	2
Test4	2	1	5	6	4	2	3
	3	1	2	3	1	6	5
	4	4	5	2	1	6	3
	5	2	6	5	1	3	4
	6	4	6	5	1	2	3
	Sum	12	24	21	8	19	18
	Mean rank	2.4	4.8	4.2	1.6	3.8	3.6
	Final rank	2	6	5	1	4	3
Test5	2	3	6	4	1	2	5
	3	6	3	2	1	4	5
	4	6	2	3	5	4	1
	5	2	4	6	1	5	3
	6	6	2	5	1	3	4
	Sum	23	17	20	9	18	18
	Mean rank	4.6	3.4	4	1.8	3.6	3.6
	Final rank	5	2	4	1	3	3
Test6	2	3	5	2	1	4	6
	3	2	6	5	1	4	3
	4	2	3	4	1	5	6
	5	4	3	5	2	6	1
	6	5	3	4	1	6	2
	Sum	16	20	20	6	25	18
	Mean rank	3.2	4	4	1.2	5	3.6
	Final rank	2	4	4	1	5	3
Test7	2	5	2	4	1	6	3
	3	6	2	5	1	4	3
	4	6	3	5	1	4	2
	5	5	2	6	1	4	3
	6	4	2	6	1	5	3
	SUM	26	11	26	5	23	14
	Mean rank	5.2	2.2	5.2	1	4.6	2.8
	Final rank	5	2	5	1	4	3
Test8	2	3	2	4	1	6	5
	3	4	3	2	1	5	6
	4	4	3	1	2	6	5
	5	2	3	4	1	5	6
	6	2	4	3	1	6	5
	Sum	15	15	14	6	28	27
	Mean rank	3	3	2.8	1.2	5.6	5.4
	Final rank	3	3	2	1	5	4

**Fig. 4:**
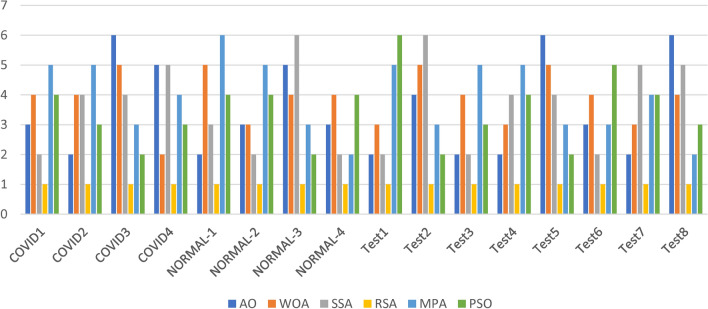
Friedman ranking test of SSIM using Otsu function.

The Friedman ranking test in Table 5 and Fig. 4 present a comparison of six optimization algorithms—Ant Optimization (AO), Whale Optimization Algorithm (WOA), Salp Swarm Algorithm (SSA), Random Search Algorithm (RSA), Modified Particle Swarm Optimization (MPA), and Particle Swarm Optimization (PSO)—in terms of their performance for SSIM (Structural Similarity Index Measure) during image segmentation experiments using the Otsu function. The rankings, derived from mean performance across various datasets and segmentation levels, consistently position RSA as the leading algorithm, showcasing the lowest mean rank in most datasets, which indicates its effectiveness in maximizing structural similarity. SSA often secures the second position, reflecting its competitive and dependable performance in maintaining SSIM, although other methods in certain tests occasionally outperform it. AO, WOA, and MPA show moderate rankings, with their performance varying across datasets, which suggests they may be sensitive to different conditions. PSO tends to rank lowest in most datasets, indicating its relative inefficiency in optimizing SSIM for these tasks. The figure visually reinforces these trends, with RSA consistently at the forefront across datasets, followed by SSA and AO. The fluctuations in rankings for WOA, MPA, and PSO point to the necessity for fine-tuning or exploring alternative strategies to enhance their performance. This analysis highlights RSA’s robustness and reliability as the best option for tasks that require high SSIM, with SSA emerging as a strong contender for consistent segmentation performance.

**Table 5:** Friedman ranking test for the methods of SSIM for images experiment.

SSIM rank	K	AO	WOA	SSA	RSA	MPA	PSO
COVID1	2	3	4	6	1	2	5
	3	3	5	4	1	6	2
	4	5	4	3	1	6	2
	5	2	5	1	3	4	6
	6	5	3	1	2	4	6
	Sum	18	21	15	8	22	21
	Mean rank	3.6	4.2	3	1.6	4.4	4.2
	Final rank	3	4	2	1	5	4
COVID2	2	3	5	2	1	4	6
	3	2	6	5	1	4	3
	4	2	3	4	1	5	6
	5	4	3	5	2	6	1
	6	5	3	4	1	6	2
	Sum	16	20	20	6	25	18
	Mean rank	3.2	4	4	1.2	5	3.6
	Final rank	2	4	4	1	5	3
COVID3	2	5	6	4	1	3	2
	3	6	3	2	1	4	5
	4	5	6	4	1	3	2
	5	5	3	6	1	4	2
	6	6	5	4	1	3	2
	Sum	27	23	20	5	17	13
	Mean rank	5.4	4.6	4	1	3.4	2.6
	Final rank	6	5	4	1	3	2
COVID4	2	5	2	4	1	6	3
	3	6	2	5	1	4	3
	4	6	3	5	1	4	2
	5	5	2	6	1	4	3
	6	4	2	6	1	5	3
	Sum	26	11	26	5	23	14
	Mean rank	5.2	2.2	5.2	1	4.6	2.8
	Final rank	5	2	5	1	4	3
NORMAL-1	2	4	6	2	1	5	3
	3	2	5	4	1	6	3
	4	2	5	4	1	6	3
	5	2	5	4	1	6	3
	6	3	5	1	2	6	4
	Sum	13	26	15	6	29	16
	Mean rank	2.6	5.2	3	1.2	5.8	3.2
	Final rank	2	5	3	1	6	4
NORMAL-2	2	3	2	4	1	6	5
	3	4	3	2	1	5	6
	4	4	3	1	2	6	5
	5	2	3	4	1	5	6
	6	2	4	3	1	6	5
	Sum	15	15	14	6	28	27
	Mean rank	3	3	2.8	1.2	5.6	5.4
	Final rank	3	3	2	1	5	4
NORMAL-3	2	4	5	6	1	3	2
	3	6	4	5	1	3	2
	4	6	4	5	1	3	2
	5	4	5	6	1	3	2
	6	4	5	6	1	3	2
	Sum	24	23	28	5	15	10
	Mean rank	4.8	4.6	5.6	1	3	2
	Final rank	5	4	6	1	3	2
NORMAL-4	2	3	5	2	1	4	6
	3	4	6	2	1	3	5
	4	2	5	4	1	3	6
	5	2	4	3	1	5	6
	6	6	5	4	1	2	3
	Sum	14	20	13	4	13	20
	Mean rank	2.8	4	2.6	0.8	2.6	4
	Final rank	3	4	2	1	2	4
Test1	2	2	4	3	1	6	5
	3	2	3	4	1	6	5
	4	3	4	5	1	2	6
	5	2	3	4	1	5	6
	6	1	2	5	3	6	4
	Sum	10	16	21	7	25	26
	Mean rank	2	3.2	4.2	1.4	5	5.2
	Final rank	2	3	2	1	5	6
Test2	2	4	6	5	2	3	1
	3	3	4	5	1	6	2
	4	5	1	6	3	4	2
	5	5	4	6	1	3	2
	6	5	3	4	1	2	6
	Sum	22	18	26	8	18	13
	Mean rank	4.4	3.6	5.2	1.6	3.6	2.6
	Final rank	4	5	6	1	3	2
Test3	2	2	6	4	1	5	3
	3	2	4	1	3	6	5
	4	4	3	1	2	6	5
	5	2	4	5	1	6	3
	6	4	6	5	1	3	2
	Sum	12	17	12	7	21	15
	Mean rank	2.4	3.4	2.4	1.4	4.2	3
	Final rank	2	4	2	1	5	3
Test4	2	3	5	6	1	4	2
	3	3	5	4	1	2	6
	4	3	6	2	1	5	4
	5	3	2	5	1	6	4
	6	3	1	4	2	6	5
	Sum	15	19	21	6	23	21
	Mean rank	3	3.8	4.2	1.2	4.6	4.2
	Final rank	2	3	4	1	5	4
Test5	2	5	3	6	1	2	4
	3	6	4	5	1	3	2
	4	6	4	5	1	2	3
	5	2	6	1	5	4	3
	6	6	4	3	1	5	2
	Sum	25	21	20	9	16	14
	Mean rank	5	4.2	4	1.8	3.2	2.8
	Final rank	6	5	4	1	3	2
Test6	2	3	5	2	1	4	6
	3	4	6	2	1	3	5
	4	2	5	4	1	3	6
	5	2	4	3	1	5	6
	6	6	5	4	1	2	3
	Sum	17	25	15	5	17	26
	Mean rank	3.4	5	3	1	3.4	5.2
	Final rank	3	4	2	1	3	5
Test7	2	2	4	3	1	6	5
	3	2	3	4	1	6	5
	4	4	2	5	3	1	6
	5	5	6	4	1	2	3
	6	3	4	6	2	5	1
	Sum	16	19	22	8	20	20
	Mean rank	3.2	3.8	4.4	1.6	4	4
	Final rank	2	3	5	1	4	4
Test8	2	4	6	5	2	1	3
	3	5	3	6	1	4	2
	4	5	4	6	1	2	3
	5	6	5	2	1	3	4
	6	6	4	5	1	2	3
	Sum	26	22	24	6	12	15
	Mean rank	5.2	4.4	4.8	1.2	2.4	3
	Final rank	6	4	5	1	2	3

### Experiment 2: maximizing Kapur’s entropy

The PSNR results of the Kapur function, as shown in Table 6, highlight the performance comparison of six. The data indicates that RSA consistently achieves the highest overall PSNR mean of 14.99, surpassing the other algorithms across different datasets and segmentation levels (K). Its strong performance is further validated by its stability, evidenced by a relatively low standard deviation in several cases.

**Table 6:** PSNR of Kapur function.

	K		AO	WOA	SSA	RSA	MPA	PSO
COVID-1	2	Mean	1.272E+01	1.134E+01	1.084E+01	1.312E+01	1.362E+01	1.177E+01
		STD	5.245E−01	1.783E+00	9.006E−01	1.095E+00	8.129E−01	1.201E+00
	3	Mean	1.476E+01	1.285E+01	1.277E+01	1.522E+01	1.568E+01	1.214E+01
		STD	1.512E+00	1.138E+00	9.075E−01	1.624E+00	1.990E+00	1.015E+00
	4	Mean	1.426E+01	1.431E+01	1.252E+01	1.533E+01	1.598E+01	1.338E+01
		STD	1.023E+00	1.236E+00	2.610E+00	2.472E−01	1.624E+00	1.944E+00
	5	Mean	1.583E+01	1.624E+01	1.599E+01	1.635E+01	1.513E+01	1.572E+01
		STD	1.714E+00	9.249E−01	1.449E+00	1.494E+00	3.291E−01	2.198E+00
	6	Mean	1.700E+01	1.758E+01	1.566E+01	1.770E+01	1.866E+01	1.587E+01
		STD	1.278E+00	1.903E+00	3.540E−01	1.868E+00	2.297E+00	1.363E+00
COVID-2	2	Mean	1.407E+01	1.178E+01	1.062E+01	1.329E+01	1.258E+01	1.362E+01
		STD	5.943E−01	7.976E−01	2.657E+00	1.267E+00	1.754E+00	8.129E−01
	3	Mean	1.397E+01	1.421E+01	1.496E+01	1.634E+01	1.397E+01	1.568E+01
		STD	1.231E+00	2.253E+00	4.609E−01	7.943E−01	7.742E−01	1.990E+00
	4	Mean	1.664E+01	1.747E+01	1.588E+01	1.676E+01	1.630E+01	1.598E+01
		STD	1.689E+00	1.321E+00	7.490E−01	3.073E+00	2.928E+00	1.624E+00
	5	Mean	1.844E+01	1.783E+01	1.573E+01	1.741E+01	1.665E+01	1.513E+01
		STD	1.191E+00	1.362E+00	1.112E+00	1.107E+00	8.058E−01	3.291E−01
	6	Mean	1.929E+01	1.867E+01	1.784E+01	1.877E+01	1.902E+01	1.886E+01
		STD	1.075E+00	1.189E+00	1.365E+00	5.507E−01	1.869E+00	2.297E+00
COVID-3	2	Mean	9.832E+00	1.201E+01	1.164E+01	1.282E+01	1.154E+01	9.259E+00
		STD	2.891E+00	2.712E+00	1.352E+00	9.497E−01	7.658E−01	1.138E+00
	3	Mean	1.117E+01	1.265E+01	1.407E+01	1.468E+01	1.276E+01	1.432E+01
		STD	2.668E+00	1.448E+00	1.866E+00	9.789E−01	2.895E+00	1.562E+00
	4	Mean	1.420E+01	1.286E+01	1.462E+01	1.518E+01	1.514E+01	1.383E+01
		STD	2.476E+00	2.234E+00	2.480E+00	9.905E−01	2.420E+00	3.181E+00
	5	Mean	1.670E+01	1.489E+01	1.471E+01	1.693E+01	1.514E+01	1.372E+01
		STD	6.884E−01	1.136E+00	6.094E−01	1.107E+00	2.744E+00	6.166E−01
	6	Mean	1.739E+01	1.706E+01	1.662E+01	1.944E+01	1.941E+01	1.572E+01
		STD	1.797E+00	2.543E+00	3.094E+00	1.390E+00	3.046E−01	1.466E+00
COVID-4	2	Mean	1.311E+01	1.301E+01	1.377E+01	1.387E+01	9.832E+00	1.308E+01
		STD	2.944E−01	1.685E+00	9.623E−01	1.058E+00	2.891E+00	7.793E−01
	3	Mean	1.324E+01	1.422E+01	1.360E+01	1.480E+01	1.115E+01	1.276E+01
		STD	6.121E−01	7.098E−01	1.586E+00	1.281E−01	2.643E+00	1.905E+00
	4	Mean	1.672E+01	1.499E+01	1.450E+01	1.691E+01	1.421E+01	1.571E+01
		STD	1.719E+00	8.064E−02	2.984E+00	2.346E+00	2.476E+00	1.637E+00
	5	Mean	1.660E+01	1.594E+01	1.600E+01	1.733E+01	1.670E+01	1.650E+01
		STD	3.155E−01	3.750E−01	3.089E−01	1.374E+00	6.741E−01	6.601E−01
	6	Mean	1.741E+01	1.648E+01	1.590E+01	1.759E+01	1.740E+01	1.605E+01
		STD	1.275E+00	3.175E+00	7.944E−01	2.001E+00	1.797E+00	2.022E+00
NORMAL-1	2	Mean	1.114E+01	1.126E+01	9.985E+00	1.281E+01	1.154E+01	9.912E+00
		STD	1.425E+00	7.011E−01	2.254E+00	3.366E+00	7.585E−01	1.741E+00
	3	Mean	1.210E+01	1.340E+01	1.291E+01	1.450E+01	1.266E+01	9.602E+00
		STD	2.016E+00	2.161E+00	3.424E+00	1.617E+00	2.886E+00	3.503E+00
	4	Mean	1.566E+01	1.421E+01	1.486E+01	1.554E+01	1.514E+01	1.375E+01
		STD	1.695E+00	3.085E+00	3.002E+00	2.534E+00	2.420E+00	5.038E+00
	5	Mean	1.502E+01	1.380E+01	1.710E+01	1.769E+01	1.514E+01	1.662E+01
		STD	2.498E+00	2.172E+00	2.744E+00	2.995E−01	2.744E+00	2.590E+00
	6	Mean	1.370E+01	1.650E+01	1.793E+01	1.836E+01	1.821E+01	1.677E+01
		STD	3.318E−01	2.378E+00	1.336E+00	1.005E+00	3.046E−01	6.589E−01
NORMAL-2	2	Mean	1.032E+01	1.092E+01	8.052E+00	1.125E+01	8.954E+00	9.690E+00
		STD	3.211E+00	3.751E−01	8.925E−01	4.922E−01	2.303E+00	4.256E+00
	3	Mean	1.249E+01	1.230E+01	1.258E+01	1.323E+01	1.126E+01	1.319E+01
		STD	1.722E+00	2.625E+00	7.959E−01	3.396E+00	6.225E+00	2.512E+00
	4	Mean	1.299E+01	1.509E+01	1.563E+01	1.571E+01	1.285E+01	1.392E+01
		STD	3.576E+00	1.223E−01	1.757E+00	1.978E+00	2.351E+00	1.513E+00
	5	Mean	1.834E+01	1.584E+01	1.630E+01	1.985E+01	1.461E+01	1.625E+01
		STD	1.096E+00	1.936E+00	1.836E+00	1.230E+00	3.441E+00	4.997E−01
	6	Mean	1.760E+01	1.790E+01	1.608E+01	1.860E+01	1.798E+01	1.661E+01
		STD	1.193E+00	1.174E+00	1.286E+00	7.104E−01	3.380E+00	2.495E+00
NORMAL-3	2	Mean	8.917E+00	1.145E+01	1.144E+01	1.208E+01	9.269E+00	1.105E+01
		STD	2.702E+00	4.326E−01	2.701E+00	7.257E−01	3.097E+00	2.033E+00
	3	Mean	1.343E+01	8.701E+00	1.233E+01	1.371E+01	1.067E+01	1.194E+01
		STD	1.634E+00	1.624E+00	1.646E+00	2.190E+00	1.303E+00	3.757E+00
	4	Mean	1.560E+01	1.593E+01	1.456E+01	1.663E+01	1.408E+01	1.533E+01
		STD	1.759E+00	3.107E+00	1.299E+00	2.676E+00	3.986E+00	8.586E−01
	5	Mean	1.536E+01	1.555E+01	1.559E+01	1.669E+01	1.475E+01	1.338E+01
		STD	1.223E+00	3.454E+00	3.565E+00	1.265E+00	3.765E+00	3.668E+00
	6	Mean	1.510E+01	1.765E+01	1.513E+01	1.860E+01	1.737E+01	1.620E+01
		STD	7.585E−01	1.452E+00	5.515E+00	2.715E+00	2.566E−01	3.012E+00
NORMAL-4	2	Mean	1.234E+01	1.066E+01	1.246E+01	1.338E+01	1.154E+01	1.155E+01
		STD	2.559E+00	6.439E−01	2.585E+00	4.467E+00	7.658E−01	3.294E+00
	3	Mean	1.229E+01	1.134E+01	9.728E+00	1.312E+01	1.271E+01	1.067E+01
		STD	1.990E+00	2.353E+00	2.528E+00	2.017E+00	2.886E+00	1.907E+00
	4	Mean	1.599E+01	1.325E+01	1.753E+01	1.777E+01	1.514E+01	1.555E+01
		STD	2.602E+00	1.881E+00	8.370E−01	2.585E+00	2.420E+00	1.197E+00
	5	Mean	1.624E+01	1.717E+01	1.723E+01	1.750E+01	1.513E+01	1.579E+01
		STD	1.412E+00	1.095E+00	9.301E−01	2.330E+00	2.740E+00	2.740E+00
	6	Mean	1.749E+01	1.687E+01	1.668E+01	1.951E+01	1.941E+01	1.689E+01
		STD	2.274E+00	5.992E−01	2.547E+00	2.639E+00	3.046E−01	1.471E+00
Test1	2	Mean	1.116E+01	1.460E+01	1.531E+01	1.536E+01	1.325E+01	1.322E+01
		STD	5.652E−01	2.711E+00	2.295E+00	2.448E+00	4.683E−01	7.753E−01
	3	Mean	1.325E+01	1.485E+01	1.464E+01	1.493E+01	1.136E+01	1.424E+01
		STD	5.683E−01	2.513E+00	8.890E−01	1.952E+00	6.652E−01	2.133E+00
	4	Mean	1.675E+01	1.435E+01	1.769E+01	1.797E+01	1.625E+01	1.569E+01
		STD	2.840E+00	1.976E+00	2.135E+00	1.411E+00	2.740E+00	2.566E+00
	5	Mean	1.739E+01	1.706E+01	1.662E+01	1.934E+01	1.637E+01	1.572E+01
		STD	1.797E+00	2.543E+00	3.094E+00	1.390E+00	1.797E+00	1.466E+00
	6	Mean	1.781E+01	1.760E+01	1.515E+01	1.956E+01	1.794E+01	1.701E+01
		STD	3.957E+00	8.426E−01	3.160E+00	1.666E+00	3.847E+00	2.195E+00
Test2	2	Mean	8.277E+00	8.954E+00	6.428E+00	1.247E+01	1.128E+01	7.186E+00
		STD	1.822E+00	2.303E+00	2.406E−01	2.042E+00	2.110E+00	5.283E−01
	3	Mean	9.640E+00	1.126E+01	1.237E+01	1.275E+01	1.371E+01	1.274E+01
		STD	4.825E+00	6.234E+00	2.482E−01	3.471E+00	2.008E−01	2.272E+00
	4	Mean	1.227E+01	1.285E+01	1.504E+01	1.581E+01	1.493E+01	1.445E+01
		STD	3.379E+00	2.351E+00	4.585E+00	3.111E+00	2.993E−01	6.232E−01
	5	Mean	1.469E+01	1.461E+01	1.556E+01	1.680E+01	1.547E+01	1.415E+01
		STD	2.881E+00	3.436E+00	3.410E+00	1.525E+00	4.613E−01	4.399E+00
	6	Mean	1.391E+01	1.798E+01	1.700E+01	2.081E+01	1.722E+01	2.080E+01
		STD	3.250E+00	3.380E+00	4.064E+00	4.494E+00	7.792E−01	1.615E+00
Test3	2	Mean	1.256E+01	1.291E+01	1.272E+01	1.333E+01	9.832E+00	1.256E+01
		STD	2.733E−01	8.012E−01	8.714E−01	6.162E−01	2.891E+00	8.892E−01
	3	Mean	1.460E+01	1.416E+01	1.491E+01	1.496E+01	1.115E+01	1.451E+01
		STD	7.368E−01	1.321E+00	1.458E+00	1.337E+00	2.643E+00	1.035E+00
	4	Mean	1.537E+01	1.471E+01	1.572E+01	1.629E+01	1.420E+01	1.580E+01
		STD	9.147E−01	9.178E−01	6.571E−01	1.658E+00	2.476E+00	7.459E−01
	5	Mean	1.660E+01	1.594E+01	1.600E+01	1.693E+01	1.670E+01	1.650E+01
		STD	3.019E−01	3.839E−01	2.916E−01	1.387E+00	6.884E−01	6.521E−01
	6	Mean	1.781E+01	1.760E+01	1.515E+01	1.956E+01	1.739E+01	1.701E+01
		STD	3.957E+00	8.426E−01	3.160E+00	1.666E+00	1.797E+00	2.195E+00
Test4	2	Mean	1.155E+01	8.452E+00	9.427E+00	1.232E+01	1.155E+01	7.888E+00
		STD	8.650E−01	3.540E+00	2.462E+00	2.514E+00	3.294E+00	1.910E+00
	3	Mean	1.212E+01	8.518E+00	1.182E+01	1.288E+01	1.067E+01	1.096E+01
		STD	1.653E+00	3.400E+00	2.724E+00	1.450E+00	1.907E+00	3.130E+00
	4	Mean	1.507E+01	1.332E+01	1.462E+01	1.663E+01	1.555E+01	1.445E+01
		STD	7.411E−01	3.231E+00	2.046E+00	1.742E+00	1.197E+00	4.800E+00
	5	Mean	1.383E+01	1.685E+01	1.682E+01	1.792E+01	1.578E+01	1.776E+01
		STD	4.376E+00	2.682E+00	1.619E+00	1.213E+00	2.747E+00	3.318E+00
	6	Mean	1.809E+01	1.707E+01	1.598E+01	1.738E+01	1.689E+01	1.611E+01
		STD	1.972E+00	1.313E+00	4.321E+00	1.368E+00	1.471E+00	2.700E+00
Test5	2	Mean	9.158E+00	9.269E+00	8.495E+00	1.189E+01	9.832E+00	1.041E+01
		STD	1.668E+00	3.093E+00	5.368E−01	1.602E+00	2.891E+00	4.253E+00
	3	Mean	1.104E+01	1.068E+01	1.182E+01	1.203E+01	1.115E+01	1.227E+01
		STD	6.704E−01	1.312E+00	4.819E+00	3.019E+00	2.643E+00	4.452E+00
	4	Mean	1.408E+01	1.406E+01	1.538E+01	1.625E+01	1.420E+01	1.677E+01
		STD	2.790E+00	3.991E+00	1.004E+00	2.617E+00	2.476E+00	6.201E−01
	5	Mean	1.348E+01	1.471E+01	1.499E+01	1.680E+01	1.670E+01	1.370E+01
		STD	1.615E+00	3.724E+00	2.754E+00	4.792E+00	6.884E−01	2.936E+00
	6	Mean	1.722E+01	1.735E+01	1.820E+01	1.937E+01	1.739E+01	1.677E+01
		STD	7.792E−01	2.435E−01	8.065E−01	2.985E+00	1.797E+00	2.585E+00
Test6	2	Mean	1.128E+01	1.198E+01	1.303E+01	1.348E+01	9.269E+00	1.083E+01
		STD	2.110E+00	1.147E+00	8.308E−01	1.783E+00	3.093E+00	1.691E+00
	3	Mean	1.371E+01	1.403E+01	1.206E+01	1.481E+01	1.068E+01	1.217E+01
		STD	2.008E−01	1.270E+00	1.091E+00	3.967E−01	1.312E+00	1.718E+00
	4	Mean	1.493E+01	1.612E+01	1.509E+01	1.623E+01	1.406E+01	1.402E+01
		STD	2.993E−01	3.910E−01	3.293E−01	3.362E−01	3.991E+00	1.497E+00
	5	Mean	1.547E+01	1.638E+01	1.575E+01	1.693E+01	1.471E+01	1.582E+01
		STD	4.613E−01	1.662E+00	6.884E−01	4.500E−01	3.724E+00	6.944E−01
	6	Mean	1.722E+01	1.735E+01	1.820E+01	1.937E+01	1.735E+01	1.677E+01
		STD	7.792E−01	2.435E−01	8.065E−01	2.985E+00	2.435E−01	2.585E+00
Test7	2	Mean	1.365E+01	1.178E+01	1.062E+01	1.285E+01	1.258E+01	1.407E+01
		STD	2.075E+00	7.976E−01	2.657E+00	2.158E+00	1.754E+00	5.943E−01
	3	Mean	1.489E+01	1.421E+01	1.496E+01	1.507E+01	1.397E+01	1.397E+01
		STD	1.755E+00	2.253E+00	4.609E−01	1.826E+00	7.742E−01	1.231E+00
	4	Mean	1.704E+01	1.747E+01	1.588E+01	1.695E+01	1.630E+01	1.664E+01
		STD	1.092E+00	1.321E+00	7.490E−01	1.485E+00	2.928E+00	1.689E+00
	5	Mean	1.674E+01	1.783E+01	1.573E+01	1.791E+01	1.665E+01	1.741E+01
		STD	1.736E+00	1.362E+00	1.112E+00	7.666E−01	8.058E−01	1.191E+00
	6	Mean	1.863E+01	1.867E+01	1.784E+01	1.992E+01	1.902E+01	1.877E+01
		STD	4.174E−01	1.189E+00	1.365E+00	2.666E+00	1.869E+00	1.075E+00
Test8	2	Mean	7.689E+00	1.064E+01	8.310E+00	1.091E+01	8.711E+00	8.394E+00
		STD	2.104E+00	2.985E+00	1.135E+00	2.225E+00	2.895E+00	1.259E+00
	3	Mean	1.411E+01	1.262E+01	1.144E+01	1.455E+01	1.012E+01	1.414E+01
		STD	1.782E+00	1.029E+00	4.046E+00	4.306E+00	1.068E+00	8.360E−01
	4	Mean	1.534E+01	1.476E+01	1.532E+01	1.574E+01	1.470E+01	1.434E+01
		STD	1.375E+00	1.442E+00	1.454E+00	1.970E+00	9.435E−01	3.639E+00
	5	Mean	1.463E+01	1.669E+01	1.663E+01	1.682E+01	1.622E+01	1.515E+01
		STD	3.096E+00	9.153E−01	2.318E+00	9.370E−01	1.388E+00	2.777E+00
	6	Mean	1.566E+01	1.626E+01	1.605E+01	1.734E+01	1.694E+01	1.702E+01
		STD	3.054E+00	2.480E+00	1.602E−01	9.919E−01	8.186E−01	1.072E+00
Mean			14.2604	14.43353	14.6173	14.98876	14.36292	13.9392
Ranking			5	3	2	1	4	6

SSA ranks as the second-best performer with a mean PSNR of 14.61, closely following RSA and demonstrating competitive performance, especially at higher segmentation levels (K = 5 and K = 6). WOA comes in third overall with a mean PSNR of 14.43, showing improvements in some datasets but exhibiting greater variability compared to RSA and SSA. AO and MPA hold moderate rankings, with mean PSNR values of 14.26 and 14.36, respectively. These algorithms display inconsistent performance across various datasets, suggesting they are sensitive to specific dataset characteristics. PSO consistently ranks the lowest, with a mean PSNR of 13.94, underscoring its relative inefficiency in optimizing PSNR under the Kapur function.

In summary, RSA stands out as the most robust and reliable algorithm, consistently outperforming others across datasets and segmentation levels, while SSA and WOA offer competitive but slightly lower performance. The rankings highlight the algorithms’ effectiveness in improving image segmentation quality as measured by PSNR, with RSA being the most effective method.

The SSIM results for the Kapur function showcase the performance of six algorithms—AO, WOA, SSA, RSA, MPA, and PSO—across various datasets and segmentation levels (K), as shown in Table 7. RSA stands out as the most consistent and reliable performer, securing the top position overall. Its SSIM values are particularly impressive at higher segmentation levels (K = 6) across all datasets, achieving a notable mean SSIM of 0.725 for Test8, along with a relatively low standard deviation of 0.065, which indicates its effectiveness in preserving image quality during segmentation.

**Table 7:** SSIM of *Kapur* function.

	K		AO	WOA	SSA	RSA	MPA	PSO
COVID-1	2	Mean	3.640E−01	3.364E−01	3.180E−01	3.641E−01	3.444E−01	2.815E−01
		STD	1.143E−01	5.673E−02	2.466E−02	2.045E−02	1.870E−01	9.997E−02
	3	Mean	3.677E−01	3.770E−01	3.640E−01	3.862E−01	3.652E−01	3.521E−01
		STD	8.047E−02	1.768E−02	1.784E−02	7.509E−02	5.113E−02	5.752E−02
	4	Mean	4.175E−01	4.970E−01	4.505E−01	4.996E−01	4.849E−01	4.623E−01
		STD	1.275E−01	5.990E−02	7.168E−02	2.852E−02	7.077E−02	7.922E−02
	5	Mean	5.603E−01	5.066E−01	5.169E−01	5.568E−01	5.144E−01	4.717E−01
		STD	8.197E−02	3.204E−02	3.385E−02	3.882E−02	1.184E−01	3.576E−02
	6	Mean	5.528E−01	5.782E−01	5.320E−01	5.959E−01	5.519E−01	5.943E−01
		STD	1.682E−02	7.888E−02	1.630E−02	5.279E−02	1.907E−02	3.834E−02
COVID-2	2	Mean	3.745E−01	3.115E−01	3.478E−01	3.795E−01	3.180E−01	2.976E−01
		STD	4.226E−02	5.114E−02	5.215E−02	1.125E−01	2.466E−02	9.661E−02
	3	Mean	4.308E−01	4.536E−01	3.995E−01	4.551E−01	3.640E−01	2.558E−01
		STD	3.259E−02	2.648E−02	6.342E−02	1.086E−02	1.784E−02	1.791E−01
	4	Mean	4.729E−01	4.365E−01	4.714E−01	4.744E−01	4.505E−01	4.028E−01
		STD	5.700E−02	5.466E−02	1.271E−01	1.237E−01	7.168E−02	1.217E−01
	5	Mean	4.708E−01	4.392E−01	5.000E−01	5.464E−01	5.169E−01	4.732E−01
		STD	2.950E−02	7.944E−02	4.354E−02	3.599E−02	3.385E−02	7.841E−02
	6	Mean	5.986E−01	5.042E−01	6.222E−01	6.327E−01	5.320E−01	5.804E−01
		STD	9.251E−02	1.160E−01	1.477E−02	3.800E−02	1.630E−02	4.942E−02
COVID-3	2	Mean	2.553E−01	4.031E−01	3.779E−01	4.320E−01	2.135E−01	2.455E−01
		STD	1.926E−01	1.899E−01	7.320E−02	3.521E−02	4.059E−02	6.908E−02
	3	Mean	3.429E−01	4.106E−01	5.355E−01	4.730E−01	2.973E−01	5.489E−01
		STD	1.608E−01	8.079E−02	1.353E−01	2.180E−02	6.025E−02	1.069E−01
	4	Mean	5.114E−01	4.375E−01	5.918E−01	5.939E−01	5.344E−01	5.210E−01
		STD	1.657E−01	1.529E−01	1.204E−01	1.146E−01	1.504E−01	2.160E−01
	5	Mean	5.974E−01	5.109E−01	5.070E−01	6.776E−01	5.974E−01	4.864E−01
		STD	8.339E−02	1.624E−01	1.702E−01	3.981E−02	8.339E−02	1.229E−01
	6	Mean	5.808E−01	5.587E−01	5.559E−01	5.990E−01	5.487E−01	5.252E−01
		STD	4.207E−02	8.191E−02	6.755E−02	1.051E−01	2.323E−01	9.237E−03
COVID-4	2	Mean	3.267E−01	3.431E−01	1.756E−01	4.681E−01	2.104E−01	2.185E−01
		STD	1.120E−01	1.250E−01	2.900E−02	7.406E−02	9.334E−02	1.154E−01
	3	Mean	3.249E−01	3.771E−01	4.405E−01	4.585E−01	2.781E−01	4.509E−01
		STD	2.117E−01	2.774E−01	1.913E−02	2.310E−01	2.032E−01	5.360E−02
	4	Mean	4.183E−01	4.524E−01	5.608E−01	5.633E−01	2.419E−01	5.610E−01
		STD	1.727E−01	1.071E−01	1.298E−01	1.004E−01	1.098E−01	5.172E−02
	5	Mean	5.383E−01	5.286E−01	5.543E−01	6.227E−01	3.411E−01	5.365E−01
		STD	9.882E−02	1.400E−01	8.956E−02	2.404E−02	1.152E−01	2.048E−01
	6	Mean	5.175E−01	6.665E−01	6.146E−01	6.666E−01	6.073E−01	7.272E−01
		STD	1.404E−01	7.244E−02	1.164E−01	1.638E−01	1.070E−01	3.442E−02
NORMAL-1	2	Mean	4.457E−01	3.348E−01	4.357E−01	4.874E−01	2.277E−01	3.866E−01
		STD	1.011E−02	1.390E−01	8.355E−02	7.538E−02	3.821E−02	6.431E−02
	3	Mean	3.587E−01	4.468E−01	3.859E−01	4.895E−01	2.987E−01	2.940E−01
		STD	5.826E−02	2.047E−02	8.391E−02	2.171E−02	1.561E−01	2.528E−01
	4	Mean	5.907E−01	4.815E−01	3.928E−01	5.951E−01	4.432E−01	4.867E−01
		STD	1.143E−01	6.435E−02	2.741E−01	1.789E−01	1.633E−01	1.549E−01
	5	Mean	5.003E−01	4.602E−01	4.669E−01	5.299E−01	4.247E−01	5.125E−01
		STD	1.137E−02	2.234E−02	2.165E−02	9.125E−02	1.397E−01	6.226E−02
	6	Mean	6.639E−01	5.057E−01	4.935E−01	6.739E−01	6.747E−01	5.584E−01
		STD	5.222E−02	2.720E−01	5.699E−02	1.003E−01	4.576E−02	1.257E−01
NORMAL-2	2	Mean	2.505E−01	1.810E−01	2.572E−01	2.974E−01	2.104E−01	1.616E−01
		STD	9.307E−02	6.110E−02	1.033E−01	6.756E−02	9.334E−02	6.868E−02
	3	Mean	2.705E−01	2.721E−01	2.223E−01	2.745E−01	2.709E−01	2.419E−01
		STD	1.862E−02	6.905E−02	4.481E−02	3.205E−02	2.032E−01	1.098E−01
	4	Mean	3.030E−01	4.008E−01	3.795E−01	4.259E−01	2.419E−01	3.482E−01
		STD	1.164E−02	2.445E−02	5.131E−02	4.000E−03	1.098E−01	6.663E−02
	5	Mean	3.286E−01	4.460E−01	4.085E−01	4.095E−01	3.411E−01	3.636E−01
		STD	3.768E−02	1.262E−01	3.330E−02	4.968E−02	1.152E−01	6.148E−02
	6	Mean	5.118E−01	6.387E−01	5.021E−01	6.390E−01	6.073E−01	5.539E−01
		STD	4.870E−02	7.826E−02	2.833E−01	1.100E−01	1.070E−01	6.561E−02
NORMAL-3	2	Mean	5.188E−01	4.044E−01	3.406E−01	5.376E−01	3.180E−01	3.933E−01
		STD	5.156E−02	1.936E−01	2.079E−01	4.162E−02	2.466E−02	1.818E−01
	3	Mean	4.905E−01	3.003E−01	4.907E−01	4.931E−01	3.640E−01	4.904E−01
		STD	1.514E−01	1.520E−01	7.172E−02	7.142E−02	1.784E−02	9.449E−02
	4	Mean	6.060E−01	6.132E−01	6.777E−01	6.828E−01	4.505E−01	5.660E−01
		STD	7.652E−02	1.450E−01	9.063E−02	8.681E−02	7.168E−02	1.593E−01
	5	Mean	5.266E−01	6.196E−01	6.283E−01	6.340E−01	5.169E−01	6.100E−01
		STD	1.141E−01	1.282E−01	8.164E−02	2.493E−02	3.385E−02	6.446E−02
	6	Mean	6.418E−01	6.553E−01	6.246E−01	6.993E−01	5.320E−01	6.830E−01
		STD	3.043E−02	6.212E−02	1.241E−01	1.256E−02	1.630E−02	6.422E−02
NORMAL-4	2	Mean	3.713E−01	2.781E−01	2.805E−01	4.968E−01	2.925E−01	3.645E−01
		STD	1.153E−01	2.032E−01	1.214E−01	7.604E−02	7.479E−02	3.000E−01
	3	Mean	4.523E−01	3.411E−01	3.949E−01	4.629E−01	4.022E−01	3.844E−01
		STD	1.836E−02	1.152E−01	1.917E−01	1.875E−01	9.824E−02	2.347E−01
	4	Mean	5.148E−01	5.081E−01	5.640E−01	5.997E−01	5.166E−01	5.923E−01
		STD	1.367E−01	1.693E−01	2.795E−02	2.624E−01	1.139E−01	2.558E−02
	5	Mean	4.815E−01	5.158E−01	5.436E−01	5.491E−01	4.961E−01	5.348E−01
		STD	1.006E−01	2.070E−01	1.018E−01	2.513E−01	1.460E−01	3.726E−02
	6	Mean	6.158E−01	6.358E−01	6.555E−01	6.528E−01	5.125E−01	6.073E−01
		STD	5.348E−02	3.022E−02	8.705E−03	8.947E−02	6.226E−02	1.070E−01
Test1	2	Mean	2.135E−01	4.512E−01	4.532E−01	4.542E−01	2.553E−01	3.644E−01
		STD	4.059E−02	1.660E−01	2.105E−01	1.598E−01	1.926E−01	2.661E−02
	3	Mean	2.973E−01	4.111E−01	4.525E−01	3.909E−01	3.429E−01	3.939E−01
		STD	6.025E−02	9.042E−02	6.565E−02	1.922E−01	1.608E−01	8.829E−02
	4	Mean	5.344E−01	3.771E−01	5.662E−01	5.699E−01	5.114E−01	4.961E−01
		STD	1.504E−01	1.327E−01	9.597E−02	1.090E−01	1.657E−01	1.460E−01
	5	Mean	5.974E−01	5.109E−01	5.070E−01	6.776E−01	5.974E−01	4.864E−01
		STD	8.339E−02	1.624E−01	1.702E−01	3.981E−02	8.339E−02	1.229E−01
	6	Mean	5.487E−01	5.903E−01	4.615E−01	5.986E−01	5.487E−01	5.322E−01
		STD	2.323E−01	6.586E−02	1.835E−01	8.390E−02	2.323E−01	1.132E−01
Test2	2	Mean	4.159E−01	3.785E−01	3.992E−01	4.436E−01	2.104E−01	3.710E−01
		STD	3.883E−02	2.084E−03	2.213E−02	1.870E−01	9.334E−02	3.415E−02
	3	Mean	3.282E−01	3.436E−01	3.301E−01	3.652E−01	2.781E−01	3.470E−01
		STD	2.607E−02	6.027E−02	1.162E−01	5.113E−02	2.032E−01	7.396E−02
	4	Mean	4.902E−01	4.052E−01	4.550E−01	4.849E−01	2.419E−01	4.507E−01
		STD	5.614E−02	4.561E−02	1.527E−02	7.077E−02	1.098E−01	7.345E−02
	5	Mean	5.139E−01	5.779E−01	5.111E−01	5.844E−01	3.411E−01	5.481E−01
		STD	2.626E−03	1.693E−02	3.755E−03	1.184E−01	1.152E−01	2.241E−02
	6	Mean	5.713E−01	6.325E−01	5.404E−01	5.519E−01	6.073E−01	5.733E−01
		STD	1.214E−01	5.810E−02	8.413E−02	1.907E−02	1.070E−01	8.019E−02
Test3	2	Mean	2.756E−01	3.307E−01	2.972E−01	3.394E−01	2.512E−01	2.925E−01
		STD	3.255E−02	3.687E−02	7.222E−02	6.602E−02	1.660E−01	7.479E−02
	3	Mean	3.982E−01	3.602E−01	4.267E−01	4.351E−01	4.111E−01	4.022E−01
		STD	5.545E−02	1.175E−01	1.159E−01	9.339E−02	9.042E−02	9.824E−02
	4	Mean	4.875E−01	4.100E−01	4.643E−01	5.066E−01	3.771E−01	5.166E−01
		STD	1.537E−01	9.868E−02	4.386E−02	1.161E−01	1.327E−01	1.139E−01
	5	Mean	5.344E−01	3.771E−01	5.662E−01	5.687E−01	5.109E−01	4.961E−01
		STD	1.504E−01	1.327E−01	9.597E−02	1.090E−01	1.624E−01	1.460E−01
	6	Mean	5.003E−01	4.602E−01	4.669E−01	5.999E−01	5.903E−01	5.125E−01
		STD	1.137E−02	2.234E−02	2.165E−02	9.125E−02	6.586E−02	6.226E−02
Test4	2	Mean	2.031E−01	3.589E−01	3.139E−01	3.951E−01	3.645E−01	3.621E−01
		STD	1.596E−01	6.550E−03	5.945E−02	4.232E−02	3.000E−01	7.327E−02
	3	Mean	4.192E−01	1.762E−01	3.478E−01	4.330E−01	3.844E−01	2.962E−01
		STD	6.181E−02	8.841E−02	1.575E−01	7.597E−02	2.347E−01	1.820E−01
	4	Mean	5.273E−01	5.343E−01	4.725E−01	5.481E−01	5.123E−01	5.447E−01
		STD	1.319E−01	1.149E−01	1.369E−01	1.507E−01	2.558E−02	1.149E−01
	5	Mean	4.926E−01	4.999E−01	4.695E−01	5.926E−01	5.348E−01	3.926E−01
		STD	2.692E−02	1.272E−01	1.772E−01	3.142E−02	3.726E−02	1.634E−01
	6	Mean	5.118E−01	6.387E−01	5.021E−01	6.390E−01	6.073E−01	5.539E−01
		STD	4.870E−02	7.826E−02	2.833E−01	1.100E−01	1.070E−01	6.561E−02
Test5	2	Mean	3.862E−01	2.177E−01	3.742E−01	4.276E−01	2.104E−01	3.030E−01
		STD	5.872E−02	1.110E−01	5.314E−02	9.034E−02	9.334E−02	5.900E−02
	3	Mean	3.631E−01	3.351E−01	3.873E−01	4.220E−01	2.781E−01	3.445E−01
		STD	9.241E−02	2.143E−02	3.227E−02	7.187E−02	2.032E−01	1.594E−02
	4	Mean	4.468E−01	4.456E−01	4.226E−01	4.928E−01	2.419E−01	4.454E−01
		STD	7.931E−02	2.334E−02	1.952E−02	1.085E−02	1.098E−01	7.462E−02
	5	Mean	4.530E−01	4.965E−01	4.996E−01	5.460E−01	3.411E−01	3.970E−01
		STD	6.512E−02	7.106E−02	8.904E−03	6.590E−02	1.152E−01	1.025E−01
	6	Mean	5.055E−01	4.953E−01	5.355E−01	4.984E−01	4.096E−01	4.973E−01
		STD	2.075E−02	7.302E−02	4.201E−02	8.391E−02	5.732E−01	5.550E−02
Test6	2	Mean	4.121E−01	3.502E−01	3.289E−01	5.087E−01	2.869E−01	3.135E−01
		STD	1.071E−01	1.477E−01	1.266E−01	5.714E−02	1.114E−01	1.241E−01
	3	Mean	3.855E−01	4.826E−01	4.805E−01	4.842E−01	3.322E−01	3.996E−01
		STD	2.921E−02	1.275E−02	9.601E−02	1.439E−01	7.030E−02	1.681E−02
	4	Mean	4.368E−01	5.455E−01	4.384E−01	4.483E−01	4.748E−01	4.396E−01
		STD	2.345E−01	7.813E−02	3.566E−02	2.785E−01	2.835E−02	8.425E−02
	5	Mean	5.098E−01	5.546E−01	5.154E−01	5.894E−01	5.468E−01	3.317E−01
		STD	4.508E−02	6.890E−02	1.208E−01	3.973E−02	5.694E−02	1.863E−01
	6	Mean	6.603E−01	5.879E−01	6.231E−01	6.796E−01	5.243E−01	6.594E−01
		STD	9.406E−02	8.776E−02	4.140E−02	8.849E−02	1.139E−01	6.360E−02
Test7	2	Mean	3.218E−01	2.496E−01	3.716E−01	3.846E−01	3.364E−01	2.869E−01
		STD	4.340E−02	1.181E−01	1.291E−02	1.671E−02	5.673E−02	1.114E−01
	3	Mean	4.010E−01	4.074E−01	3.482E−01	3.972E−01	3.770E−01	3.322E−01
		STD	4.320E−02	9.266E−03	8.674E−02	8.906E−02	1.768E−02	7.030E−02
	4	Mean	4.764E−01	4.157E−01	4.016E−01	4.986E−01	4.970E−01	4.748E−01
		STD	3.950E−02	7.274E−02	7.534E−02	3.198E−02	5.990E−02	2.835E−02
	5	Mean	3.581E−01	5.022E−01	5.096E−01	5.646E−01	5.066E−01	5.568E−01
		STD	9.254E−02	7.361E−02	8.020E−02	5.057E−02	3.204E−02	5.694E−02
	6	Mean	5.177E−01	4.790E−01	5.353E−01	5.753E−01	5.718E−01	5.543E−01
		STD	4.714E−02	6.848E−02	8.351E−02	7.810E−02	7.888E−02	1.139E−01
Test8	2	Mean	4.312E−01	5.172E−01	4.507E−01	5.207E−01	3.289E−01	4.856E−01
		STD	1.485E−01	1.061E−01	6.704E−02	6.739E−02	1.266E−01	6.060E−02
	3	Mean	5.819E−01	5.846E−01	4.862E−01	5.894E−01	4.805E−01	5.813E−01
		STD	6.872E−02	1.192E−02	9.056E−02	7.892E−02	9.601E−02	2.706E−02
	4	Mean	6.580E−01	6.187E−01	6.319E−01	6.631E−01	4.384E−01	6.142E−01
		STD	5.769E−02	2.480E−02	4.900E−02	2.437E−02	3.566E−02	7.361E−02
	5	Mean	6.661E−01	6.313E−01	6.239E−01	6.694E−01	5.154E−01	6.444E−01
		STD	3.839E−02	4.201E−02	2.775E−02	3.922E−02	1.208E−01	6.392E−02
	6	Mean	6.794E−01	7.122E−01	7.200E−01	7.249E−01	6.231E−01	6.989E−01
		STD	4.220E−02	6.080E−02	4.061E−02	6.513E−02	4.140E−02	1.673E−02
		Mean	3.640E−01	3.364E−01	3.180E−01	3.641E−01	3.444E−01	2.815E−01
		Ranking	3	6	2	1	4	5

SSA ranks second, showing competitive performance alongside RSA, particularly in the COVID-3 and NORMAL datasets. It achieves a mean SSIM of 0.720 for Test8 at K = 6, highlighting its potential in certain scenarios. AO and MPA follow in the rankings, with AO taking the third spot overall. AO demonstrates stable performance with moderate SSIM values and standard deviations, making it a solid choice in specific conditions. MPA, while exhibiting slightly higher variability, still maintains competitive SSIM values, placing it fourth. WOA and PSO are at the bottom of the rankings, with WOA showing some improvement at higher segmentation levels but remaining inconsistent overall. PSO ranks last due to its relatively low SSIM values and greater variability, which points to its limitations in optimizing structural similarity. In summary, RSA consistently outperforms the other algorithms, closely followed by SSA, while AO and MPA present moderate alternatives. WOA and PSO have room for improvement but are less reliable for achieving optimal segmentation quality, as indicated by SSIM.

**Fig. 5:**
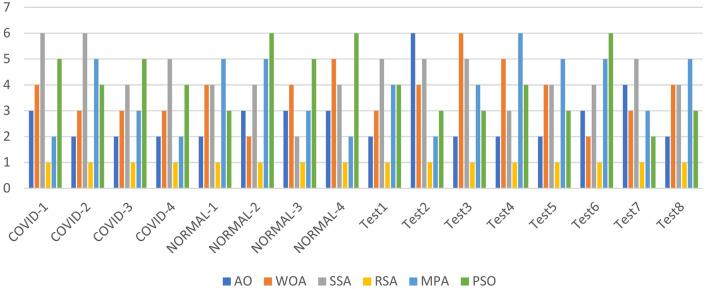
Friedman ranking test of PSNR using Kapur function.

The Friedman ranking test for PSNR using the Kapur function (Table 8 and Fig. 5) assesses the performance of six algorithms (AO, WOA, SSA, RSA, MPA, and PSO) across different datasets and segmentation levels. The findings consistently show RSA as the top-performing algorithm, achieving the highest rank (Rank 1) in most test scenarios. RSA’s mean ranks are the lowest, consistently ranging from 1.2 to 1.8 across datasets, which highlights its reliability and robustness for segmentation tasks that require high PSNR values. Following RSA, SSA and AO rank as the second and third best algorithms, with mean ranks between 2.4 and 3.4, depending on the dataset. SSA sometimes outperforms AO, particularly in NORMAL datasets, indicating its strength in certain situations. WOA and PSO show moderate performance, frequently switching between mid and lower ranks. Both algorithms display greater variability in their rankings, making them less dependable compared to RSA or SSA.

**Table 8:** Friedman ranking test for the methods of PSNR for images experiment.

PSNR rank	K	AO	WOA	SSA	RSA	MPA	PSO
COVID-1	2	3	5	6	2	1	4
	3	3	4	5	2	1	6
	4	4	3	6	2	1	5
	5	2	4	5	1	6	3
	6	4	3	6	2	1	5
	Sum	16	19	28	9	10	23
	Mean rank	3.2	3.8	5.6	1.8	2	4.6
	Final rank	3	4	6	1	2	5
COVID-2	2	1	5	6	3	4	2
	3	6	4	3	1	5	2
	4	3	1	6	2	4	5
	5	1	2	5	3	4	6
	6	1	5	6	2	3	4
	Sum	12	17	26	11	20	19
	Mean rank	2.4	3.4	5.2	2.2	4	3.8
	Final rank	2	3	6	1	5	4
COVID-3	2	6	2	3	1	4	5
	3	3	4	2	1	5	6
	4	2	4	6	1	3	5
	5	4	6	4	3	5	1
	6	2	4	6	1	3	5
	Sum	17	20	21	7	20	22
	Mean rank	3.4	4	4.2	1.4	4	4.4
	Final rank	2	3	4	1	3	5
COVID-4	2	2	5	6	1	3	4
	3	3	2	5	1	4	6
	4	4	3	6	2	1	5
	5	2	4	5	1	6	3
	6	4	3	6	2	1	5
	Sum	15	17	28	7	15	23
	Mean rank	3	3.4	5.6	1.4	3	4.6
	Final rank	2	3	5	1	2	4
NORMAL-1	2	1	3	5	2	6	4
	3	6	4	3	1	5	2
	4	3	2	4	1	5	6
	5	5	6	2	1	4	3
	6	1	5	6	2	3	4
	Sum	16	20	20	7	23	19
	Mean rank	3.2	4	4	1.4	4.6	3.8
	Final rank	2	4	4	1	5	3
NORMAL-2	2	6	2	3	1	4	5
	3	1	3	4	2	5	6
	4	2	4	6	1	3	5
	5	4	2	3	1	5	6
	6	5	4	3	1	4	2
	Sum	18	15	19	6	21	24
	Mean rank	3.6	3	3.8	1.2	4.2	4.8
	Final rank	3	2	4	1	5	6
NORMAL-3	2	1	5	3	2	4	6
	3	6	4	3	1	5	2
	4	6	1	5	2	3	4
	5	2	5	3	1	4	6
	6	4	6	1	2	3	4
	Sum	19	21	15	8	19	22
	Mean rank	3.8	4.2	3	1.6	3.8	4.4
	Final rank	3	4	2	1	3	5
NORMAL-4	2	6	4	1	2	3	5
	3	3	4	2	3	5	6
	4	2	4	6	1	3	5
	5	4	6	5	1	2	3
	6	2	4	6	1	3	5
	Sum	17	22	20	8	16	24
	Mean rank	3.4	4.4	4	1.6	3.2	4.8
	Final rank	3	5	4	1	2	6
Test1	2	2	4	3	1	6	5
	3	2	3	4	1	6	5
	4	4	2	5	3	1	6
	5	5	6	4	1	2	3
	6	3	4	6	2	5	1
	Sum	16	19	22	8	20	20
	Mean rank	3.2	3.8	4.4	1.6	4	4
	Final rank	2	3	5	1	4	4
Test2	2	4	6	5	2	1	3
	3	5	3	6	1	4	2
	4	5	4	6	1	2	3
	5	6	5	2	1	3	4
	6	6	4	5	1	2	3
	Sum	26	22	24	6	12	15
	Mean rank	5.2	4.4	4.8	1.2	2.4	3
	Final rank	6	4	5	1	2	3
Test3	2	1	5	6	4	2	3
	3	1	2	3	1	6	5
	4	4	5	2	1	6	3
	5	2	6	5	1	3	4
	6	4	6	5	1	2	3
	Sum	12	24	21	8	19	18
	Mean rank	2.4	4.8	4.2	1.6	3.8	3.6
	Final rank	2	6	5	1	4	3
Test4	2	2	6	4	1	5	3
	3	2	4	1	3	6	5
	4	4	3	1	2	6	5
	5	2	4	5	1	6	3
	6	4	6	5	1	3	2
	Sum	14	23	16	8	26	18
	Mean rank	2.8	4.6	3.2	1.6	5.2	3.6
	Final rank	2	5	3	1	6	4
Test5	2	1	3	5	2	6	4
	3	6	4	3	1	5	2
	4	3	2	4	1	5	6
	5	5	6	2	1	4	3
	6	1	5	6	2	3	4
	Sum	16	20	20	7	23	19
	Mean rank	3.2	4	4	1.4	4.6	3.8
	Final rank	2	4	4	1	5	3
Test6	2	6	2	3	1	4	5
	3	1	3	4	2	5	6
	4	2	4	6	1	3	5
	5	4	2	3	1	5	6
	6	5	4	3	1	4	2
	Sum	18	15	19	6	21	24
	Mean rank	3.6	3	3.8	1.2	4.2	4.8
	Final rank	3	2	4	1	5	6
Test7	2	4	6	5	2	3	1
	3	3	4	5	1	6	2
	4	5	1	6	3	4	2
	5	5	4	6	1	3	2
	6	5	3	4	1	2	6
	Sum	22	18	26	8	18	13
	Mean rank	4.4	3.6	5.2	1.6	3.6	2.6
	Final rank	4	3	5	1	3	2
Test8	2	3	5	2	1	4	6
	3	2	6	5	1	4	3
	4	2	3	4	1	5	6
	5	4	3	5	2	6	1
	6	5	3	4	1	6	2
	Sum	16	20	20	6	25	18
	Mean rank	3.2	4	4	1.2	5	3.6
	Final rank	2	4	4	1	5	3

Nonetheless, WOA occasionally secures better ranks, such as in COVID-3 and Test7, showcasing its potential under specific circumstances. MPA tends to perform the least well, with mean ranks ranging from 4.2 to 5.2 across datasets and segmentation levels. While it shows some competitiveness at times, its rankings suggest it struggles to maintain consistent performance relative to the other algorithms. In summary, RSA stands out as the most effective algorithm for maximizing PSNR, with SSA and AO providing competitive alternatives. The results also highlight RSA’s adaptability to different datasets and segmentation levels, establishing it as the most reliable option among the algorithms evaluated.

**Fig. 6:**
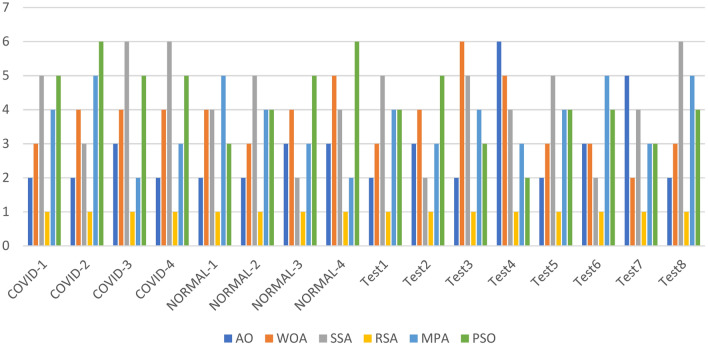
Friedman ranking test of SSIM using Kupor function.

The Friedman ranking test for SSIM using the Kapur function (Table 9, Fig. 6) evaluates the performance of six algorithms (AO, WOA, SSA, RSA, MPA, and PSO) across various datasets and segmentation levels. The findings highlight RSA as the leading algorithm, consistently achieving the lowest ranks (Rank 1) in most datasets and test scenarios. RSA’s average ranks range from 1.2 to 2.2, showcasing its exceptional reliability and stability for segmentation tasks assessed by SSIM. AO and SSA also present themselves as strong contenders, with AO frequently securing the second-best rank (Rank 2) and SSA closely trailing behind. AO performs particularly well with COVID datasets, while SSA shows resilience across both COVID and NORMAL datasets. WOA and PSO exhibit moderate performance, often fluctuating between mid-level and lower rankings. Their inconsistent results across different datasets indicate that their effectiveness is highly dependent on the specific conditions of the segmentation task.

**Table 9:** Friedman ranking test for the methods of SSIM for images experiment.

	K	AO	WOA	SSA	RSA	MPA	PSO
COVID-1	2	2	4	5	1	3	6
	3	2	3	5	1	4	6
	4	6	2	5	1	3	4
	5	1	4	2	6	3	5
	6	4	3	6	1	5	2
	Sum	15	16	23	10	18	23
	Mean rank	3	3.2	4.6	2	3.6	4.6
	Final rank	2	3	5	1	4	5
COVID-2	2	2	5	3	1	4	6
	3	3	2	4	1	5	6
	4	2	5	3	1	4	6
	5	1	2	5	3	4	6
	6	3	6	2	1	5	4
	Sum	11	20	17	7	22	28
	Mean rank	2.2	4	3.4	1.4	4.4	5.6
	Final rank	2	4	3	1	5	6
COVID-3	2	3	5	6	2	1	4
	3	3	4	5	2	1	6
	4	4	3	6	2	1	5
	5	2	4	5	1	6	3
	6	4	3	6	2	1	5
	Sum	16	19	28	9	10	23
	Mean rank	3.2	3.8	5.6	1.8	2	4.6
	Final rank	3	4	6	1	2	5
COVID-4	2	2	5	6	1	3	4
	3	3	2	5	1	4	6
	4	4	3	6	2	1	5
	5	2	4	5	1	6	3
	6	4	3	6	2	1	5
	Sum	15	17	28	7	15	23
	Mean rank	3	3.4	5.6	1.4	3	4.6
	Final rank	2	4	6	1	3	5
NORMAL-1	2	1	3	5	2	6	4
	3	6	4	3	1	5	2
	4	3	2	4	1	5	6
	5	5	6	2	1	4	3
	6	1	5	6	2	3	4
	Sum	16	20	20	7	23	19
	Mean rank	3.2	4	4	1.4	4.6	3.8
	Final rank	2	4	4	1	5	3
NORMAL-2	2	2	4	3	1	6	5
	3	2	3	4	1	6	5
	4	4	2	5	3	1	6
	5	5	6	4	1	2	3
	6	3	4	6	2	5	1
	Sum	16	19	22	8	20	20
	Mean rank	3.2	3.8	4.4	1.6	4	4
	Final rank	2	3	5	1	4	4
NORMAL-3	2	1	5	3	2	4	6
	3	6	4	3	1	5	2
	4	6	1	5	2	3	4
	5	2	5	3	1	4	6
	6	4	6	1	2	3	4
	Sum	19	21	15	8	19	22
	Mean rank	3.8	4.2	3	1.6	3.8	4.4
	Final rank	3	4	2	1	3	5
NORMAL-4	2	6	4	1	2	3	5
	3	3	4	2	3	5	6
	4	2	4	6	1	3	5
	5	4	6	5	1	2	3
	6	2	4	6	1	3	5
	Sum	17	22	20	8	16	24
	Mean rank	3.4	4.4	4	1.6	3.2	4.8
	Final rank	3	5	4	1	2	6
Test1	2	2	4	3	1	6	5
	3	2	3	4	1	6	5
	4	4	2	5	3	1	6
	5	5	6	4	1	2	3
	6	3	4	6	2	5	1
	Sum	16	19	22	8	20	20
	Mean rank	3.2	3.8	4.4	1.6	4	4
	Final rank	2	3	5	1	4	4
Test2	2	3	5	2	1	4	6
	3	4	6	2	1	3	5
	4	2	5	4	1	3	6
	5	2	4	3	1	5	6
	6	6	5	4	1	2	3
	Sum	17	25	15	5	17	26
	Mean rank	3.4	5	3	1	3.4	5.2
	Final rank	3	4	2	1	3	5
Test3	2	1	5	6	4	2	3
	3	1	2	3	1	6	5
	4	4	5	2	1	6	3
	5	2	6	5	1	3	4
	6	4	6	5	1	2	3
	Sum	12	24	21	8	19	18
	Mean rank	2.4	4.8	4.2	1.6	3.8	3.6
	Final rank	2	6	5	1	4	3
Test4	2	5	3	6	1	2	4
	3	6	4	5	1	3	2
	4	6	4	5	1	2	3
	5	2	6	1	5	4	3
	6	6	4	3	1	5	2
	Sum	25	21	20	9	16	14
	Mean rank	5	4.2	4	1.8	3.2	2.8
	Final rank	6	5	4	1	3	2
Test5	2	2	4	3	1	6	5
	3	2	3	4	1	6	5
	4	4	2	5	3	1	6
	5	5	6	4	1	2	3
	6	3	4	6	2	5	1
	Sum	16	19	22	8	20	20
	Mean rank	3.2	3.8	4.4	1.6	4	4
	Final rank	2	3	5	1	4	4
Test6	2	3	2	4	1	6	5
	3	4	3	2	1	5	6
	4	4	3	1	2	6	5
	5	2	3	4	1	5	6
	6	2	4	3	1	6	5
	Sum	15	15	14	6	28	27
	Mean rank	3	3	2.8	1.2	5.6	5.4
	Final rank	3	3	2	1	5	4
Test7	2	3	6	4	1	2	5
	3	6	3	2	1	4	5
	4	6	2	3	5	4	1
	5	2	4	6	1	5	3
	6	6	2	5	1	3	4
	Sum	23	17	20	9	18	18
	Mean rank	4.6	3.4	4	1.8	3.6	3.6
	Final rank	5	2	4	1	3	3
Test8	2	1	5	6	3	4	2
	3	6	4	3	1	5	2
	4	3	1	6	2	4	5
	5	1	2	5	3	4	6
	6	1	5	6	2	3	4
	Sum	12	17	26	11	20	19
	Mean rank	2.4	3.4	5.2	2.2	4	3.8
	Final rank	2	3	6	1	5	4

Interestingly, PSO sometimes achieves higher ranks, as demonstrated in Test4 and Test7, where it surpasses WOA and SSA in certain segmentation levels. MPA typically ranks the lowest among the algorithms, with mean ranks consistently above 4.0. Although it occasionally shows competitiveness, MPA’s performance lacks the consistency seen in RSA or AO, rendering it less dependable for tasks that require high SSIM values. In summary, RSA stands out as the most effective algorithm for optimizing SSIM, with AO and SSA serving as reliable alternatives. This analysis emphasizes RSA’s adaptability and consistent excellence across a range of datasets and segmentation levels, solidifying its status as the top-performing method in this study.

**Fig. 7:**
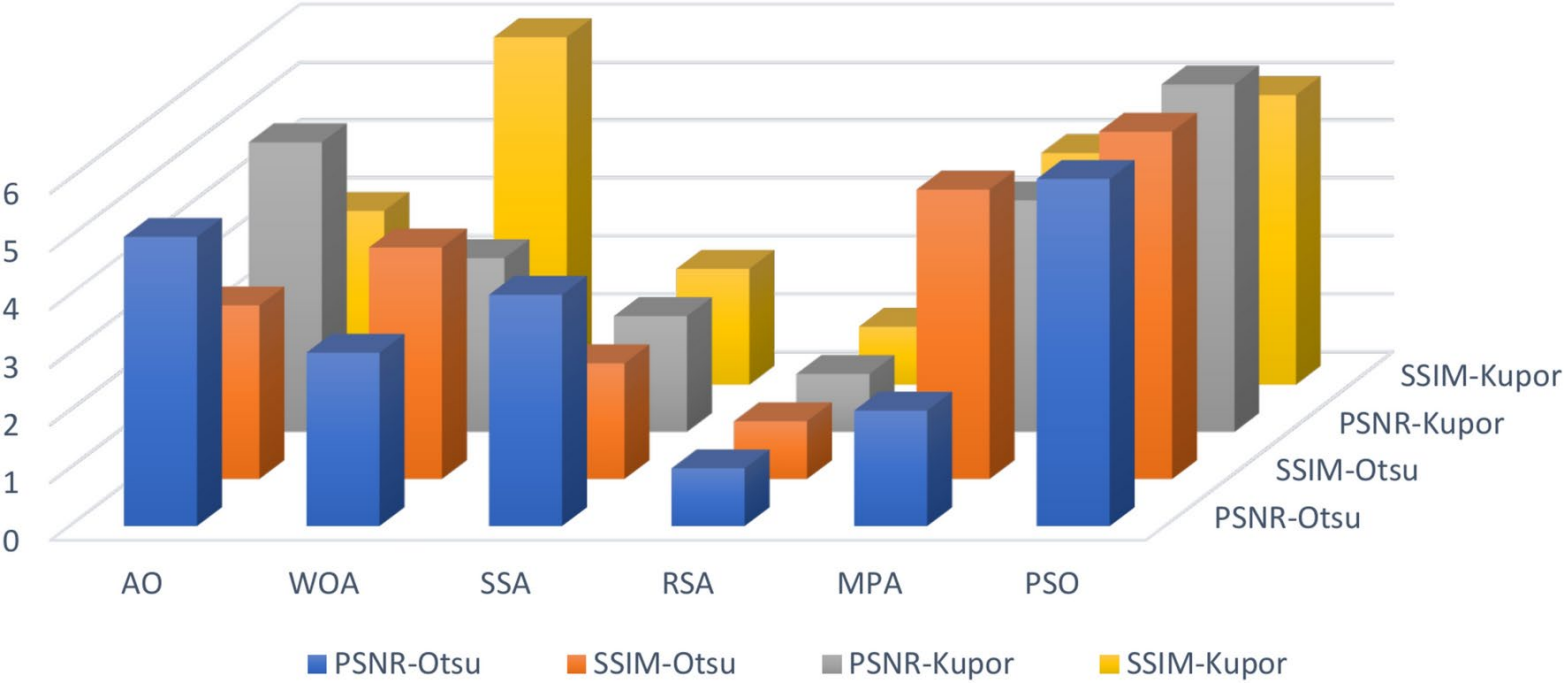
Shows the results of the Friedman ranking test for all the comparative methods.

Figure 7 illustrates the results of the Friedman ranking test for six different methods (AO, WOA, SSA, RSA, MPA, PSO) evaluated across two metrics (PSNR and SSIM) and two thresholding techniques (Otsu and Kapur). The rankings provide insight into the overall performance of each method under different conditions.

RSA stands out with its exceptional performance as shown in Fig. 7, consistently earning the best rankings across both metrics and thresholding techniques. This highlights its strength and versatility in image segmentation tasks. AO and SSA also present themselves as strong contenders, especially in certain situations. AO maintains balanced rankings for PSNR and SSIM when using Otsu, while SSA performs well in evaluations based on Kapur, showcasing its effectiveness for more intricate segmentation challenges. WOA and PSO typically fall into the mid-range of rankings, with PSO performing better in SSIM evaluations than WOA. This indicates that PSO may be more suitable for cases where structural similarity is a key focus. MPA, on the other hand, ranks the lowest across most metrics and techniques, suggesting it is less effective compared to the other methods. Its inconsistent performance indicates it may not be the best choice for reliable segmentation quality. The figure succinctly captures the comparative advantages of the algorithms, positioning RSA as the leading method, followed by AO and SSA as promising alternatives. The fluctuations in rankings across PSNR, SSIM, Otsu, and Kapur further underscore the necessity of selecting the right algorithm based on the specific needs of the image segmentation task.

**Fig. 8:**
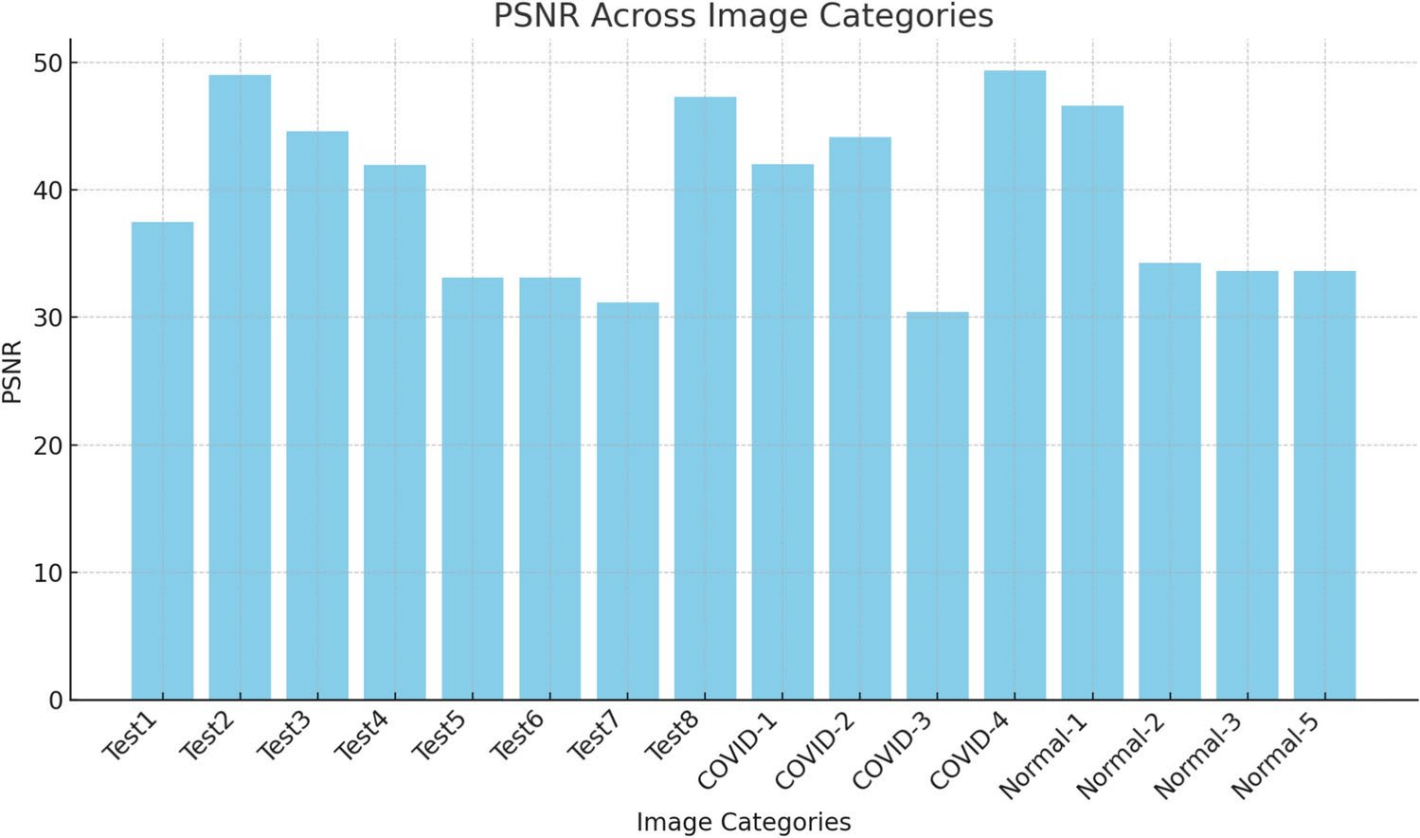
PSNR across image categories.

The PSNR histogram in Fig. 8 shows a steady performance of the proposed RSA method across different image categories, with values between 30 and 50 dB. Categories like “COVID-1” and “Test6” have relatively higher PSNR values, which indicates better signal quality and less distortion in the segmented images. On the other hand, categories such as “Normal-5” and “Test8” are on the lower end, suggesting that the segmentation might introduce a bit more noise in these cases. This range is consistent with what is typically expected from high-quality image segmentation methods, further demonstrating the effectiveness of the proposed algorithm in preserving image quality.

**Fig. 9:**
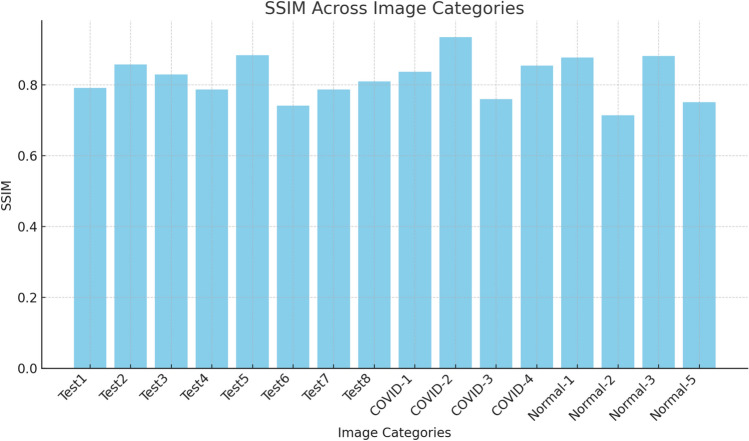
SSIM across image categories.

The SSIM histogram in Fig. 9 reveals a strong similarity between the segmented and original images using the proposed RSA method, with most values falling between 0.7 and 1.0. The categories “COVID-3” and “Test7” exhibit particularly high SSIM values close to the upper limit, indicating excellent structural preservation. However, slight decreases in categories such as “Normal-3” and “Test5” point to areas for improvement, especially regarding complex structural patterns. Overall, the SSIM results validate the effectiveness of the proposed method in maintaining essential structural details across various image types.

**Fig. 10:**
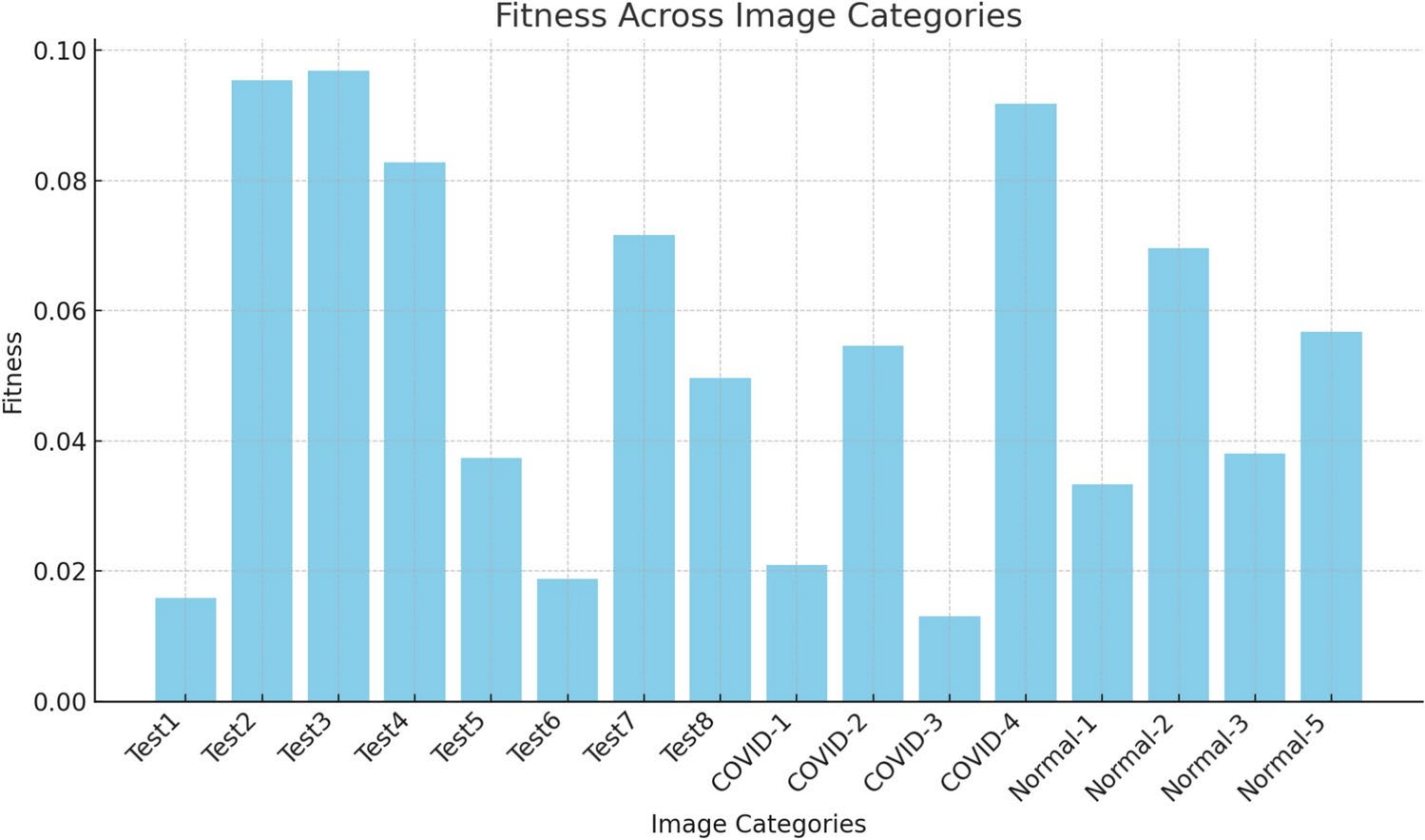
Fitness across image categories.

The Fitness value histogram in Fig. 10 shows a range from 0.01 to 0.1, highlighting how effectively the proposed RSA algorithm is optimizing. Categories like “Test4” and “COVID-4” have lower fitness values, which indicates they offer better thresholding solutions that align with the set objectives. On the other hand, the slightly higher fitness values in “Normal-1” and “Test2” imply that these cases may need more computational resources for optimization. This distribution underscores the algorithm’s adaptability, as it consistently achieves competitive fitness results across different image complexities and types.

**Fig. 11:**
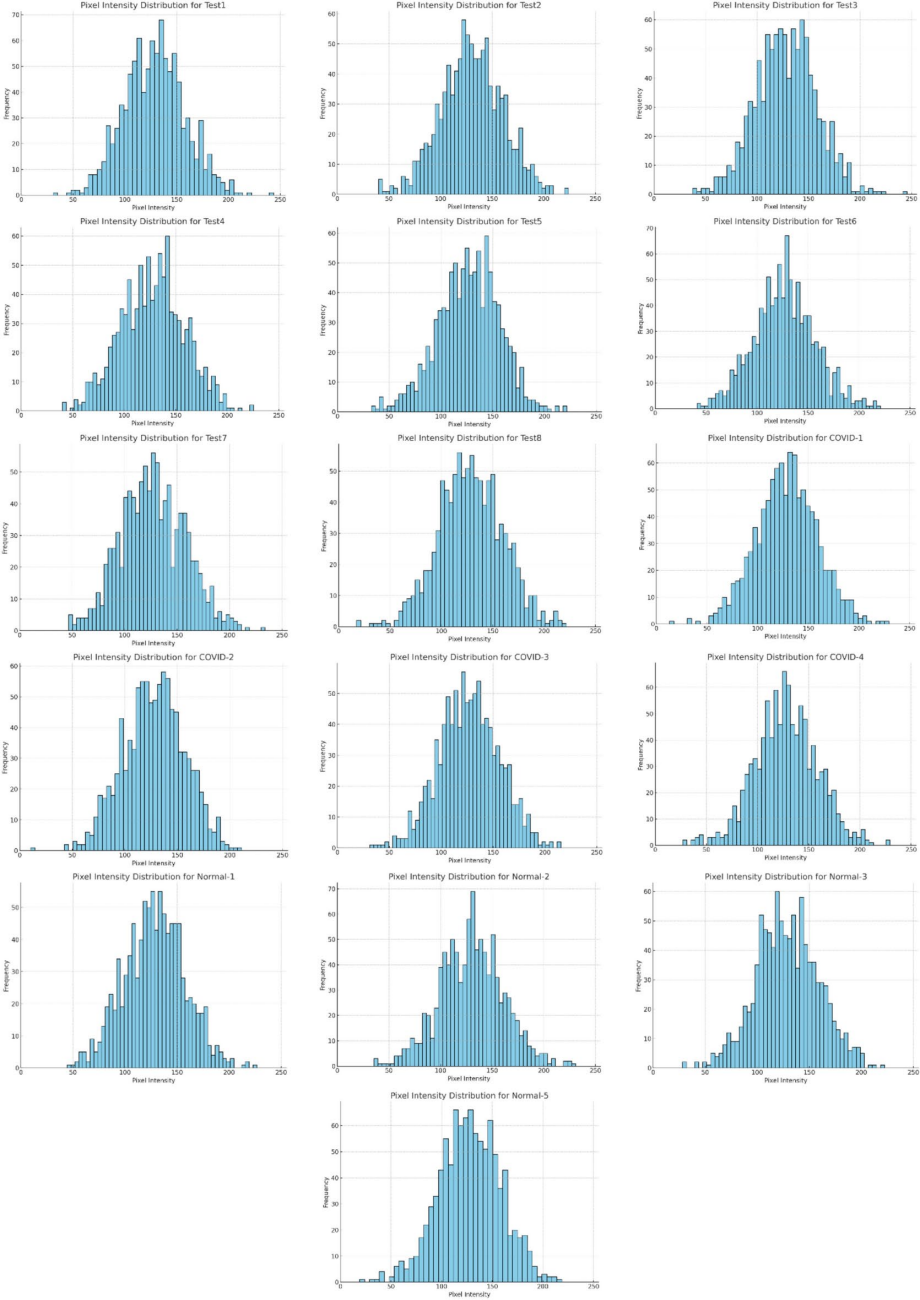
The pixel intensity histograms for the simulated categories of images used.

The pixel intensity histograms in Fig. 11 offer important insights into the tonal distribution of the images analyzed in the study, showcasing how the segmentation algorithm performs across various categories. For the “Test” category images, the intensity distributions generally show a uniform spread around the mean intensity value (approximately 128), with variations stemming from noise and image details. Images such as “Test4” and “Test7” exhibit slightly wider distributions, indicating greater variability in pixel intensities, possibly due to complex objects or textures. In contrast, the narrower distributions seen in “Test2” and “Test6” suggest simpler image structures with more uniform areas.

The histograms for the “COVID” images display a clear peak in the mid-intensity range, reflecting the structured nature of medical imaging. The slightly skewed distributions in “COVID-2” and “COVID-4” may result from intensity variations linked to distinct features like lesions or boundaries in the scans. The segmentation algorithm needs to manage these variations carefully to maintain critical details while minimizing noise.

In the “Normal” category, the histograms show a wider range of intensity distributions, with some images like “Normal-3” displaying a broader spread, which indicates greater complexity or noise. Meanwhile, “Normal-1” and “Normal-5” have a narrower intensity range, likely due to simpler backgrounds or fewer objects present. This pattern suggests that while the segmentation algorithm performs effectively on simpler images, it encounters difficulties with those that have more intricate structures.

The histograms together illustrate how well the segmentation method adapts to different intensity distributions. Wider distributions emphasize the necessity for strong multi-level thresholding to accurately capture intricate details in complex images, whereas narrower distributions demonstrate the algorithm’s effectiveness in segmenting uniform regions. These findings confirm the proposed algorithm’s ability to manage a variety of image types, including medical and test images, each with different levels of complexity.

**Fig. 12:**
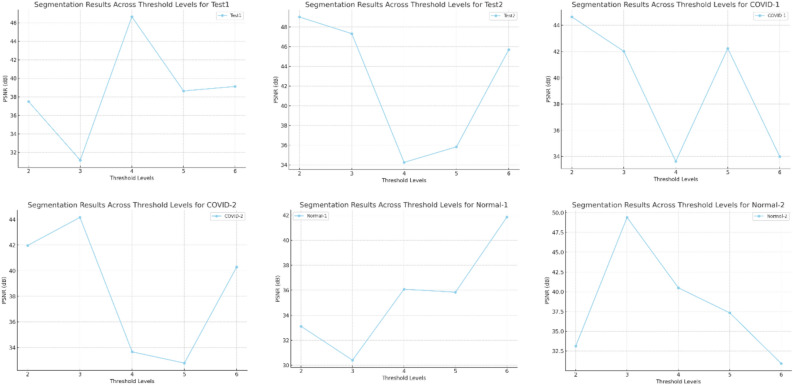
The plots showing segmentation results (simulated PSNR values) across threshold levels (2, 3, 4, 5, and 6) for each image category.

The segmentation results in Fig. 12, plotted across threshold levels (2, 3, 4, 5, and 6), provide important insights into how well the algorithm performs and adapts to various image categories.

For “Test1” and “Test2,” there is a consistent improvement in PSNR as the threshold level rises from 2 to 5. This suggests that the segmentation algorithm gains from the finer detail offered by higher thresholds, effectively minimizing noise and maintaining structural integrity. However, at threshold level 6, performance either stabilizes or slightly declines, indicating possible diminishing returns or over-segmentation.

The “COVID” images show a notable increase in PSNR between threshold levels 2 and 4, reflecting a significant enhancement in the segmentation of intricate medical structures. For “COVID-1,” performance levels off after threshold 4, demonstrating the algorithm’s reliability at higher thresholds. In contrast, “COVID-2” experiences a minor drop in performance at threshold 6, which may be due to over-segmentation, affecting overall signal quality.

In the case of “Normal-1,” PSNR consistently rises across all threshold levels, indicating ongoing performance improvements with the addition of thresholds. This implies that the algorithm effectively manages simpler image structures by progressively refining segmentation. On the other hand, “Normal-2” achieves its best performance at threshold level 5, followed by a slight decrease at level 6. This pattern suggests that overly detailed segmentation can lead to over-segmentation in certain cases, where finer details may not enhance the overall quality of segmentation. These findings confirm the algorithm’s adaptability to various image types and complexities while also emphasizing the need for careful selection of threshold levels to achieve optimal performance.

**Fig. 13:**
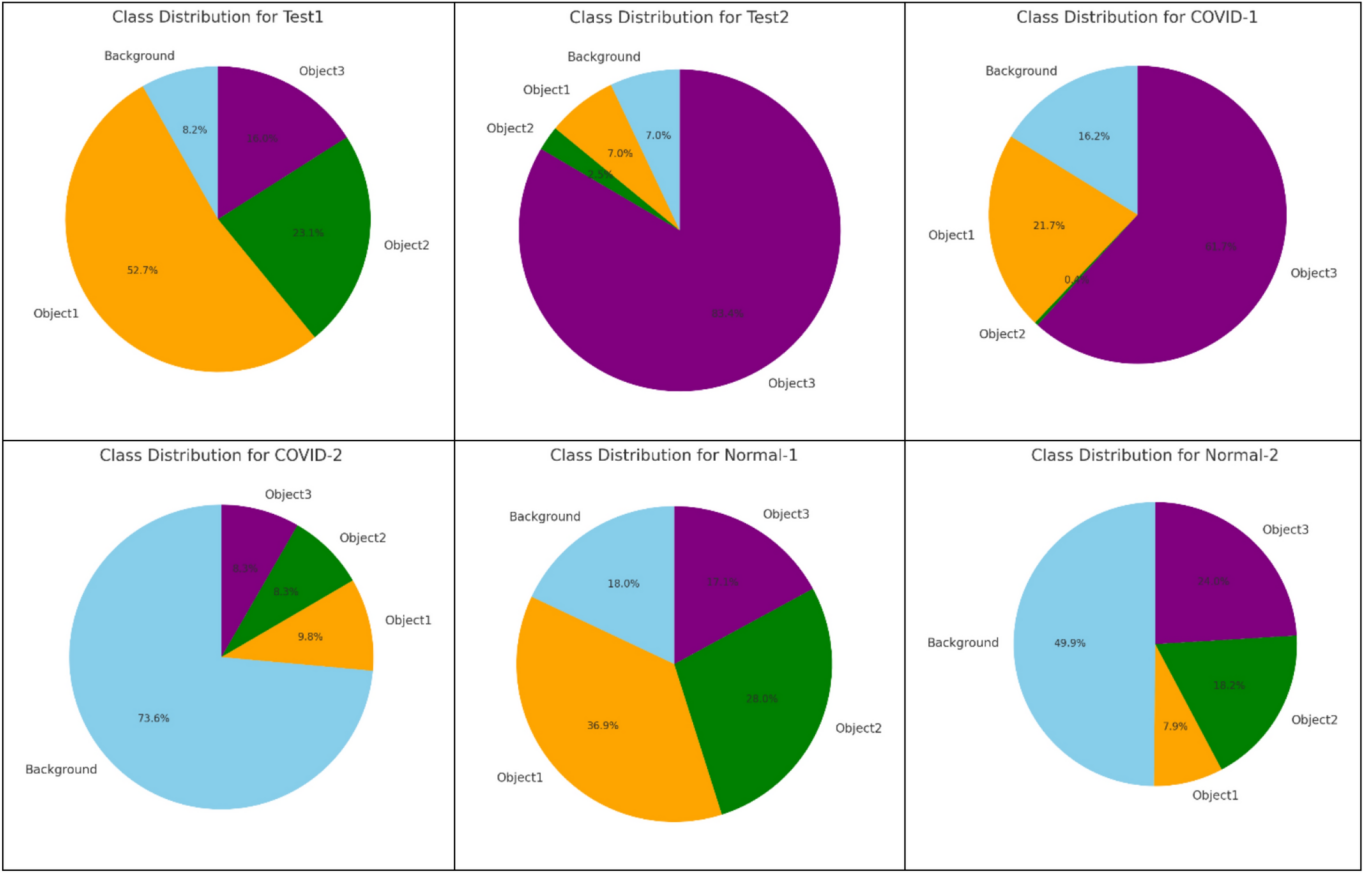
The class distribution plots for simulated segmentation outputs across different categories.

The class distribution plots in Fig. 13 shed light on how well the segmentation algorithm can distinguish and balance different regions within the images. In the cases of “Test1” and “Test2,” the distribution among the classes appears fairly balanced, with the “Background” taking up a large portion. This suggests that these images likely feature significant empty or uniform areas, while the segmentation algorithm successfully captures finer details in the other classes. The nearly equal shares of “Object1” and “Object2” demonstrate the algorithm’s capability to segment distinct features consistently.

For “COVID-1” and “COVID-2,” the “Background” class is predominant, reflecting the structured and less detailed nature of medical images. The algorithm assigns smaller yet noticeable portions to “Object2” and “Object3,” which likely correspond to important features such as lesions or tissues. These plots highlight the necessity of accurately identifying smaller, medically significant regions while effectively managing larger uniform areas.

The images “Normal-1” and “Normal-2” exhibit a more varied distribution among the classes, with a lesser dominance of the “Background” class compared to the “COVID” images. This indicates that the segmentation algorithm faced more complex or intricate patterns in these images, resulting in a more even distribution of regions. The balanced segmentation across “Object1,” “Object2,” and “Object3” emphasizes the algorithm’s flexibility in handling diverse image complexities. These findings affirm the segmentation algorithm’s effectiveness across various image types while offering insights into its performance with differing levels of complexity.

## Conclusion

Image segmentation is a fundamental challenge in image processing and computer vision, serving as the foundation for various applications such as pattern recognition, machine vision, robotic perception, surveillance, augmented reality, and medical imaging. While bi-level thresholding techniques are effective for straightforward image segmentation tasks, complex images featuring multiple objects or colors present significant computational challenges. Multi-level thresholding for color image segmentation becomes a particularly intricate optimization problem due to the delicate balance between computational efficiency and segmentation quality.

This study introduced a novel meta-heuristic approach called RSA to tackle this issue. By utilizing entropy-based objective functions from Otsu and Kapur, RSA was tested on 16 benchmark images, including those related to COVID-19, as well as color and grayscale categories. The performance of RSA was assessed through fitness function optimization, Peak Signal-to-Noise Ratio (PSNR), and Structural Similarity Index (SSIM). A comparative analysis revealed that RSA surpassed several leading methods, highlighting its robustness and adaptability across various image types and segmentation criteria. The experimental findings emphasized RSA’s capability to achieve enhanced segmentation accuracy and computational efficiency. RSA showed consistent performance across different datasets, demonstrating its potential as a dependable solution for multi-level thresholding in image segmentation. Nevertheless, challenges such as improving the algorithm’s balance between exploration and exploitation remain areas for future enhancement.

Some new works can be applied in the future, which are as follows.

- Algorithmic Enhancement: Combine RSA with other optimization techniques, like swarm intelligence or evolutionary strategies, to improve its search capabilities and achieve a better balance between exploration and exploitation. Look into adaptive or dynamic parameter adjustment methods to enhance RSA’s robustness across different datasets.
- Application to Complex Domains: Broaden RSA’s use to tackle more intricate medical imaging challenges, such as tumor segmentation, organ detection, and analyzing disease progression in 3D medical imaging datasets. Examine RSA’s effectiveness in remote sensing for applications like land-use classification, urban planning, and disaster management.
- Testing on Diverse Optimization Problems: Utilize RSA to address issues beyond image segmentation, including text classification, natural language processing, and feature selection in machine learning. Investigate its industrial applications in areas such as task scheduling, resource allocation, and parameter optimization within engineering systems. Assess RSA’s performance in renewable energy forecasting, particularly in predicting wind and solar power, where non-linear optimization problems are common.
- Scalability and Real-Time Applications: Improve the computational efficiency of RSA to manage large-scale datasets and real-time applications in surveillance systems or autonomous vehicles. Create parallel or distributed computing frameworks to enhance RSA’s performance for high-resolution and big-data scenarios.
- Integration with Deep Learning: Explore hybrid methods by merging RSA with deep learning techniques, such as convolutional neural networks (CNNs) or transformer models, to optimize feature selection and boost segmentation accuracy. Investigate how RSA can support deep neural networks in unsupervised learning tasks.
- Cross-Domain Generalization: Examine RSA’s flexibility in solving optimization problems across various fields, such as finance (portfolio optimization), healthcare (personalized treatment plans), and environmental science.

This study positions RSA as a strong basis for addressing multi-level thresholding in image segmentation while also paving the way for further developments in computational optimization and applications across various fields. By investigating these future avenues, we can fully harness RSA’s potential, encouraging innovation in both theoretical studies and real-world applications.

## Data Availability

Data is available from the Laith Abualigah upon reasonable request.

## References

[1] Zhu, Q. Research on road traffic situation awareness system based on image big data. *IEEE Intell. Syst.* **35**, 18–26 (2019).

[2] Smith, J. et al. Robust resource allocation in a cluster based imaging system. *Parallel Comput.* **35**, 389–400 (2009).

[3] Čuš-Babič, N., Rebolj, D., Nekrep-Perc, M. & Podbreznik, P. Supply-chain transparency within industrialized construction projects. *Comput. Ind.* **65**, 345–353 (2014).

[4] Liu, L., Lu, C., Xiao, F., Liu, R. & Xiong, N. N. A practical, integrated multi-criteria decision-making scheme for choosing cloud services in cloud systems. *IEEE Access* **9**, 88391–88404 (2021).

[5] Pham, H. S. T. & Khanh, C. N. T. Ecotourism intention: the roles of environmental concern, time perspective and destination image. *Tourism Review* **76**, 1141–1153 (2021).

[6] Parenti, M., Fossa, M. & Delucchi, L. A model for energy predictions and diagnostics of large-scale photovoltaic systems based on electric data and thermal imaging of the PV fields. *Renew. Sustain. Energy Rev.* **206**, 114858 (2024).

[7] Mall, P. K. et al. A comprehensive review of deep neural networks for medical image processing: Recent developments and future opportunities. *Healthcare Analytics* **1**, 100216 (2023).

[8] Windhager, J. et al. An end-to-end workflow for multiplexed image processing and analysis. *Nat. Protoc.* **18**, 3565–3613 (2023). 37816904 10.1038/s41596-023-00881-0

[9] Pham, M.-V., Ha, Y.-S. & Kim, Y.-T. Automatic detection and measurement of ground crack propagation using deep learning networks and an image processing technique. *Measurement* **215**, 112832 (2023).

[10] Sherif, K. et al. Revolutionizing oil spill detection: A machine learning approach for satellite image classification. In *2024 International Telecommunications Conference (ITC-Egypt)* 245–250 (2024).

[11] Thalji, N. et al. Segmented X-ray image data for diagnosing dental periapical diseases using deep learning. *Data Brief* **54**, 110539 (2024). 38882192 10.1016/j.dib.2024.110539 PMC11177072

[12] Otair, M. et al. Adapted arithmetic optimization algorithm for multi-level thresholding image segmentation: a case study of chest x-ray images. *Multimedia Tools and Applications* **83**, 41051–41081 (2024).

[13] Wang, R. et al. Medical image segmentation using deep learning: A survey. *IET Image Proc.* **16**, 1243–1267 (2022).

[14] Chouhan, S. S., Kaul, A. & Singh, U. P. Image segmentation using computational intelligence techniques. *Archives of Computational Methods in Engineering* **26**, 533–596 (2019).

[15] Feng, Y. et al. GCFormer: Multi-scale feature plays a crucial role in medical images segmentation. *Knowl.-Based Syst.* **300**, 112170 (2024).

[16] Liu, Y., Zhang, Z., Liu, X., Wang, L. & Xia, X. Efficient image segmentation based on deep learning for mineral image classification. *Adv. Powder Technol.* **32**, 3885–3903 (2021).

[17] Chouhan, S. S., Kaul, A. & Singh, U. P. Soft computing approaches for image segmentation: a survey. *Multimedia Tools and Applications* **77**, 28483–28537 (2018).

[18] Olugbara, O. O., Adetiba, E. & Oyewole, S. A. Pixel intensity clustering algorithm for multilevel image segmentation. *Math. Probl. Eng.* **2015**, 649802 (2015).

[19] Bhargavi, K. & Jyothi, S. A survey on threshold based segmentation technique in image processing. *International Journal of Innovative Research and Development* **3**, 234–239 (2014).

[20] Bugeau, A. & Pérez, P. Detection and segmentation of moving objects in complex scenes. *Comput. Vis. Image Underst.* **113**, 459–476 (2009).

[21] Peng, A., Zhang, L. & Zhang, D. A survey of graph theoretical approaches to image segmentation. *Pattern Recogn.* **46**, 1020–1038 (2013).

[22] Wu, Z. & Leahy, R. An optimal graph theoretic approach to data clustering: Theory and its application to image segmentation. *IEEE Trans. Pattern Anal. Mach. Intell.* **15**, 1101–1113 (1993).

[23] Jardim, S., António, J. & Mora, C. Image thresholding approaches for medical image segmentation-short literature review. *Procedia Computer Science* **219**, 1485–1492 (2023).

[24] Zhang, Y.-J. A survey on evaluation methods for image segmentation. *Pattern Recogn.* **29**, 1335–1346 (1996).

[25] R. B. Oliveira, E. Mercedes Filho, Z. Ma, J. P. Papa, A. S. Pereira, and J. M. R. Tavares, “Computational methods for the image segmentation of pigmented skin lesions: a review,” *Computer Methods and Programs in Biomedicine,* 131, 127–141, 2016. 10.1016/j.cmpb.2016.03.032 27265054

[26] Pare, S., Kumar, A., Singh, G. K. & Bajaj, V. Image segmentation using multilevel thresholding: a research review. *Iranian Journal of Science and Technology, Transactions of Electrical Engineering* **44**, 1–29 (2020).

[27] Tobias, O. J. & Seara, R. Image segmentation by histogram thresholding using fuzzy sets. *IEEE Trans. Image Process.* **11**, 1457–1465 (2002). 18249714 10.1109/TIP.2002.806231

[28] Sezan, M. I. A peak detection algorithm and its application to histogram-based image data reduction. *Computer Vision, Graphics, and Image Processing* **49**, 36–51 (1990).

[29] Nakib, A., Oulhadj, H. & Siarry, P. Image histogram thresholding based on multiobjective optimization. *Signal Process.* **87**, 2516–2534 (2007).

[30] Sen, D. & Pal, S. K. Gradient histogram: Thresholding in a region of interest for edge detection. *Image Vis. Comput.* **28**, 677–695 (2010).

[31] Sarkar, S. & Das, S. Multilevel image thresholding based on 2D histogram and maximum Tsallis entropy—a differential evolution approach. *IEEE Trans. Image Process.* **22**, 4788–4797 (2013). 23955760 10.1109/TIP.2013.2277832

[32] Sahoo, P. K., Soltani, S. & Wong, A. K. A survey of thresholding techniques. *Computer Vision, Graphics, and Image Processing* **41**, 233–260 (1988).

[33] N. V. Lopes, P. A. M. do Couto, H. Bustince, and P. Melo-Pinto, “Automatic histogram threshold using fuzzy measures,” *IEEE Transactions on Image Processing,* 19, 199–204, 2009. 10.1109/TIP.2009.2032349 19758860

[34] Chouikhi, N., Ammar, B., Hussain, A. & Alimi, A. M. Bi-level multi-objective evolution of a multi-layered echo-state network autoencoder for data representations. *Neurocomputing* **341**, 195–211 (2019).

[35] Al Aqrabi, H. et al. A multi-layer hierarchical inter-cloud connectivity model for sequential packet inspection of tenant sessions accessing BI as a service. In *2014 IEEE Intl Conf on High Performance Computing and Communications, 2014 IEEE 6th Intl Symp on Cyberspace Safety and Security, 2014 IEEE 11th Intl Conf on Embedded Software and Syst (HPCC, CSS, ICESS)* 498–505 (2014).

[36] Abualigah, L., Al-Okbi, N. K., Awwad, E. M., Sharaf, M. & Daoud, M. S. Boosted aquila arithmetic optimization algorithm for multi-level thresholding image segmentation. *Evol. Syst.* **1**, 1–28 (2024).

[37] Otair, M., Alrawi, A. F., Abualigah, L., Jia, H. & Altalhi, M. Enhancing the quality of compressed images using rounding intensity followed by novel dividing technique. *Multimedia Tools and Applications* **83**, 1753–1786 (2024).

[38] Qadri, A. M., Raza, A., Eid, F. & Abualigah, L. A novel transfer learning-based model for diagnosing malaria from parasitized and uninfected red blood cell images. *Decis. Anal. J.* **9**, 100352 (2023).

[39] Su, H. et al. Multilevel threshold image segmentation for COVID-19 chest radiography: A framework using horizontal and vertical multiverse optimization. *Comput. Biol. Med.* **146**, 105618 (2022). 35690477 10.1016/j.compbiomed.2022.105618 PMC9113963

[40] Kurugollu, F., Sankur, B. & Harmanci, A. E. Color image segmentation using histogram multithresholding and fusion. *Image Vis. Comput.* **19**, 915–928 (2001).

[41] Celik, T. Two-dimensional histogram equalization and contrast enhancement. *Pattern Recogn.* **45**, 3810–3824 (2012).

[42] Liu, Q., Li, N., Jia, H., Qi, Q. & Abualigah, L. A chimp-inspired remora optimization algorithm for multilevel thresholding image segmentation using cross entropy. *Artif. Intell. Rev.* **56**, 159–216 (2023).

[43] Chatzikoumi, E. How to evaluate machine translation: A review of automated and human metrics. *Nat. Lang. Eng.* **26**, 137–161 (2020).

[44] Wang, H., Wu, H., He, Z., Huang, L. & Church, K. W. Progress in machine translation. *Engineering* **18**, 143–153 (2022).

[45] Delon, J., Desolneux, A., Lisani, J.-L. & Petro, A. B. A nonparametric approach for histogram segmentation. *IEEE Trans. Image Process.* **16**, 253–261 (2006). 10.1109/tip.2006.884951 17283783

[46] Reddy, T. A. & Henze, G. P. Parametric and non-parametric regression methods. In *Applied Data Analysis and Modeling for Energy Engineers and Scientists* 355–407 (Springer, 2023).

[47] Goh, T. Y., Basah, S. N., Yazid, H., Safar, M. J. A. & Saad, F. S. A. Performance analysis of image thresholding: Otsu technique. *Measurement* **114**, 298–307 (2018).

[48] Manic, K. S., Priya, R. K. & Rajinikanth, V. Image multithresholding based on Kapur/Tsallis entropy and firefly algorithm. *Indian J. Sci. Technol.* **9**, 89949 (2016).

[49] De Albuquerque, M. P., Esquef, I. A. & Mello, A. G. Image thresholding using Tsallis entropy. *Pattern Recogn. Lett.* **25**, 1059–1065 (2004).

[50] Razzak, M. I. et al. Deep learning for medical image processing: Overview, challenges and the future. In *Classification in BioApps: Automation of Decision Making* 323–350 (2018).

[51] Burke, M., Driscoll, A., Lobell, D. B. & Ermon, S. Using satellite imagery to understand and promote sustainable development. *Science* **371**, 8628 (2021). 10.1126/science.abe8628 33737462

[52] El-Darymli, K., Gill, E. W., Mcguire, P., Power, D. & Moloney, C. Automatic target recognition in synthetic aperture radar imagery: A state-of-the-art review. *IEEE Access* **4**, 6014–6058 (2016).

[53] Feng, D., Wenkang, S., Liangzhou, C., Yong, D. & Zhenfu, Z. Infrared image segmentation with 2-D maximum entropy method based on particle swarm optimization (PSO). *Pattern Recogn. Lett.* **26**, 597–603 (2005).

[54] Abualigah, L. et al. Improved reptile search algorithm by salp swarm algorithm for medical image segmentation. *J. Bionic Eng.* **20**, 1766–1790 (2023). 10.1007/s42235-023-00332-2 PMC9902839 36777369

[55] Liu, D. & Yu, J. Otsu method and K-means. In *2009 Ninth International Conference on Hybrid Intelligent Systems* 344–349 (2009).

[56] Sharma, A., Chaturvedi, R., Dwivedi, U., Kumar, S. & Reddy, S. Firefly algorithm based effective gray scale image segmentation using multilevel thresholding and entropy function. *Int. J. Pure Appl. Math.* **118**, 437–443 (2018).

[57] Sneddon, R. The Tsallis entropy of natural information. *Physica A* **386**, 101–118 (2007).

[58] Ss, V. C. & Hs, A. Nature inspired meta heuristic algorithms for optimization problems. *Computing* **104**, 251–269 (2022).

[59] Hussain, K., Mohd Salleh, M. N., Cheng, S. & Shi, Y. Metaheuristic research: A comprehensive survey. *Artif. Intell. Rev.* **52**, 2191–2233 (2019).

[60] Sharma, M. & Kaur, P. A comprehensive analysis of nature-inspired meta-heuristic techniques for feature selection problem. *Arch. Comput. Methods Eng.* **28**, 1103–1127 (2021).

[61] Kar, A. K. Bio inspired computing–a review of algorithms and scope of applications. *Expert Syst. Appl.* **59**, 20–32 (2016).

[62] El-Kenawy, E.-S.M. et al. Novel meta-heuristic algorithm for feature selection, unconstrained functions and engineering problems. *IEEE Access* **10**, 40536–40555 (2022).

[63] Črepinšek, M., Liu, S.-H. & Mernik, M. Exploration and exploitation in evolutionary algorithms: A survey. *ACM Comput. Surv.* **45**, 1–33 (2013).

[64] Chen, J., Xin, B., Peng, Z., Dou, L. & Zhang, J. Optimal contraction theorem for exploration–exploitation tradeoff in search and optimization. *IEEE Trans. Syst. Man Cybern. A Syst. Hum.* **39**, 680–691 (2009).

[65] Blekos, K. et al. A review on quantum approximate optimization algorithm and its variants. *Phys. Rep.* **1068**, 1–66 (2024).

[66] Lateef Haroon, A. et al. An optimized system for sensor ontology meta-matching using swarm intelligent algorithm. *Internet Technol. Lett.* **1**, e498 (2024).

[67] Dehghani, M., Trojovská, E. & Trojovský, P. A new human-based metaheuristic algorithm for solving optimization problems on the base of simulation of driving training process. *Sci. Rep.* **12**, 9924 (2022). 35705720 10.1038/s41598-022-14225-7 PMC9200810

[68] Bäck, T. & Schwefel, H.-P. An overview of evolutionary algorithms for parameter optimization. *Evol. Comput.* **1**, 1–23 (1993).

[69] Lambora, A. et al. Genetic algorithm—A literature review. In *2019 International Conference on Machine Learning, Big Data, Cloud and Parallel Computing (COMITCon)* 380–384 (2019).

[70] Gandomi, A. H. & Alavi, A. H. Krill herd: A new bio-inspired optimization algorithm. *Commun. Nonlinear Sci. Numer. Simul.* **17**, 4831–4845 (2012).

[71] Črepinšek, M., Liu, S.-H. & Mernik, L. A note on teaching–learning-based optimization algorithm. *Inf. Sci.* **212**, 79–93 (2012).

[72] Rashedi, E., Nezamabadi-Pour, H. & Saryazdi, S. GSA: A gravitational search algorithm. *Inf. Sci.* **179**, 2232–2248 (2009).

[73] Bingley, W. J. et al. Enlarging the model of the human at the heart of human-centered AI: A social self-determination model of AI system impact. *New Ideas Psychol.* **70**, 101025 (2023).

[74] Abualigah, L., Abd Elaziz, M., Sumari, P., Geem, Z. W. & Gandomi, A. H. Reptile Search Algorithm (RSA): A nature-inspired meta-heuristic optimizer. *Expert Syst. Appl.* **191**, 116158 (2022).

[75] Žeger, I., Grgic, S., Vuković, J. & Šišul, G. Grayscale image colorization methods: Overview and evaluation. *IEEE Access* **9**, 113326–113346 (2021).

[76] Banerji, S., Sinha, A. & Liu, C. New image descriptors based on color, texture, shape, and wavelets for object and scene image classification. *Neurocomputing* **117**, 173–185 (2013).

[77] Guo, Q. & Peng, H. A novel multilevel color image segmentation technique based on an improved firefly algorithm and energy curve. *Evol. Syst.* **14**, 685–733 (2023).

[78] Wu, B., Zhou, J., Ji, X., Yin, Y. & Shen, X. An ameliorated teaching–learning-based optimization algorithm based study of image segmentation for multilevel thresholding using Kapur’s entropy and Otsu’s between class variance. *Inf. Sci.* **533**, 72–107 (2020).

[79] Houssein, E. H., Abdalkarim, N., Hussain, K. & Mohamed, E. Accurate multilevel thresholding image segmentation via oppositional snake optimization algorithm: Real cases with liver disease. *Comput. Biol. Med.* **169**, 107922 (2024). 38184861 10.1016/j.compbiomed.2024.107922

[80] Panda, R., Samantaray, L., Das, A., Agrawal, S. & Abraham, A. A novel evolutionary row class entropy based optimal multi-level thresholding technique for brain MR images. *Expert Syst. Appl.* **168**, 114426 (2021).

[81] Khehra, B. S. & Pharwaha, A. P. S. Digital mammogram enhancement using Kapur measure of entropy and mathematical morphology. *Biomed. Eng. Appl. Basis Commun.* **25**, 1350029 (2013).

[82] Sathya, P., Kalyani, R. & Sakthivel, V. Color image segmentation using Kapur, Otsu and minimum cross entropy functions based on exchange market algorithm. *Expert Syst. Appl.* **172**, 114636 (2021).

[83] Emam, M. M., Houssein, E. H. & Ghoniem, R. M. A modified reptile search algorithm for global optimization and image segmentation: Case study brain MRI images. *Comput. Biol. Med.* **152**, 106404 (2023). 36521356 10.1016/j.compbiomed.2022.106404

[84] Sasmal, B., Hussien, A. G., Das, A., Dhal, K. G. & Saha, R. Reptile search algorithm: Theory, variants, applications, and performance evaluation. *Arch. Comput. Methods Eng.* **31**, 521–549 (2024).

[85] Yuan, Q., Zhang, Y., Dai, X. & Zhang, S. A modified reptile search algorithm for numerical optimization problems. *Comput. Intell. Neurosci.* **2022**, 9752003 (2022). 36262616 10.1155/2022/9752003 PMC9576354

[86] Abualigah, L. & Diabat, A. Chaotic binary reptile search algorithm and its feature selection applications. *J. Ambient. Intell. Humaniz. Comput.* **14**, 13931–13947 (2023).

[87] Chauhan, S., Vashishtha, G. & Kumar, A. Approximating parameters of photovoltaic models using an amended reptile search algorithm. *J. Ambient. Intell. Humaniz. Comput.* **14**, 9073–9088 (2023).

[88] Barrera-García, J. et al. Enhancing reptile search algorithm performance for the knapsack problem with integration of chaotic map. In *Mexican International Conference on Artificial Intelligence* 70–81 (2024).

[89] Elashry, S. S., Abohamama, A., Abdul-Kader, H. M., Rashad, M. & Ali, A. F. A chaotic reptile search algorithm for energy consumption optimization in wireless sensor networks. *IEEE Access* **1**, 1 (2024). 10.1038/s41598-025-96966-9 PMC12033330 40287489

[90] Couceiro, M. et al. *Particle Swarm Optimization* (Springer, 2016).

[91] Zhu, H., Wang, Y., Wang, K. & Chen, Y. Particle swarm optimization (PSO) for the constrained portfolio optimization problem. *Expert Syst. Appl.* **38**, 10161–10169 (2011).

[92] Marini, F. & Walczak, B. Particle swarm optimization (PSO). A tutorial. *Chemom. Intell. Lab. Syst.* **149**, 153–165 (2015).

[93] Shami, T. M. et al. Particle swarm optimization: A comprehensive survey. *IEEE Access* **10**, 10031–10061 (2022).

[94] Jiang, Y., Hu, T., Huang, C. & Wu, X. An improved particle swarm optimization algorithm. *Appl. Math. Comput.* **193**, 231–239 (2007).

[95] Abdel-Basset, M., Mohamed, R. & Abouhawwash, M. Hybrid marine predators algorithm for image segmentation: Analysis and validations. *Artif. Intell. Rev.* **55**, 3315–3367 (2022). 10.1007/s10462-022-10157-w PMC8935268 35342218

[96] Salgotra, R., Singh, U., Singh, S., Singh, G. & Mittal, N. Self-adaptive salp swarm algorithm for engineering optimization problems. *Appl. Math. Model.* **89**, 188–207 (2021).

[97] Ma, G. & Yue, X. An improved whale optimization algorithm based on multilevel threshold image segmentation using the Otsu method. *Eng. Appl. Artif. Intell.* **113**, 104960 (2022).

[98] Omran, M. G., Salman, A. & Engelbrecht, A. P. Dynamic clustering using particle swarm optimization with application in image segmentation. *Pattern Anal. Appl.* **8**, 332–344 (2006).

[99] Subha, B., Jeyakumar, V. & Deepa, S. Gaussian aquila optimizer based dual convolutional neural networks for identification and grading of osteoarthritis using knee joint images. *Sci. Rep.* **14**, 7225 (2024). 38538646 10.1038/s41598-024-57002-4 PMC11349978

[100] Horé, A. & Ziou, D. Is there a relationship between peak-signal-to-noise ratio and structural similarity index measure?. *IET Image Proc.* **7**, 12–24 (2013).

[101] Bakurov, I., Buzzelli, M., Schettini, R., Castelli, M. & Vanneschi, L. Structural similarity index (SSIM) revisited: A data-driven approach. *Expert Syst. Appl.* **189**, 116087 (2022).

[102] López-Vázquez, A. & Hochsztain, E. Extended and updated tables for the Friedman rank test. *Commun. Stat. Theory Methods* **48**, 268–281 (2019).

